# DNA origami vaccine (DoriVac) nanoparticles improve both humoral and cellular immune responses to infectious diseases

**DOI:** 10.1101/2023.12.29.573647

**Published:** 2024-01-02

**Authors:** Yang C. Zeng, Olivia J. Young, Longlong Si, Min Wen Ku, Giorgia Isinelli, Anjali Rajwar, Amanda Jiang, Chris M. Wintersinger, Amanda R. Graveline, Andyna Vernet, Melinda Sanchez, Ju Hee Ryu, Ick Chan Kwon, Girija Goyal, Donald E. Ingber, William M. Shih

**Affiliations:** 1 Department of Cancer Biology, Dana-Farber Cancer Institute, Harvard Medical School, Boston, Massachusetts 02115, USA; 2 Wyss Institute for Biologically Inspired Engineering at Harvard University, Boston, Massachusetts 02115, USA; 3 Department of Biological Chemistry and Molecular Pharmacology, Harvard Medical School, Boston, Massachusetts 02115, USA; 4 Harvard-Massachusetts Institute of Technology (MIT) Division of Health Sciences and Technology, Massachusetts Institute of Technology, Cambridge, Massachusetts, 02139, USA; 5 Center for Theragnosis, Biomedical Research Institute, Korea Institute of Science and Technology (KIST), Seoul 02792, Republic of Korea; 6 KU-KIST Graduate School of Converging Science and Technology, Korea University, Seoul 02841, Republic of Korea; 7 Vascular Biology Program, Department of Surgery, Boston Children’s Hospital and Harvard Medical School, Boston, MA, USA; 8 Harvard John A. Paulson School of Engineering and Applied Sciences, Cambridge, MA, USA

## Abstract

Current SARS-CoV-2 vaccines have demonstrated robust induction of neutralizing antibodies and CD4^+^ T cell activation, however CD8^+^ responses are variable, and the duration of immunity and protection against variants are limited. Here we repurposed our DNA origami vaccine platform, DoriVac, for targeting infectious viruses, namely SARS-CoV-2, HIV, and Ebola. The DNA origami nanoparticle, conjugated with infectious-disease-specific HR2 peptides, which act as highly conserved antigens, and CpG adjuvant at precise nanoscale spacing, induced neutralizing antibodies, Th1 CD4^+^ T cells, and CD8^+^ T cells in naïve mice, with significant improvement over a bolus control. Pre-clinical studies using lymph-node-on-a-chip systems validated that DoriVac, when conjugated with antigenic peptides or proteins, induced promising cellular immune responses in human cells. These results suggest that DoriVac holds potential as a versatile, modular vaccine platform, capable of inducing both humoral and cellular immunities. The programmability of this platform underscores its potential utility in addressing future pandemics.

## Background

The coronavirus disease 2019 pandemic highlighted the need for swift vaccine development. Initial focus on rapid vaccine design for pandemics^1–5^ led to the novel mRNA vaccines, mRNA-1273 (manufactured by Moderna) and BNT162b2 (manufactured by Pfizer), which rely on lipid nanoparticle delivery of mRNA encoding an early variant of the spike protein. Despite their success, the emergence of SARS-CoV-2 variants like B.1.351 (Beta)^6^, B.1.617.2 (Delta)^7^, and B.1.529 (Omicron)^8^ raised concerns about the vaccine efficacy as variants demonstrated the ability to evade immunity^9–15^. The immune evasion observed with current vaccines necessitates interventions effective against mutations.

Current SARS-CoV-2 vaccines focus on the receptor binding domain (RBD) of the spike protein. Viruses rely on RBD to bind to cells and initiate infection, and then heptad repeat 1 (HR1) and heptad repeat 2 (HR2) to fuse the viral and cell membranes. HR1 and HR2, conserved across various viruses, self-assemble into α-helical coils, and then assemble into superhelical structures to facilitate fusion^16–21^. While the RBD region and other viral regions are subject to viral evolution, HR1 and HR2 sequences remain highly conserved, providing a conserved antigen for vaccines^22^. Only three amino acids differ between the original SARS-CoV-2 HR1 sequence and the Omicron variant (Supplementary Table 1). HR1 and HR2 also harbor T cell epitopes and may induce neutralizing antibodies, serving as viable antigens for vaccine design^23,24^. HR2, with a simpler structure than HR1, has been successfully targeted by vaccines,^22,25^ and was selected as the antigen for delivery via our vaccine platform for SARS-CoV-2, HIV, and Ebola (Supplementary Table 2).

While the vaccine community traditionally prioritized neutralizing antibody responses^26^, there is now growing acknowledgement of the essential role of cellular immune responses (dendritic cells, CD4^+^ and CD8^+^ T cells) for broad viral protection^9,24,27–33^. Functional T cells prevent immune escape of mutated strains^9^. SARS-CoV-2 mutated strains have been demonstrated to escape neutralizing antibody responses, but not T cell responses^34^. CD4^+^ T cells support antibody generation^35^, and studies show that CD4^+^ T cell transfer can protect against viral challenge^36^. Mild SARS-CoV-2 infections exhibit robust CD8^+^ T cell reactivity^37,38^ contributing to rapid viral clearance^29^. Depleting CD8^+^ T cells in non-human primates increases susceptibility to SARS-CoV-2 re-infection^39^. In HIV and Ebola, CD8^+^ T cells are crucial for long-term control and vaccine-induced protection. CD8^+^ depletion led to failure in controlling simian immunodeficiency virus in non-human primates^40,41^. In Ebola, CD8^+^ cells were essential for immune protection in non-human primates, while antibody transfer failed to protect^42^. An ideal vaccine should induce both humoral and cellular immune responses, including neutralizing antibodies and long-term memory T cells^9^. mRNA vaccines demonstrate robust CD4^+^ responses, but variable CD8^+^ responses; both influence long-term immunity^43–47^.

Multiple vaccine candidates have been developed to induce neutralizing antibodies and cellular responses against the SARS-CoV-2 spike protein, including over 60 different nanoparticle formulations^48,49^. Despite the success of lipid nanoparticle-based mRNA vaccines, these vaccines face challenges like manufacturing complexity, cold-chain requirements, limited stability, high cost, poor cargo loading efficiency, limited control over cargo stoichiometry, and off-target effects^48,50^. Our goal is to introduce DoriVac, a DNA origami nanoparticle vaccine platform, as a versatile platform for infectious disease. While previous studies have demonstrated vaccine delivery with DNA origami for cancer, this study aims to demonstrate its broad applicability for infectious diseases. DoriVac induced robust humoral and T cell immune responses against SARS-CoV-2, HIV, and Ebola viruses in mouse models, demonstrating the platform’s programmability for various infectious-disease HR2 antigens. This approach may broadly apply to pathogen vaccine development by conjugating the respective antigens to the DNA origami.

## Fabrication of modular DoriVac nanoparticles

**Figure 1.**
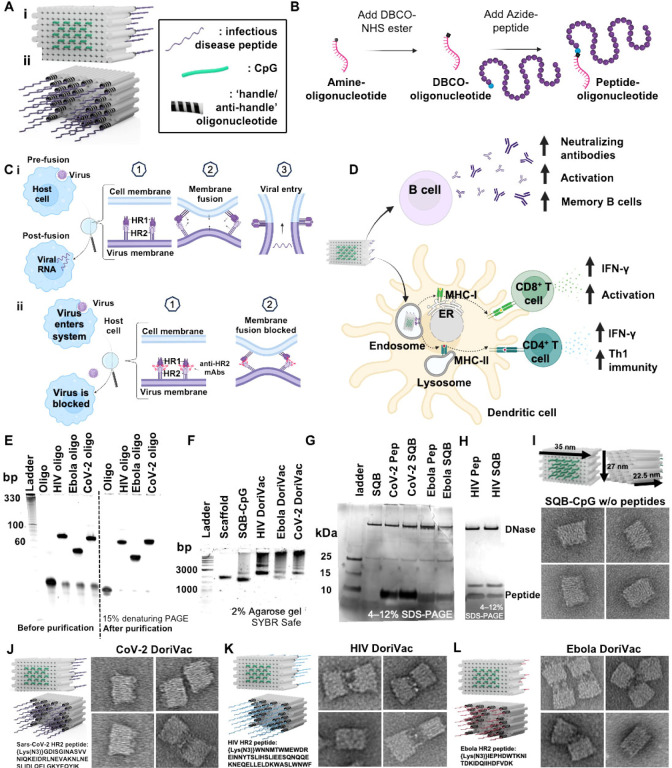
DNA origami vaccines (DoriVac) were fabricated with infectious-disease-specific peptides. **(A) S**chematic of DoriVac, consisting of a DNA origami square block (SQB) nanoparticle conjugated with CpG at precise spacing of 3.5 nm and with infectious disease-specific peptides. **(B)** Schematic demonstrated conjugation of DBCO-modified-oligonucleotide to an azide-modified peptide via copper-free click chemistry. **(C) (i)** A schematic showing how HR2 protein mediates virus-host fusion; HR2 can serve as a conserved target for infectious-disease vaccines. **(ii)** Schematic showing how production of anti-HR2 antibodies (mAbs) prevents virus-host cell-membrane fusion and thereby inhibits viral infection. **(D) S**chematic of DNA origami SQB nanoparticles delivering antigen and adjuvant at a precise spacing to antigen presenting cells, eliciting both humoral and cellular immune responses. **(E)** Denaturing PAGE gel demonstrating successful conjugation and purification of infectious-disease-specific HR2 peptides to anti-handle oligonucleotides (“oligo”). **(F)** Agarose gel demonstrating successful conjugation of oligo-HR2 peptides to the SQB DNA origami nanoparticle. **(G–H)** SDS-PAGE gel demonstrating the results after DNaseI digestion of infectious disease-specific HR2 peptides alone or conjugated with SQBs. DNaseI digestion of the SQBs, followed by the analysis of the conjugated peptides using silver staining, confirms the successful peptide conjugation on the SQBs. **(I–L)** Proposed schematics of the SQBs conjugated with CpGs (SQB-CpG) and SQBs conjugated with CpGs and HR2 peptides (CoV-2-HR2 DoriVac, HIV-HR2 DoriVac, Ebola-HR2 DoriVac), respectively, and their representative TEM images. The specific HR2 peptide sequences associated with each infectious disease are listed.

We previously developed a DNA origami nanoparticle, termed square block (SQB), for its square-lattice architecture for precise spatial presentation of CpG adjuvants^55^. Formed through self-assembly of a long scaffold strand with corresponding short ‘staple’ strands, DoriVac is easy to manufacture and highly stable without cold-chain requirements, exhibits high cargo-loading efficiency due to the robustness of DNA hybridization, and offers precise control over cargo attachment. This platform facilitates optimized spatial arrangement of immune-activating adjuvants, resulting in robust cellular immune responses in various cancers, as previously published^55^. The SQB flat face, modified with 18 CpG strands at 3.5 nm spacing, induces type I (Th1) skewed immune activation (Fig. 1Ai)^55^.

We applied DoriVac technology to create vaccines for SARS-CoV-2, HIV, and Ebola viruses by linking highly conserved viral HR2 peptides to the extruding face of the SQB nanoparticles (Fig. 1Aii). HR2 peptides contain MHC-I and MHC-II epitopes, which are crucial for broadly activating cellular immunity. To this end, we designed peptide-oligonucleotide conjugates with the appropriate “anti-handle” strand through DBCO-Azide click chemistry for specific attachment onto 24 specific “handle” sites of the extruding face of the SQB (Fig. 1A, B). The SQB nanoparticle co-delivers CpG adjuvant and disease-specific HR2 antigens to antigen presenting cells. B cells produce neutralizing antibodies, which can block the membrane fusion of the virus with the host cell (Fig. 1C). Dendritic cells (DCs) present and cross-present the antigens to activate both CD4^+^ and CD8^+^ T cells (Fig. 1D). The oligonucleotide-HR2-peptide conjugates were purified via PAGE purification (Fig. 1E). The agarose-gel electrophoresis band shift demonstrates successful fabrication of peptide-functionalized SQB (Fig. 1F). To confirm peptide conjugation efficiency to the SQB, we digested the DNA origami via DNase I and estimated peptide occupancy of greater than 95% of the conjugation sites via silver stain (Fig. 1G–H, Supplementary Table 3). Fabrication of SARS-CoV-2-HR2, HIV-HR2, and Ebola-HR2 DoriVac was verified via TEM (Fig. 1I–L, Supplementary Figure 1). Aggregation was observed via agarose gel, especially in the case of the HIV and Ebola SQBs of which the majority are dimers, possibly due to hydrophobic peptide interactions.

## DoriVac induces robust humoral immune responses

**Figure 2.**
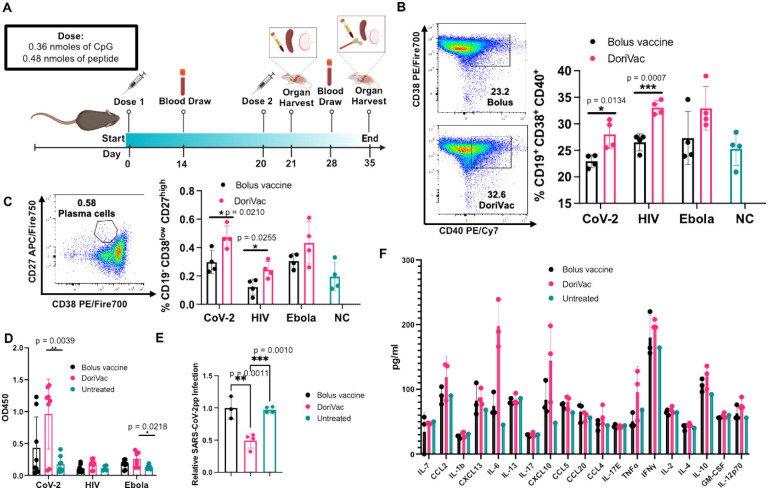
Immune profiling reveals the DoriVac elicits improved neutralizing antibody responses compared to a bolus vaccine. **(A)** Schematic delineating the vaccine administration protocol for naïve C57BL/6 mice and the data collection timeline. Lymph nodes (LNs) and spleens were collected on day 21 and day 35 after sacrificing the mice for flow cytometry and ELISpot analysis. Plasma was collected on Days 14 and 28 for anti-HR2 antibody quantification and pseudovirus neutralization assays. Bone marrow and heart blood was collected at the conclusion of the study, day 35, to analyze B cell markers. **(B)** B cells collected from the blood demonstrated increased markers of activation and antigen-presentation capabilities (CD40) and memory capacity (CD38) after two doses of DoriVac treatment (n=4) as determined by flow cytometry on day 21. NC refers to negative control. **(C)** B cells collected from the blood on Day - 21 demonstrated increased plasma-memory-cell population as evidenced by the increased CD19^−^ CD38^low^ CD27^high^ subpopulation as determined by flow cytometry (n=4). **(D)** DoriVac treatment enhanced HR2-specific IgG antibody production as evidenced via ELISA assay, after two doses of DoriVac (on Day 35) compared to a bolus vaccine of free peptide and free CpG. Samples were diluted 1:100 before quantification. Data has been normalized (n=8). **(E)** SARS-CoV-2 pseudovirus (SARS-CoV-2pp) neutralization assay (n=3–4, 1:10 dilution, Day 28) in model cell line ACE2–293T (n=4). **(F)** Plasma was collected four hours after the first treatment dose on Day 0. The inflammatory cytokine response was quantified via Luminex ELISA assay (Bio-Plex Pro Mouse Cytokine 20-plex Assay (Bio-Rad)) (n=3 for treated groups; n=1 for negative (i.e. untreated) control). Data are represented as mean ± SD. The pseudovirus and ELISA data were analyzed by one-way ANOVA (with correction for multiple comparisons using a Tukey test) and significance was defined as a multiplicity-adjusted p value less than 0.05. The flow data were analyzed by multiple unpaired t-tests and significance was defined as a two-tailed p value less than 0.05. ‘*’ refers to p≤ 0.05; ‘**’ refers to p≤ 0.01; ‘***’ refers to p ≤ 0.001.

Having successfully fabricated the vaccine, we evaluated DoriVac’s efficacy for induction of both humoral and cellular immune responses *in vivo*. Naïve mice were administered 20 pmol of HR2-fabricated DoriVac, comprising 0.36 nmol (2.2 μg) of CpG and 0.48 nmol of antigen (1.5 – 3.2 μg) (Fig. 2A). Two subcutaneous doses of DoriVac were given on day 0 and day 20, compared to bolus vaccine consisting of free CpG adjuvant and HR2 peptide. Blood samples were collected on day 14 and 28 for peripheral blood mononuclear cells (PBMCs) and plasma processing. On day 21, half of the mice from each group were sacrificed for immune cell profiling (Supplementary Table 4–6). On day 35, the remaining mice were sacrificed for immune cell profiling.

On day 21, B cells from PBMCs exhibited increased CD40 expression, a marker of activation and antigen-presentation capacity, in all three DoriVac treatment groups (Fig. 2B), surpassing the bolus vaccine, suggesting that DoriVac is superior in inducing B cell activation. Day 35 revealed a heightened plasma memory B cell population in the bone marrow, as evidenced by an increased CD19^low^ CD38^low^ CD27^high^ subpopulation after DoriVac treatment (Fig. 2C), despite unchanged overall B cell numbers (Supplementary Figures 2–4). SARS-CoV-2-HR2-DoriVac treatment induced elevated HR2 peptide-specific IgG1 antibody responses, as quantified via ELISA, compared to the bolus vaccine (Fig. 2D, Supplementary Figure 4). Neutralizing antibodies harvested from SARS-CoV-2-HR2-DoriVac groups significantly reduced infection in a SARS-CoV-2 pseudovirus (SARS-CoV-2pp) assay (Fig. 2E). In contrast, we did not observe neutralization of the pseudovirus for HIV and Ebola in our assay, possibly due to the weak immunogenicity of the antigens associated with these viruses (Supplementary Tables 7–14). We did observe modest antigen-specific IgG1 responses for HIV and Ebola after HIV-HR-DoriVac and Ebola-HR2-DoriVac treatment, respectively (Fig. 2D, Supplementary Figure 4). We examined initial cytokine responses four hours post the first vaccine dose to naïve mice (Fig. 2F). Type 1 cytokines (TNFα, IL-2, IFNγ, IL-12) were slightly elevated, while type 2 cytokines (IL-4, IL-10) exhibited no obvious elevation after DoriVac treatment compared to the bolus vaccine group and the untreated mice^56^. Overall, these findings affirm DoriVac’s superior induction of humoral immune responses compared to those induced by a bolus vaccine, demonstrating its effectiveness in reducing the infection rate of SARS-CoV-2 pseudovirus.

## DoriVac induces DC activation

**Figure 3.**
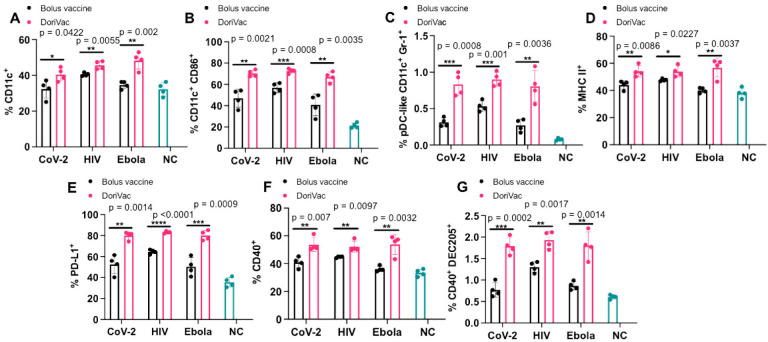
Immune profiling reveals DoriVac elicits superior antigen presenting cell responses compared to a bolus vaccine. C57BL/6 mice were treated with DoriVac (20 pmol) on Day 0 and Day 20. The mice were sacrificed on Day 21 and the draining lymph nodes (LNs) were processed into single-cell suspensions and analyzed by flow cytometry. **(A)** Percentages of CD11c^+^ cells in the draining LNs (n=4) were quantified. NC means negative (i.e. untreated) control. **(B)** Percentages of CD11c^+^ CD86^+^ DCs in the draining LNs (n=4) as determined by flow cytometry. **(C)** Percentages of human plasmacytoid DC (pDC)-like (CD11c^+^ Gr-1^+^) DCs in the draining LNs (n=4) as determined by flow cytometry. **(D)** Percentages of MHC-II^+^ DCs in the draining LNs (n=4) as determined by flow cytometry. **(E)** Percentages of PD-L1^+^ population in the draining LNs (n=4) as determined by flow cytometry. **(F)** Percentages of CD40^+^ population in the draining LNs (n=4) as determined by flow cytometry. **(G)** Percentages of CD40^+^ DEC205^+^ population in DCs in the draining LNs (n=4) as determined by flow cytometry. DoriVac demonstrated a significant increase in DC activation compared to bolus-vaccine treatment. Data are represented as mean ± SD. The flow data were analyzed by multiple unpaired t-tests and significance was defined as a two-tailed p value less than 0.05. ‘*’ refers to P ≤ 0.05; ‘**’ refers to P ≤ 0.01; ‘***’ refers to P ≤ 0.001; ‘****’ refers to P ≤ 0.0001.

To ensure enduring immune protection against viral variants, a vaccine should stimulate both humoral and cellular immune responses. We first checked the DCs, which serve as a link between innate and adaptive immune responses. On day 21 — one day after the second vaccine dose— half of the mice were sacrificed, and the draining lymph nodes near the injection site were collected for flow (Supplementary Figure 5). DoriVac increased the overall DC population (Fig. 3A) and activated DCs (CD11c^+^ CD86^+^) compared to the bolus vaccine (Fig. 3B). Plasmacytoid DCs (pDCs) are crucial in the anti-viral response in humans, secreting abundant type-1 interferon, fostering T cell activation and recruiting other immune cells^57^. The human pDC-like population (CD11c^+^ Gr-1^+^) significantly increased after DoriVac treatment (Fig. 3C), suggesting an increased anti-viral response. Activation markers MHC-II, PD-L1 and CD40 also increased after DoriVac administration (Fig. 3D–F). A notable rise was observed in the CD40^+^ DEC205^+^ DC population, indicating an increase in the activated, endocytic DC population (Fig. 3G). These results demonstrated DoriVac induced robust activation of DCs in healthy mice (Supplementary Figure 6). Furthermore, co-delivery of SQB and HR2 peptide did not elicit the same level of DC activation as observed with the delivery of HR2 peptide-conjugated SQBs (Supplementary Figure 7). This observation is consistent with the outcomes previously noted in DoriVac studies involving tumor-bearing mice^55^, further supporting the notion that the enhanced efficacy of the conjugated delivery system in activating DCs.

## DoriVac demonstrates activation of CD4^+^ T cells

**Figure 4.**
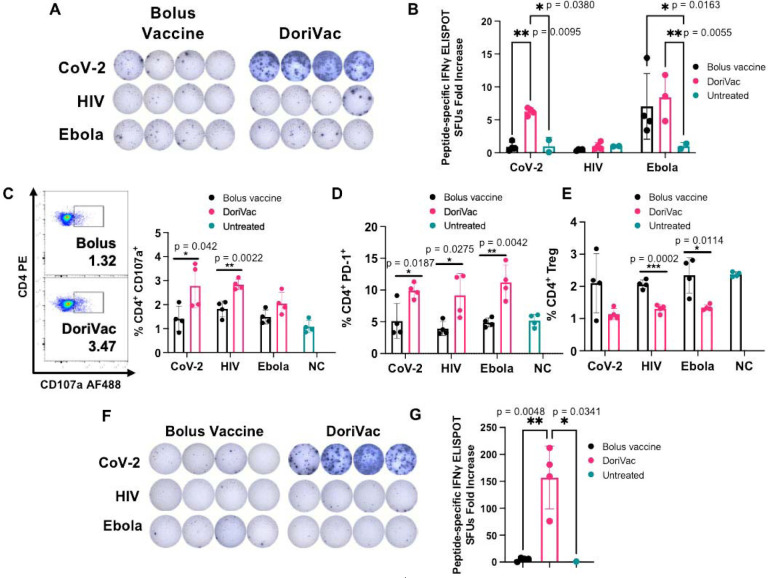
DoriVac induces enhanced Th1 CD4^+^ T cell activation in mice. **(A)** IFNγ ELISpot demonstrating frequency of antigen-specific T cells in splenocytes on day 35 (n=4) after treatment with DoriVac compared with the bolus vaccine. **(B)** Quantification of IFNγ ELISpot spot forming units (SFUs) demonstrates significant increase in SARS-COV-2 antigen-specific T cell frequency after treatment with DoriVac compared with the bolus vaccine and negative (i.e. untreated) control. **(C)** Percentages of CD4^+^ CD107a^+^ T cells in the LN (n=4) as determined by flow cytometry and representative flow plots on day 35. NC means negative (i.e. untreated) control. **(D)** Percentages of the LN CD4^+^ PD-1^+^ population (n=4) as determined by flow cytometry on day 21. **(E)** Percentages of LN CD4^+^ T regulatory cell (Treg) population (n=4) as determined by flow cytometry on day 21. **(F)** IFNγ ELISpot demonstrating frequency of CD4^+^ enriched antigen-specific splenocytes (n=4, day 35) after treatment with SARS-CoV-2-HR2 DoriVac. **(G)** Corresponding quantification of IFNγ ELISpot spot forming units (SFUs). Data are represented as mean ± SD. The ELIspot data in (B) were analyzed by two-way ANOVA (with correction for multiple comparisons using Tukey’s test). The ELISpot data in (G) were analyzed by one-way ANOVA (with correction for multiple comparisons using Tukey’s test). In both analyses, statistical significance was defined as a multiplicity-adjusted p value less than 0.05. The flow data were analyzed by multiple unpaired t-tests and significance was defined as a two-tailed p value less than 0.05. ‘*’ refers to P ≤ 0.05; ‘**’ refers to P ≤ 0.01.

In our previous study involving DoriVac in mouse cancer models^55^, we showed that DC activation by DoriVac leads to broad T cell activation. We aimed to confirm T cell induction by DoriVac in the context of viral antigens. Antigen-specific T cell activation was assessed via IFNγ ELISpot assay on splenocytes; our results demonstrated a significant increase in antigen-specific T cells after SARS-CoV-2-HR2 DoriVac administration (Fig. 4A, B). In contrast, HIV-HR2 DoriVac administration led to only a modest increase, and Ebola-HR2 DoriVac showed no apparent effect (Fig. 4A, B; Supplementary Figure 8). We attribute this to the limited immunogenicity of the HR2 peptides used for HIV and Ebola, validated by their weaker predicted binding to MHC-I and MHC-II in both mice and humans via NetMHCpan-4.1^58^. In contrast, the SARS-CoV-2 HR2 peptide exhibited stronger binding predictions with six epitopes classified as strong binders, compared to zero to two epitopes for HIV and Ebola (Supplementary Tables 7–10). These findings underscore the importance of antigen selection in achieving effective antigen-specific T cell activation. Notably, these results confirmed that SARS-CoV-2-HR2 DoriVac induces significantly more antigen-specific T cells compared to the bolus vaccine.

Beyond overall T cell responses, we confirmed the presence of activated Th1 CD4^+^ T cells via flow cytometry (Supplementary Figure 9 for gating strategy). CD107a notably increased in the CD4^+^ T cell population, indicating enhanced CD4^+^ T cell activation and increased cytotoxic potential (Fig. 4C)^59^. PD-1 was also upregulated in the CD4^+^ T cell population, demonstrating increased activation (Fig. 4D). The T regulatory cell (Treg) population significantly decreased after treatment with DoriVac, suggesting reduced immunosuppression (Fig. 4E). Antigen-specific CD4^+^ T cell activation (CD8^+^ T cells depleted by positive sorting) was quantified via IFNγ ELISpot, revealing a significant increase in antigen-specific activation after SARS-CoV-2 vaccination (Fig. 4F, G). These results verified activation of CD4^+^ T cells, demonstrating an antigen-specific immune response critical for immune memory.

## DoriVac induces an antigen-specific CD8^+^ T cell activation

**Figure 5.**
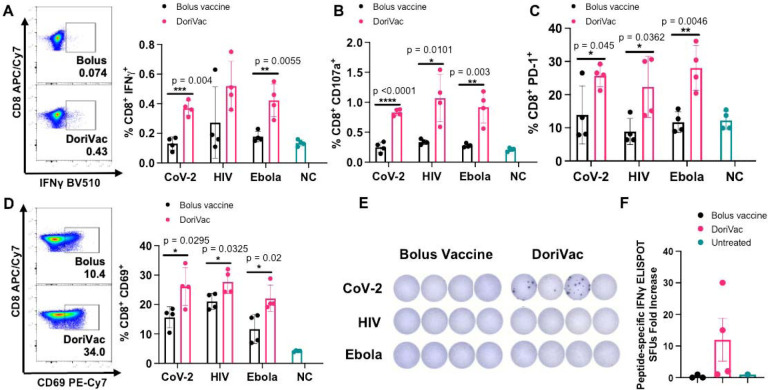
DoriVac induces enhanced antigen-specific CD8^+^ T cell activation in mice compared to bolus vaccine. **(A)** Percentages of CD8^+^ IFNγ^+^ T cells in the lymph node (LN; n=4) on Day 21 as determined by flow cytometry and representative flow plots from the SARS-CoV-2 data. NC means negative (i.e. untreated) control. **(B)** Percentages of CD8^+^ CD107a^+^ T cells in the LN (n=4) on Day 21 as determined by flow cytometry. **(C)** Percentages of CD8^+^ PD-1^+^ T cells in the LN (n=4) on Day 21 as determined by flow cytometry. **(D)** Percentages of CD8^+^ CD69^+^ T cells in the LN (n=4) on Day 21 as determined by flow cytometry and representative flow plots. **(E–F)** IFNγ ELISpot demonstrating frequency of CD8^+^ enriched antigen-specific splenocytes (n=4, day 35) and accompanying quantification of IFNγ ELISpot SFUs demonstrates an increase significant difference in SARS-COV-2 antigen-specific CD8^+^ T cell frequency after treatment with DoriVac compared with the bolus vaccine. Data are represented as mean ± SD. The flow data were analyzed by multiple unpaired t-tests and significance was defined as a two-tailed p value less than 0.05. The ELISpot data were analyzed by one-way ANOVA (with correction for multiple comparisons using Tukey’s test). Statistical significance was defined as a multiplicity-adjusted p value less than 0.05. ‘*’ refers to p ≤ 0.05; ‘**’ refers to p ≤ 0.01; ‘***’ refers to p≤ 0.001; ‘****’ refers to p ≤ 0.0001.

Furthermore, after two vaccine doses, we confirmed activation of cytotoxic CD8^+^ T cells. DoriVac increased the population of IFNγ secreting cytotoxic CD8^+^ T cells (Fig. 5A) and degranulating CD107a^+^ CD8^+^ T cells (Fig. 5B) in the LNs. PD-1 and CD69 were upregulated in the CD8^+^ T cell population (Fig. 5C–D), indicating increased activation. On Day 35, after the second dose, we quantified antigen-specific CD8^+^ enriched T cells (CD4^+^ T cell depleted by positive sorting) in the spleen via IFNγ ELISpot, showing increased antigen-specific CD8^+^ T cells after SARS-CoV-2 DoriVac (Fig. 5E, F). Co-delivery of SQB, free HIV-HR2 peptide, and free CpG did not induce similar level of T cell activation as observed with the delivery of DoriVac (Supplementary Figure 10).

## Human immune-cell validation of peptide-conjugated DoriVac

**Figure 6.**
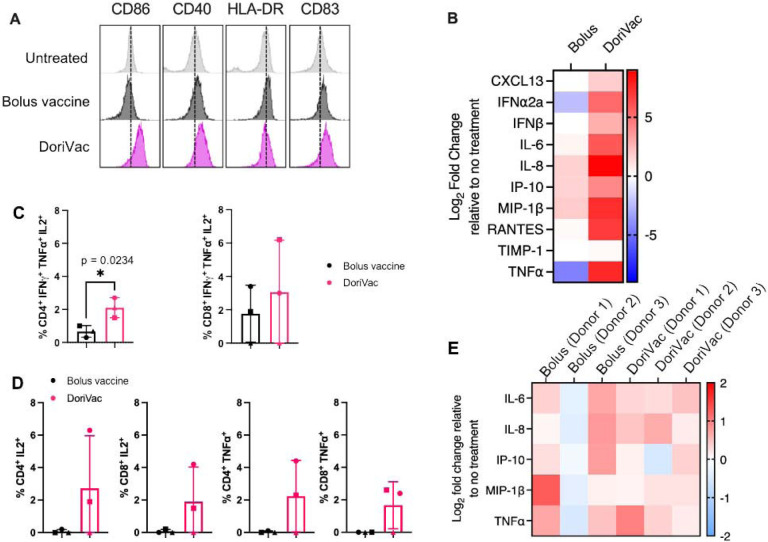
Peptide-conjugated (SARS-CoV-2-HR2) DoriVac effectively stimulates human dendritic cells (DCs) and induce enhanced immunogenicity compared to bolus vaccine on lymph node (LN) organ-on-a-chip model. **(A)** Human monocyte-derived DCs were stimulated for 24 hours with bolus or DoriVac, and the DC activation markers were analyzed using flow cytometry. **(B)** Relative fold changes in cytokines and chemokines after 24 hours of human monocyte-derived DCs with bolus or DoriVac. Fold change relative to no treatment is shown. (**C)** Graph quantifying polyfunctional T cells, as determined by their ability to co-secrete IFNγ, TNFα and IL-2 as determined via intracellular cytokine staining and flow cytometry. **(D)** LN organ-on-a-chips (n = 3) were vaccinated with bolus or the SARS-CoV-2-HR2 DoriVac. Nine days after vaccination, T cell responses were assessed via intracellular cytokine staining and flow cytometry after *ex vivo* stimulation with SARS-CoV-2 HR2 peptide and PMA/Ionomycin. Graph quantifying the average cytokine-producing CD8^+^ and CD4^+^ T cell populations at 9 days after vaccination in three different donors. Each symbol represents one donor. **(E)** Relative fold changes in IL-6, IL-8, IP-10, MIP-1β and TNFα at 9 days after transduction either of the bolus or DoriVac on the LN organ-on-a-chip. Fold change relative to no treatment is shown. Data are represented as mean ± SD. The flow data were analyzed by unpaired t-test and significance was defined as a two-tailed p value less than 0.05. ‘*’ refers to p ≤ 0.05.

Beyond murine models, we assessed DoriVac immunogenicity using a human LN organ-on-a-chip model. This model mimics the human LN for rapid prediction of vaccine responses in humans^60^. Analyzing the impact of SARS-CoV-2-HR2 DoriVac on human monocyte-derived DCs, we observed increased CD86, CD40, HLA-DR, and CD83 expression, indicating DoriVac can activate human DCs (Fig. 6A). DoriVac treatment also elevated inflammatory cytokines secreted by DCs compared to the bolus (Fig. 6B). Analyzing effector T cell responses on the human LN organ-on-a-chip model after nine days of vaccination, DoriVac displayed a substantial increase in CD4^+^ and CD8^+^ T cell activation in two of the three donors, as evidenced by TNFα^+^ and IL-2^+^ staining (Fig. 6C) Polyfunctionality analysis (examining T cells that express IFNγ, TNFα and IL-2) revealed significantly more CD4^+^ polyfunctional cells induced by DoriVac than the bolus vaccine, and an overall increase CD8^+^ polyfunctional cells (Fig. 6D). Inflammatory cytokine analysis indicated similar levels induced by bolus and DoriVac across three donors (Fig. 6E). These findings suggest that DoriVac induces a robust immune response in an *in vitro* human immune system that closely predicts human vaccine response.

## Human immune-cell validation of protein-conjugated DoriVac

We further expanded our investigation to showcase the versatility of DoriVac, enabling the conjugation of full-length viral protein antigens. Protein vaccines historically faced limitations in presenting antigens on MHC-I and inducing CD8^+^ T cell responses^61^. As a proof-of-concept, we selected hepatitis B surface antigen (HBsAg) and three monkeypox antigens (E8L, H3L and L1R), validated for prior immunogenicity in their respective diseases^62,63^. This study demonstrates DoriVac versatility in eliciting both CD4^+^ and CD8^+^ T cell responses against protein antigens.

**Figure 7.**
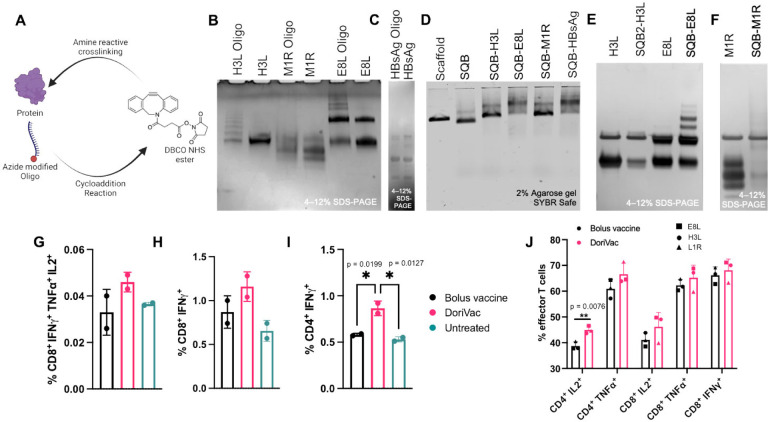
Preclinical validation of protein antigen-conjugated DoriVac immunogenicity in a human lymph node organ-on-chip model and a tonsil organoid model. **(A)** Schematic representation of protein-oligonucleotide conjugation, demonstrating the utilization of DBCO-NHS ester crosslinker to link an azide-modified oligonucleotide to the protein via free amine groups on lysines. **(B)** SDS-PAGE gel demonstrating the successful conjugation of monkeypox-specific proteins to oligonucleotides. **(C)** SDS-PAGE gel demonstrating the successful conjugation of Hepatitis B surface antigen (HBsAg) proteins to oligonucleotides. **(D)** Agarose gel demonstrating the successful conjugation of infectious-disease protein onto the SQB DNA origami. **(E–F)** Confirmation via SDS-PAGE gel of successful protein conjugation after DNase degradation of the DNA origami scaffold and staple strands and analysis of the remaining protein via silver stain. **(G–I)** LN chips (n = 2) were vaccinated with bolus or DoriVac harboring CpG and full-length HBsAg. The T cell responses were assessed using intracellular cytokine staining and flow cytometry after *ex vivo* stimulation with autologous DC-pulsed HBsAg (1:10 effector:target ratio), nine days after vaccination. Graph quantifies the **(G)** CD8^+^ polyfunctionality after nine days of vaccination in two different donors. Polyfunctionality is defined by T cells producing IFNγ, TNFα, and IL-2, **(H)** IFNγ^+^ CD8^+^ populations, and **(I)** IFNγ^+^ CD4^+^ populations. **(J)** Tonsil organoids from one donor were vaccinated with bolus or DoriVac harboring CpG and full length monkeypox antigens (E8L, H3L or L1R). T cell responses were assessed using intracellular cytokine staining and flow cytometry after *ex vivo* stimulation with 15-mer monkeypox antigenic peptides (overlapping by 11-mer) and PMA/Ionomycin stimulation for the last 4 hours of incubation. Graph quantifies the cytokine-producing effector CD8^+^ and CD4^+^ populations, nine days after vaccination (n = 3 cell samples obtained from one preparation, treated with DoriVac fabricated with three different monkeypox antigens). Data are represented as mean ± SD. The flow data were analyzed by one-way ANOVA (with correction for multiple comparisons using Tukey’s test). Significance was defined as a multiplicity-adjusted p value less than 0.05. The grouped flow data was analyzed by multiple unpaired t-tests. Significance was defined as a p value less than 0.05. ‘*’ refers to p≤ 0.05.

The protein-’anti-handle’-oligonucleotide conjugate was synthesized using DBCO-azide click chemistry, where azide-modified oligonucleotide was conjugated to a protein via a DBCO-NHS ester linker (Fig. 7A). Successful conjugation was confirmed by SDS-PAGE gel (Fig. 7B, C). The protein-’anti-handle’-oligonucleotide conjugate was hybridized to the corresponding ‘handle’ strands on the SQB via Watson-Crick base-pairing. Protein-conjugated DoriVac exhibited reduced mobility in agarose gel electrophoresis compared to unconjugated DNA origami (Fig. 7D). Successful protein conjugation was observed via SDS-PAGE gel analysis after DNase I digestion (relative to a protein-only control) (Fig. 7E,F).

Initially, we assessed T cell responses to HbsAg-conjugated DoriVac and bolus vaccine in the human LN organ-on-a-chip system nine days post-vaccination. DoriVac-stimulated DCs, pulsed with HBsAg, induced IFN-γ secretion in both CD4^+^ and CD8^+^ subsets, demonstrating antigen-specific T cell activation (Fig. 7G–I). Polyfunctionality of T cells, measured by CD8^+^ T cells secreting IFNγ, TNFα and IL-2, was higher for DoriVac than the bolus (Fig. 7I). Additionally, we evaluated the immunogenicity of monkeypox antigens using a tonsil organoid model^64^, observing increased CD4^+^ IL-2^+^ and TNFα^+^ effector T cells, as well as a higher percentage of IL-2^+^, TNFα^+^ and IFNγ^+^ CD8^+^ T cells (Fig. 7J). These findings illustrate the capability of DoriVac to induce cellular immunity against protein antigens of various infectious diseases.

## Conclusions

Enduring immune memory and protection against viral variants rely on cellular immune responses, particularly CD8 T cells that target less mutable viral proteins. SARS-CoV-2 vaccines with greater than 90% protection demonstrated induction of Th1-skewed immunity^65–70^, emphasizing the importance of Th1 CD4 and CD8 T cell responses. In recent decades, DNA origami has achieved crucial milestones, indicating its potential as a therapeutic platform. Its programmability makes it a versatile ‘plug-and-play’ vaccine platform, particularly relevant for emerging infectious diseases. In this proof-of-concept study, HR2-DoriVac, conjugated with infectious-disease-associated peptides and proteins, successfully elicited robust neutralizing antibodies and antigen-specific CD4 and CD8 T cell activation in healthy mice, a notable contrast to some SARS-CoV-2 vaccines with limited T cell responses. DoriVac presents four distinct advantages: (1) precise nanoscale arrangement of antigen and adjuvant for their co-delivery on each nanoparticle; (2) well-established, simple, and scalable fabrication; (3) modular, programmable design adaptable for various antigens via DNA hybridization; (4) stability at 4°C, contrasting with mRNA vaccines needing −20°C to −80°C cold chain storage^48^.

While robust antigen-specific T cell activation was observed with SARS-CoV-2-HR2 DoriVac, the same level of activation was not observed for HIV and Ebola. However, significant activation of B cells, DCs, CD4 and CD8 T cells for HIV and Ebola, suggested a strong immune response, albeit possibly insufficient for protection from infection. The chosen antigens for HIV and Ebola are also predicted to be weakly immunogenic in humans (Supplementary Table 11–14), so future studies could focus on identifying more immunogenic peptides for HIV and Ebola for human HLA, potentially enhancing antigen-specific activation. Viral rechallenge studies, contingent on availability of BL3 facilities, could further assess the effectiveness of DoriVac-induced immune activation against live viruses. Additionally, a direct comparison with mRNA-based vaccines would be beneficial; however, differences in dosing regimens and experimental setups may present challenges in making direct comparisons.

This proof-of-concept study suggests the potential of DoriVac, when conjugated with antigens associated with emerging infectious diseases, to rapidly manufacture vaccines capable of offering protection against variants and future outbreaks. The unique programmable modularity of DoriVac allows for the creation of multiplexed nanoparticles, each carrying a different antigen. This feature holds the promise of developing a universal vaccine, adaptable to a wide range of pathogens, by targeting multiple antigens simultaneously. Such versatility and adaptability establish DoriVac as a significant advancement in the field of vaccine technology, especially in the context of rapidly evolving infectious threats.

## Materials and Methods

### SQB fabrication

SQB fabrication is detailed in a previous publication, including scaffold and staple sequences^55,71^. Scaffold p8634 was produced in-house, as previously published^72^. DNA staple strands were purchased from IDT. Folding concentrations were 5 mM Tris base, 1 mM ethylenediaminetetraacetic acid (EDTA; pH 8.0), 12 mM MgCl_2,_ 20–100 nM scaffold, 5 times excess of the basic staple strands (relative to scaffold concentration), 10 times excess of handle-conjugated staple strands (for attachment of relevant infectious-disease antigens) and 20 times excess CpG-staple strands. An 18-hour thermocycler program was used to fabricate SQBs: denaturation at 80°C for 15 minutes, then annealing via a temperature ramp from 50°C to 40°C decreasing at −0.1°C every 10 minutes and 48 seconds. Most staple strands include ten thymidine residues at the end of the double helices to minimize aggregation. The CpG-containing strands were appended on the flat face of the SQB. The CpG oligonucleotides with nuclease-resistant phosphorothioate backbones (5´ – TCCATGACGTTCCTGACGTT-3´, IDT) replaced the corresponding thymine residues in a 3.5 nm nanoscale pattern as determined previously^55^. CpG was appended to the 5´ end of designated strands.

### HR2 peptide conjugation with ‘anti-handle’ oligonucleotide

An ‘anti-handle’ oligonucleotide, which corresponds to 24 sites of ‘handle’ oligonucleotide on the extruding face of the SQB, was ordered from IDT with an 5’ amine (aminoC6-TTCTAGGGTTAAAAGGGGACG). HR2 peptides were ordered from GenScript with an azide-modified N-terminal lysine (Fig. 1 I–L; Supplementary Table 2) for DBCO-Azide copper-free click chemistry reaction between the peptide and oligonucleotide. The oligonucleotide was prepared at 1 mM with phosphate buffer (pH 8.0). The dibenzocyclooctyne-N-hydroxysuccinimidyl ester (DBCO-NHS ester; Millipore, #761524) (diluted in DMSO to 2 mM) was incubated with the oligonucleotide (diluted in phosphate buffer pH 8.0 to 100 uM) in 1:1 volume ratio and 1: 20 oligonucleotide to DBCO ratio (with final concentration of DBCO >1 mM) and incubated overnight at ambient temperature. The oligonucleotide-DBCO was purified via Illustra NAP column (GE Healthcare Life Sciences, #17–0852-02), eluted with sterile water, and concentrated via 3K Amicon Ultra Centrifugal Filter Unit (Millipore; #UFC500324). DBCO incorporation was confirmed via OD310 peak. Concentration measured via A260 with the NanoDrop. The Azide-modified HR2 peptides, representing SARS-CoV-2, HIV and Ebola, were dissolved in DMSO to 5 mM. The peptide-Azide was mixed with the oligonucleotide-DBCO at a ratio of 1.5: 1 and incubated overnight at room temperature in 1 × PBS.

### Denaturing PAGE (dPAGE) verification and purification of peptide-conjugated oligonucleotide

15% denaturing PAGE (dPAGE) was used to confirm peptide-oligonucleotide conjugation. dPAGE gel (15%) was made in-house using 9 mL urea concentrate (Fisher Scientific #EC-833), 4.5 mL urea dilutant, 1.5 mL urea buffer, 10 μL tetramethylethylenediamine (TEMED) and 150 μL 10 wt% ammonium persulfate, and cast into a cassette (ThermoFisher Novex #NC2010)^55^. 5 picomoles of sample were mixed with formamide loading buffer (FLB) in 1:1 volume ratio. The mixture was denatured at 80°C for 10 minutes and loaded into the wells. The gel was run in 0.5 × TBE buffer for 45 minutes at 250V, stained with SYBR Gold for 10 minutes, and imaged with the Typhoon Gel Scanner. For purification, the mixture was combined with formamide loading buffer (FLB) at a volume ratio of 1:1, loaded into an 8% dPAGE gel in a large well-formed using a taped comb, and run at 250V for 50 minutes. The peptide-conjugated oligonucleotide was observed via UV shadowing on a thin layer chromatography plate, and cut out with a razor blade. The gel was crushed thoroughly with a pestle and immersed in 1 × TE buffer. The solution was incubated while shaking overnight at 25°C, filtered through Freeze ‘N Squeeze DNA Gel Extraction Spin Columns (Bio-Rad; #7326165) and purified via ethanol precipitation. The peptide-oligonucleotide was resuspended in 1 × PBS; the concentration was determined via NanoDrop. Purification was confirmed via dPAGE.

### HR2 peptide-conjugated oligonucleotide hybridization with SQB

The purified peptide-oligonucleotides were hybridized to the SQB DNA origami in 2 × excess, maintaining 10 mM MgCl_2_ and 1 × TE via addition of stock 10 × TE and 100 mM MgCl_2_. The SQBs were added last so they were not subjected to variable buffer. The peptide-SQBs were incubated for 1–2 hours at 37°C while shaking, then purified through PEG precipitation and analyzed via agarose gel electrophoresis, TEM, and DNase I degradation assay.

### Agarose gel electrophoresis

SQBs were analyzed via 2% native agarose gel electrophoresis. Gel was prepared with 0.5 × TBE buffer with 11 mM MgCl_2_ and 0.005% v/v SYBR Safe (ThermoFisher #S33102), run at 70V for 2 hours, and imaged via a Typhoon Gel Scanner.

### Transmission electron microscopy (TEM) analysis

Structural integrity and SQB aggregation were verified using negative-stain TEM. Formarcoated, carbon-stabilized grids were prepared in-house or purchased (Electron Microscopy Services, FCF200-CU-TA). Grids were passivated using plasma discharge for 30 seconds. Four μl of 4–10 nM SQBs was deposited on a grid. After 45 seconds, the sample was wicked away by blotting with filter paper. A drop of uranyl-formate solution (0.75% w/v in H_2_O) was deposited onto the grid and blotted off. A second drop of 0.75% uranyl formate solution was deposited onto the grid for 2 minutes and then blotted off. Grids were imaged with a JEOL JEM-1400 TEM in brightfield mode at 120 kV.

### SQB purification via PEG precipitation

CpG-SQBs or peptide-conjugated CpG-SQBs were purified via PEG precipitation. 1 × TE buffer (5 mM Tris base, pH 8.0 and 1 mM EDTA acid) containing 15% w/v PEG-8000 (Fisher Scientific, BP2331) and 510 mM NaCl was added to the SQB sample at 1:1 volume and mixed gently via pipetting. MgCl_2_ stock was added to the PEG solution to achieve 10 mM MgCl_2_ final concentration. As described previously, the solution was incubated for 30 min on the benchtop, centrifuged at 16000 g for 25 minutes and the supernatant was removed^55^. This procedure purifies and concentrates the sample, as a high concentration is often required for further studies. The concentration was determined via Nanodrop; the sample purity and integrity were confirmed via agarose gel electrophoresis and TEM.

### DNase I degradation and silver stain analysis of peptide conjugation efficiency

One μg of SQBs was incubated with 1.0 U/μL DNase I (NEB) with 10 × DNase I buffer diluted in water (New England Biolabs #M0303S). Samples were incubated in the thermocycler for 30 minutes at 37°C. The sample was mixed with 4 × NuPAGE LDS sample buffer (ThermoFisher; #NP0008), incubated at 95°C for 2 minutes, and loaded in 4–12% NuPAGE Bis-Tris gels (ThermoFisher; #NP0322). The gel was run in 1 × MES SDS running buffer (ThermoFisher; #NP0002) at 150V for 45 minutes. The gel was analyzed via silver stain (Pierce, #24612), following the manufacturer’s protocol. The gel was imaged under the Silver Stain setting on Image Lab 6 on a Gel Doc EZ Imager (Bio-Rad). ImageJ was used to quantify band intensities and determine peptide loading efficiencies.

### K10-PEG5k coating of SQBs

PEG-purified SQBs were mixed with oligolysine-PEG5k (K10-PEG5k; methoxy-poly(ethylene glycol)-block-poly(L-lysine hydrochloride); n=113, x=10; Alamanda Polymers (mPEG20K-b-PLKC10). The number of phosphates in the SQB was calculated based on the sequence. An appropriate amount of K10-PEG5k was added such that the number of nitrogens in the amines of K10-PEG5k was equivalent to the number of SQB phosphates, according to the previously published method^73^. Samples were incubated at 37°C for a minimum of 30 minutes. The concentration was calculated based on the dilution.

### Animal model and treatment

C57BL/6 mice (6–8 weeks) were purchased from Jackson Laboratory and maintained in the Harvard Medical School (HMS) animal facility. Eight groups were tested with eight mice per group: (1) SARS-CoV-2-HR2 DoriVac (2) SARS-CoV-2 HR2 bolus, (3) HIV-HR2 DoriVac, (4) HIV-HR2 bolus, (5) Ebola-HR2 DoriVac, (6) Ebola-HR2 bolus, (7) SQB-CpG + free HIV-HR2 peptide, and (8) untreated. The bolus consisted of an equivalent dose of CpG, and HR2 peptide in PBS. After one-week accommodation, the first treatment dose (100μL of DoriVac containing 0.36 nmoles of CpG, 0.48 nmoles of HR2 peptide) was injected subcutaneously in the upper back midline of each mouse on day 0 and day 20. Four hours after, blood was harvested via submandibular vein draw. On day 14, blood was collected via submandibular vein draw. On day 20, the second dose was administered. On day 21, four mice in each group were sacrificed; heart blood, LNs, and spleens were harvested. On day 28, blood was collected via submandibular vein draw. On day 35, the remaining mice were sacrificed; heart, blood, LNs, spleens and femurs were harvested. Studies were approved by the HMS Institutional Animal Care and Use Committee.

### Lymph-node-on-a-chip and tonsil organoid vaccination

Lymph-node-on-a-chip (LN chip) was fabricated as previously described^60^. Tonsil organoids was seeded according to a prior publication^64^. Both human patient derived apheresis collars and tonsil were obtained from the Crimson Biomaterials Collection Core Facility under approval of Harvard University’s Institutional Review Board. Chips and organoid culture were treated with 1nM of vaccine. In LN chip experiments, medium (RPMI supplemented with 10% FBS, 1% antibiotics, IL-2 and IL-4 as previously described^60^) was recirculated for 4 days of treatment (i.e., effluents were added back to the inlet perfusion reservoir), and at day 4, a 1:1 mix of effluent and fresh medium was used for perfusion to maintain the cytokine milieu. For tonsil organoid experiment, at day 4, fresh media was added 1:1 to the well. At the conclusion of the chip study, cells were harvested by blocking one port of the basal channel with a tip and manually pipetting Cell Recovery Medium (Corning, 354253, 200 μL chip−1) through the other port to harvest the ECM and cells. ECM was incubated in the Cell Recovery Medium for 1 hour at 4 °C to depolymerize the ECM and recover associated cells The released cells were centrifuged at 300 × g for 5 min and resuspended in PBS.

### Processing blood cells

Blood was collected either from the heart with heparin-rinsed syringes or via submandibular cheek draw into heparin-coated tubes. Plasma was separated from blood cells and collected vi centrifugation at 800g at 4°C for 5 min. The plasma stored directly in −80°C until analysis, and the blood cells were treated with red blood cell lysis buffer three times (RBC lysis buffer (10 ×); BioLegend; #420301), according to manufacturer’s protocol. The peripheral blood mononuclear cells (PBMCs) were analyzed via ELISpot and/or flow (Cytoflex LX).

### Luminex Multiplex ELISA analysis

The Bio-Plex Pro Mouse Cytokine Standard 23-plex kit (Bio-Rad) was customized to include the following: IL-1α , IL-1β, IL-2, IL-3, IL-4, IL-5, IL-6, IL-9, IL-10, IL-12p40, IL-12p70, IL-13, IL-17A, Eotaxin, G-CSF, GM-CSF, IFNγ, MCP-1, MIP-1α, MIP-1β, RANTES, TNFα. The manufacturer’s protocol was followed, and data was collected via Bio-Plex 3D Suspension Array System (Bio-Rad).

### Processing lymph nodes (LNs)

After euthanasia, the upper axillary and superficial cervical LNs were harvested from the mouse and stored in cold PBS as described previously.^55^ The LNs were processed into single cell suspensions for flow-cytometry analysis (Cytoflex LX). LNs were gently mashed through a 40 μm cell strainer using a sterile syringe plunger into a petri dish. Cells were collected into 1.5 mL Eppindoff tubes, pelleted at 400g for 5 min at 4°C, and the supernatant was discarded. The cell pellet was resuspended in 700 μL of PBS and separated into 96 well plates for flow cytometry analysis.

### Processing spleens

After euthanasia, mouse spleens were harvested. The spleens were washed with PBS and mashed through a 40 μm cell strainer using a sterile syringe plunger into a 60 mm petri dish. The single cell suspension was washed with complete RPMI-1640 media (containing 10% fetal bovine serum and 1% penicillin-streptomycin), collected into a Falcon tube, and treated two times with red blood cell lysis buffer (RBC lysis buffer (10X); BioLegend; #420301), according to manufacturer’s protocol. The cell pellet was resuspended in 2 mL of complete RPMI media, and the cell number was counted. Splenocytes were then applied for flow cytometry or ELISpot assays.

### Processing bone marrow

After euthanasia, femurs were collected and washed with PBS several times. Muscle fibers and connective tissues were removed with forceps. The marrow was flushed from the bone using a syringe and collected into a dish with PBS. The marrow clot was pipetted and filtered through a 40 μm cell strainer and collected into a Falcon tube. The single cell suspension was pelleted at 300 × g for five minutes. Supernatant was discarded, the pellet was resuspended, and the cells were treated with red blood cell lysis buffer (RBC lysis buffer (10X); BioLegend; #420301), according to the manufacturer’s protocol. The suspension was pelleted at 300 × g for five minutes and resuspended in culture media. B cells were analyzed via flow (Cytoflex LX).

### Flow cytometry

Single cell suspensions of LNs, PBMCs, spleens, and bone marrow were obtained. The suspensions were washed with PBS, stained with Zombie UV viability dye (BioLegend; #423108) and washed with cell staining buffer (BioLegend, #420201). The cells were stained with fluorophore-conjugated cell surface antibodies (Supplementary Table 4–6). Intracellular staining was performed, relying on permeabilization and fixation reagents (BioLegend; #424401). Antibodies were arranged into appropriate panels, compensations were set up to account for fluorescent emission overlap, and the stained cells were analyzed on a Cytoflex LX flow cytometer. Storage events were gated on the population of interest, based on protocols published previously^55^, and according to the gating shown in Supplementary Figures 2, 5, and 9. Flow data was analyzed using FlowJo V10.

### CD8 and CD4 enrichment of splenocytes

Splenocytes were depleted for CD4 or CD8 T cells using CD8 Dynabeads^™^ (Thermofisher, #11145D) or CD4 Microbeads (Miltenyi Biotec, #130–117-043), according to manufacturer’s instructions. The remaining sample was enriched for CD4 T cells (via CD8 T cell depletion) or enriched for CD8 T cells (via CD4 T cell depletion). Splenocytes were maintained in 4°C for 36 hours before processing for CD8 T cell enrichment. For CD8 depletion, the Dynabeads^™^ were washed in isolation buffer and placed in the magnet. At the same time, cells were prepared at a concentration of 1 × 10^7^ cells per mL in isolation buffer. The prewashed beads were added, and the solution was incubated for 30 minutes at 4°C with gentle tilting. After, the tubes were placed in the magnet for 2 minutes and the supernatant was transferred to a new tube for further analysis. The beads and associated cells were discarded. For CD4 depletion via Microbeads, the cells were prepared in the appropriate buffer and the microbeads were added. The solution was incubated for 10 minutes at 4°C. The cell suspension was added to the LD column and the flow through, representing the CD8 enriched (CD4 depleted) population, was collected for further analysis.

### IFNγ ELISpot

After samples were processed into single cell suspensions, PBMCs or splenocytes were plated into a 96-well round-bottom plate, with each well containing cells from an individual mouse in 200 μl of media. Two and a half million cells were used for splenocytes on day 21. Three million cells were used for the splenocytes on day 35. Two million cells were used for PBMCs. Eight and a half million cells were used for the CD4 and CD8 enriched samples. We stimulated each well with 2 μg/mL of HR2 peptide. After 48 hours of stimulated pre-incubation at 37°C, we collected the cells (290 g, 5 min, 4°C) and resuspended in 100 μl of sterile splenocyte media. The cells were then plated for analysis on ELISpot plate (RND systems, Mouse IFNγ ELISpot kit, #505841). The cells were incubated at 37°C for 36 hours to allow time for epitope processing and MHC presentation and then processed the ELISpot plate according to the manufacturer’s instructions. After the plate was processed and dried overnight, the plate was analyzed via an ELISpot plate reader by Dana Farber Cancer Institute’s Center for Immuno-oncology Translational Immunogenics Laboratory. The fold increase in cells for DoriVac treated groups versus bolus groups was quantified.

### Enzyme-linked Immunosorbent Assay (ELISA)

IgG in plasma from vaccinated mice was quantified by enzyme-linked immunosorbent assay (ELISA). Nunc Maxisorp ELISA plates (Thermo Fisher Scientific Inc., USA #44–2404-21) were coated with HR2 peptide (SARS-CoV-2, HIV or Ebola) at a concentration of 2–20 μg/mL in 100 μL of coating buffer (100 mM bicarbonate/carbonate buffer, pH 9.5) and incubated overnight at 4 . After washing three times with washing buffer (PBS containing 0.05% Tween 20), 150 μL of blocking buffer (2% bovine serum albumin (Sigma, USA #9048–46-8) in washing buffer) was applied for 1 h at 37 . After removing the blocking buffer, 100 μL of plasma samples diluted in blocking buffer (1:100, 1:200, 1:400 dilutions) were added and incubated for 1 h at 37 . After washing three times with washing buffer, 150 μL of blocking buffer was applied for 1 h at 37 . After removing the blocking buffer, 100 μL of HRP-conjugated anti-mouse IgG antibody (Cell Signaling Technology, USA **#**7076) diluted in blocking buffer was applied for 1 h at 37 . After washing five times with washing buffer, 50 μL of 3,3’,5,5’-tetramethyl benzidine (TMB) substrate (Sigma #54827–17-7) for detection was added, and the reaction was stopped after 15 min by the addition of 50 μL of 1 M H_2_SO_4_. The spectroscopic absorbance was determined by an automated plate reader (BioTek, USA) at a wavelength of 450 nm.

### Pseudovirus assay

Plasma was isolated by collecting the clear supernatant after centrifugation. Samples were diluted in 1 × PBS at various ratios (1:10, 1:100, 1:1000) and cultured with the appropriate pseudovirus SARS-CoV-2pp, HIV-pp, Ebola-pp) and ACE2–293 T cells. Data in the figures is at the optimal 1:100 dilution. The relative pseudovirus infection level was quantified as the ratio of number of cells infected in each group compared to the number of cells infected in the bolus group. Therefore, bolus groups had a relative infection of 1.0.

### Statistical analyses

One-way or two-way ANOVA or unpaired t-test(s) with appropriate corrections for multiple comparisons was applied to determine the statistical significance of all flow, ELISpot and ELISA data in Figures 2–7. GraphPad Prism 10 was used to make graphs and calculate p values. A p value ≤ 0.05 was considered statistically significant. ‘*’ refers to p ≤ 0.05; ‘**’ refers to p ≤ 0.01; ‘***’ refers to p≤ 0.001; ‘****’ refers to p ≤ 0.0001. Error bars represent standard deviation (SD).

## Supplementary Material

Supplement 1

## Data Availability

The paper and supplementary materials contain the supporting data for this study.
