# Supplementary material for: DNA origami vaccine (DoriVac) nanoparticles improve both humoral and cellular immune responses to infectious diseases
Source: bioRxiv. 2025 Apr 4:2023.12.29.573647. Originally published 2024 Jan 2. Preprint. [Version 2] doi: 10.1101/2023.12.29.573647 (PMC10802255; doi:10.1101/2023.12.29.573647)
Supplement: Supplement 1 [file media-1.pdf]

Supplementary Information for:

DNA origami vaccine (DoriVac) nanoparticles improve both humoral and cellular immune responses to infectious diseases

Yang C. Zeng[1,2,3*#], Olivia J. Young[2,3,4*], Qiancheng Xiong[1,2,3*], Longlong Si[2*], Min Wen Ku[2*], Sylvie G. Bernier[2], Hawa Dembele[1,2,3,5], Giorgia Isinelli[1,2], Tal Gilboa[2,6], Zoe Newell Swank[2,6], Su Hyun Seok[1,2,3,7], Anjali Rajwar[2], Amanda Jiang[2], Yunhao Zhai[2], LaTonya D. Williams[8], Caleb A. Hellman[8], Chris M. Wintersinger[1,3], Amanda R. Graveline[2], Andyna Vernet[2], Melinda Sanchez[2], Sarai Bardales[2], Georgia D. Tomaras[8], Ju Hee Ryu[1,7,9], Ick Chan Kwon[1,7,9], Girija Goyal[2#], Donald E. Ingber[2,10,11#], William M. Shih[1,2,3#].

* These authors contributed equally to this work
# Correspondence to: william_shih@dfci.harvard.edu; don.ingber@wyss.harvard.edu; girija.goyal@wyss.harvard.edu; yang_zeng@dfci.harvard.edu

[1]Department of Cancer Biology, Dana-Farber Cancer Institute, Harvard Medical School, Boston, Massachusetts 02115, USA
[2]Wyss Institute for Biologically Inspired Engineering, Harvard University, Boston, Massachusetts 02215, USA
[3]Department of Biological Chemistry and Molecular Pharmacology, Harvard Medical School, Boston, Massachusetts 02115, USA
[4]Harvard-Massachusetts Institute of Technology (MIT) Division of Health Sciences and Technology, Massachusetts Institute of Technology, Cambridge, Massachusetts 02139, USA
[5]Department of Systems Biology, Harvard Medical School, Boston, Massachusetts 02115, USA
[6]Department of Pathology, Brigham and Women's Hospital, Harvard Medical School, Boston, Massachusetts 02115, USA
[7]Medicinal Materials Research Center, Biomedical Research Institute, Korea Institute of Science and Technology (KIST), Seoul 02792, Republic of Korea
[8]Center for Human Systems Immunology, Departments of Surgery and Integrative Immunobiology, Duke University School of Medicine, Durham, North Carolina 27701, USA
[9]KU-KIST Graduate School of Converging Science and Technology, Korea University, Seoul 02841, Republic of Korea
[10]Vascular Biology Program, Department of Surgery, Boston Children's Hospital and Harvard Medical School, Boston, Massachusetts 02115, USA
[11]Harvard John A. Paulson School of Engineering and Applied Sciences, Boston, Massachusetts 02134, USA

Variant	Sequence
SARS-CoV-2 (Alpha)	GDISGINASVVNIQKEIDRLNEVAKNLNESLIDLQELG KYEQYIK
SARS-CoV-2 (Omicron)	GDISGINASVVNIQKEIDRLNEVAKNLNESLIDLQELG KYEQGSG

Supplementary Table 1 | Differences between HR2 sequences in variants of SARS-CoV-2. The amino acids that differ between the two variant sequences are colored in red. HR2 peptide is highly conserved, compared to many epitopes within the spike region, which renders the effectiveness of HR2-based SARS-CoV-2 vaccines less susceptible to antigenic drift.

Peptide Name	Sequence	Molecular Weight	Number of Residues	Net Charge at pH 7	Hydrophobicity (GRAVY)
SARS-CoV-2 HR2	[Lys(N3)]GDIS GINASVVNIQK EIDRLNEVAKN LNESLIDLQEL GKYEQYIK	5203.81 g/mol	46	-2.0	-0.52
HIV HR2	[Lys(N3)]WNN MTWMEWDRE INNYTSLIHSLI EESQNQQEKN EQELLELDKW ASLWNWF	6600.19 g/mol	52	-5.9	-1.14
Ebola HR2	[Lys(N3)]IEPHD WTKNITDKIDQ IIHDFVDK	3049.38 g/mol	25	-1.8	-0.98

Supplementary Table 2 | Peptide sequences and relevant properties. Peptides were purchased with N-terminal azide modifications from GenScript. The azide modifications enable a click-chemistry reaction between the azide-modified peptide and a DBCO-modified oligonucleotide. A negative value in the Grand AVerage of hydropathY (GRAVY) score indicates peptides that are net hydrophilic at pH 7.

Sample	Intensity of DoriVac Band	Intensity of Peptide Band (Control)	Percent Conjugation Efficiency
SARS-CoV-2 DoriVac	5885.468	5770.296	102.0%
Ebola DoriVac	2660.154	2573.125	103.4%
HIV DoriVac	3330.874	3436.288	96.9%

Supplementary Table 3 | Peptide conjugation efficiency. Peptide conjugation efficiency was determined by using the band intensity of a DNase I-digested infectious disease SQB, as calculated by ImageJ, and comparing it with the band intensity of the theoretical amount of peptide.

Antibody	Fluorophore	BioLegend Catalog Number
CD11c	BV510	117338
CD40	PE	124610
CD80	APC/fire 750	104740
CD86	PE/Cy7	105014
MHC-II	BV421	107632
PD-L1	BV785	124331
DEC205	PerCp/Cy 5.5	138207
CD11b	BV605	101257
CD103	AF700	121442
Gr-1	FITC	108406
Viability	Zombie UV	423108

Supplementary Table 4 | Antibodies and fluorophores used for flow cytometric characterization of dendritic cell (DC) phenotypes in our DoriVac studies. This table lists the antibodies employed to delineate DC lineage, activation status, and antigen-presentation capacity. Markers include DC lineage proteins (CD11c, CD11b, CD103, Gr-1), co-stimulatory molecules (CD40, CD80, CD86), antigen presentation molecules (MHC-II, DEC205), and the immune checkpoint regulator PD-L1, alongside the Zombie UV viability dye. These antibodies were assembled into panels according to specific experimental objectives to evaluate the DC responses associated with DoriVac immunogenicity.

Antibody	Fluorophore	BioLegend Catalog Number
B220	BV510	103247
CD19	AF700	115528
CD138	BV650	142518
CD38	PE/Fire 700	102747
IgG1	PerCP/Cyanine5.5	406612
IgG2a	BV421	407117
CD40	PE/CY7	124622
CD27	APC/fire750	124237
PD-L2	PE594	107216
Viability	Zombie UV	423108

Supplementary Table 5 | Antibodies and fluorophores used for flow cytometric analysis of B cell subsets in our DoriVac studies. This table details the antibodies used to characterize B cell populations and monitor their activation and differentiation following DoriVac immunization. Markers such as B220 and CD19 identify the overall B cell compartment, while CD138 marks plasma cells. CD38 and CD27 help assess activation and memory status, whereas IgG1 and IgG2a detect class-switched immunoglobulins. Additionally, CD40 and PD-L2 indicate co-stimulatory functions. The Zombie UV viability dye is used to exclude dead cells. Antibodies were organized into panels tailored to specific experimental objectives, with each entry listing the corresponding BioLegend catalog number.

Antibody	Fluorophore	BioLegend Catalog Number
CD3	BV785	100232
CD8	APC/Cy7	100713
CD4	PE	100408
CD69	PE-Cy7	104512
CD62L	PerCp	104430
CD44	AF-700	103026
CD127	APC	135012
CD25	BV650	102037
PD-1	BV605	135220
CD107a	FITC-AF488	121608
Viability	Zombie UV	423108
FoxP3 (Intra)	BV421	126419
TNFα (Intra)	PE594	506346
IL-2 (Intra)	PE - Cy5	503824
IFN-γ (Intra)	BV510	505841

Supplementary Table 6 | Antibodies and fluorophores for multiparametric T cell profiling in DoriVac studies. This table lists the antibodies used to characterize T cell subsets and assess activation, differentiation, and effector functions following DoriVac immunization. Markers such as CD3, CD4, and CD8 define the T cell compartment, while CD69, CD62L, and CD44 provide insight into activation and memory status. CD127, CD25, and PD-1 further refine the phenotypic profile, and CD107a indicates degranulation. Intracellular staining for FoxP3 identifies regulatory T cells, and TNFα, IL-2, and IFN-γ are measured to evaluate cytokine production. The Zombie UV viability dye is used to exclude dead cells. Antibodies were organized into specific panels according to experimental goals, and each entry includes the corresponding BioLegend catalog number.

MHC-I or MHC-II	Allele	Start	End	Length	Peptide	Core peptide region	Rank
MHC-I	H-2-Db	8	17	10	ASVVNIQKEI	ASVVNIQEI	0.04
MHC-I	H-2-Db	3	11	9	ISGINASVV	ISGINASVV	0.09
MHC-I	H-2-Kb	6	13	8	INASVVNI	INAS-VVNI	0.37
MHC-I	H-2-Kb	30	37	8	SLIDLQEL	SLID-LQEL	0.37
MHC-I	H-2-Db	8	20	13	ASVVNIQKEIDRL	ASVVNIDRL	0.39
MHC-I	H-2-Db	29	37	9	ESLIDLQEL	ESLIDLQEL	0.44
MHC-I	H-2-Kb	13	20	8	IQKEIDRL	IQKE-IDRL	0.99
MHC-I	H-2-Db	4	11	8	SGINASVV	SGI-NASVV	0.68
MHC-II	H2-IAb	2	16	15	DISGINASVVNIQKE	INASVVNIQ	2
MHC-II	H2-IAb	3	17	15	ISGINASVVNIQKEI	INASVVNIQ	2.4
MHC-II	H2-IAb	1	15	15	GDISGINASVVNIQK	INASVVNIQ	3.4
MHC-II	H2-IAb	4	18	15	SGINASVVNIQKEID	INASVVNIQ	4.4
MHC-II	H2-IAb	5	19	15	GINASVVNIQKEIDR	INASVVNIQ	18
MHC-II	H2-IAb	15	29	15	KEIDRLNEVAKNLNE	DRLNEVAKN	29
MHC-II	H2-IAb	14	28	15	QKEIDRLNEVAKNLN	DRLNEVAKN	36
MHC-II	H2-IAb	16	30	15	EIDRLNEVAKNLNES	DRLNEVAKN	40

Supplementary Table 7 | Predicted murine MHC epitopes of the SARS-CoV-2 HR2 peptide and associated MHC binding. NetMHCpan-4.1 was used to predict the top eight murine MHC-I and MHC-II epitopes of each HR2 peptide. The default cutoff for MHC binders was used as described by NetMHCpan[59]. A lower rank score for the peptide is associated with stronger binding to MHC. 0.5% was defined as the cutoff for 'strong' MHC-I binders while 2% was defined as the cutoff for 'weak' MHC-I binders. Any peptides that had a rank score >2% were assumed to not bind to MHC-I. 2% was defined as the cutoff for 'strong' MHC-II binders while 10% was defined as the cutoff for 'weak' MHC-II binders. Any peptides that had a rank score >10% were assumed to not bind to MHC-II.

MHC-I or MHC-II	Allele	Start	End	Length	Peptide	Core peptide region	Rank
MHC-I	H-2-Db	11	19	9	REINNYTSL	REINNYTSL	0.09
MHC-I	H-2-Kb	17	24	8	TSLIHSLI	TSL-IHSLI	0.42
MHC-I	H-2-Kb	16	23	8	YTSLIHSL	YTS-LIHSL	0.5
MHC-I	H-2-Db	30	38	9	QQEKNEQEL	QQEKNEQEL	0.39
MHC-I	H-2-Db	27	38	12	SQNQQEKNEQEL	SQNQNEQEL	0.43
MHC-I	H-2-Kb	12	19	8	EINNYTSL	EINN-YTSL	0.79
MHC-I	H-2-Db	11	20	10	REINNYTSLI	REINNYTSI	0.7
MHC-I	H-2-Db	29	38	10	NQQEKNEQEL	NQQENEQEL	0.85
MHC-II	H2-IAb	16	30	15	YTSLIHSLIEESQNQ	LIHSLIEES	11
MHC-II	H2-IAb	15	29	15	NYTSLIHSLIEESQN	LIHSLIEES	14
MHC-II	H2-IAb	14	28	15	NNYTSLIHSLIEESQ	LIHSLIEES	20
MHC-II	H2-IAb	17	31	15	TSLIHSLIEESQNQQ	LIHSLIEES	20
MHC-II	H2-IAb	12	26	15	EINNYTSLIHSLIEE	NYTSLIHSL	21
MHC-II	H2-IAb	13	27	15	INNYTSLIHSLIEES	YTSLIHSLI	23
MHC-II	H2-IAb	11	25	15	REINNYTSLIHSLIE	NYTSLIHSL	26
MHC-II	H2-IAb	21	35	15	HSLIEESQNQQEKNE	IEESQNQQE	30

Supplementary Table 8 | Predicted murine MHC epitopes of the HIV HR2 peptide and associated MHC binding. NetMHCpan-4.1 was used to predict the top eight murine MHC-I and MHC-II epitopes of each HR2 peptide. The default cut-off for MHC binders was used as described by NetMHCpan[59]. A lower rank score for the peptide is associated with stronger binding to MHC. 0.5% was defined as the cut-off for 'strong' MHC-I binders while 2% was defined as the cut-off for 'weak' MHC-I binders. Any peptides that had a rank score >2% were assumed to not bind to MHC-I. 2% was defined as the cut-off for 'strong' MHC-II binders while 10% was defined as the cut-off for 'weak' MHC-II binders. Any peptides that had a rank score >10% were assumed to not bind to MHC-II.

MHC-I or MHC-II	Allele	Start	End	Lengt h	Peptide	Core peptide region	Rank
MHC-I	H-2-Db	8	17	10	KNITDKIDQI	KNITDIDQI	1.8
MHC-I	H-2-Kb	13	21	9	KIDQIIHDF	KIDQIIHDF	4
MHC-I	H-2-Kb	10	17	8	ITDKIDQI	IT-DKIDQI	4.9
MHC-I	H-2-Db	10	18	9	ITDKIDQII	ITDKIDQII	4
MHC-I	H-2-Db	8	18	11	KNITDKIDQII	KNITIDQII	5.3
MHC-I	H-2-Db	6	14	9	WTKNITDKI	WTKNITDKI	5.6
MHC-I	H-2-Db	13	21	9	KIDQIIHDF	KIDQIIHDF	6.1
MHC-I	H-2-Kb	9	17	9	NITDKIDQI	NITDKIDQI	7.4
MHC-II	H2-IAb	3	17	15	PHDWTKNITDKIDQI	WTKNITDKI	19
MHC-II	H2-IAb	2	16	15	EPHDWTKNITDKIDQ	WTKNITDKI	19
MHC-II	H2-IAb	1	15	15	IEPHDWTKNITDKID	WTKNITDKI	23
MHC-II	H2-IAb	4	18	15	HDWTKNITDKIDQII	WTKNITDKI	31
MHC-II	H2-IAb	5	19	15	DWTKNITDKIDQIIH	WTKNITDKI	57
MHC-II	H2-IAb	6	20	15	WTKNITDKIDQIIHD	NITDKIDQI	60
MHC-II	H2-IAb	7	21	15	TKNITDKIDQIIHDF	ITDKIDQII	62
MHC-II	H2-IAb	10	24	15	ITDKIDQIIHDFVDK	IDQIIHDFV	67

Supplementary Table 9 | Predicted murine MHC epitopes of the Ebola HR2 peptide and associated MHC binding. NetMHCpan-4.1 was used to predict the top eight murine MHC-I and MHC-II epitopes of each HR2 peptide. The default cut-off for MHC binders was used as described by NetMHCpan[59]. A lower rank score for the peptide is associated with stronger binding to MHC. 0.5% was defined as the cut-off for 'strong' MHC-I binders while 2% was defined as the cut-off for 'weak' MHC-I binders. Any peptides that had a rank score >2% were assumed to not bind to MHC-I. 2% was defined as the cut-off for 'strong' MHC-II binders while 10% was defined as the cut-off for 'weak' MHC-II binders. Any peptides that had a rank score >10% were assumed to not bind to MHC-II.

Infectious disease	MHC-I strong binders	MHC-I weak binders	MHC-II strong binders	MHC-II weak binders
SARS-CoV-2	6	>8	0	4
HIV	2	>8	0	0
Ebola	0	1	0	0

Supplementary Table 10 | Summary Table of 'strong' and 'weak' MHC binding epitopes for various HR2 peptides. NetMHCpan-4.1 was used to predict the top eight murine MHC-I and MHC-II epitopes of each HR2 peptide. The default cutoff for MHC binders was used as described by NetMHCpan[59]. A lower rank score for the peptide is associated with stronger binding to MHC. 0.5% was defined as the cutoff for 'strong' MHC-I binders while 2% was defined as the cutoff for 'weak' MHC-I binders. Any peptides that had a rank score >2% were assumed to not bind to MHC-I. 2% was defined as the cutoff for 'strong' MHC-II binders while 10% was defined as the cutoff for 'weak' MHC-II binders. Any peptides that had a rank score >10% were assumed to not bind to MHC-II.

Antibody	Fluorophore	Catalog Number	Supplier
CD1c	BV421	331526	Biolegend
CD14	BUV395	563561	BD
CD86	BV605	305430	Biolegend
CD40	APC/Fire™ 750	334345	Biolegend
HLA-DR	PE-Cy7	307616	Biolegend
CD83	FITC	305306	Biolegend
ViaKrome 808 Fixable Viability Dye	ViaKrome 808	C36628	Beckman Coulter

Supplementary Table 11 | Antibodies and fluorescent reagents used for multiparametric flow cytometric analysis of human monocyte - derived dendritic cells (DCs). This panel includes markers for DC lineage (CD1c, CD14), activation and co-stimulation (CD86, CD40), antigen presentation (HLA-DR) and maturation (CD83), along with a fixable viability dye (ViaKrome 808) to exclude dead cells.

Antibody	Fluorophore	Catalog Number	Supplier
CD3	PerCP Cy5.5	317336	Biolegend
CD4	BV650	317436	Biolegend
CD8	AF700	344724	Biolegend
IFNγ	BV421	506538	Biolegend
TNFα	FITC	502906	Biolegend
IL2	PE-Cy7	500326	Biolegend
Mouse IgG1, κ Isotype Ctrl Antibody	BV421	400158	Biolegend
Mouse IgG1, κ Isotype Ctrl Antibody	FITC	400108	Biolegend
Rat IgG2a, κ Isotype Ctrl Antibody	PE-Cy7	400522	Biolegend
ViaKrome 808 Fixable Viability Dye	ViaKrome 808	C36628	Beckman Coulter

Supplementary Table 12 | Antibodies and reagents used for intracellular cytokine staining in human T cells by flow cytometry. This panel includes T cell lineage markers (CD3, CD4, CD8) to delineate T cell subsets; intracellular cytokines (IFNγ, TNFα, IL2) to assess functional responses; appropriate isotype control antibodies (mouse IgG1, κ and rat IgG2a, κ) for specificity; and a fixable viability dye (ViaKrome 808) to discriminate live cells.

Protein Name	Supplier	Catalog Number	Number of Lysines	Molecular Weight
HBsAg	Advanced Immunochemical Inc.	7-HbADW	4	25,394.7 g/mol
E8L	Sino Biological	40890-V08B	21	35,750 g/mol
H3L	Sino Biological	40893-V08H1	22	33,690 g/mol
M1R	Sino Biological	40904-V07H	10	21,590 g/mol
SARS-CoV-2 Spike	Sino Biological	40589-V08H8	61	136,450 g/mol

Supplementary Table 13 | Protein sources and relevant properties. The number of lysines for each protein is relevant to the protein-oligonucleotide conjugation reaction, as the oligonucleotide is conjugated to lysine residues via an NHS ester reaction with the amino group on the lysines, followed by a DBCO-azide click chemistry reaction.

HLA-A or HLA-DRB	Start	End	Length	Peptide	Core peptide region	Rank
HLA-A*02:01	19	27	9	RLNEVAKNL	RLNEVAKNL	0.16
HLA-A*02:01	26	34	9	NLNESLIDL	NLNESLIDL	0.18
HLA-A*02:01	30	37	8	SLIDLQEL	SLID-LQEL	0.37
HLA-A*02:01	25	34	10	KNLNESLIDL	KLNESLIDL	0.66
HLA-A*02:01	24	34	11	AKNLNESLIDL	ALNESLIDL	1.1
HLA-A*02:01	5	13	9	GINASVVNI	GINASVVNI	1.1
HLA-A*02:01	15	23	9	KEIDRLNEV	KEIDRLNEV	1.5
HLA-A*02:01	13	23	11	IQKEIDRLNEV	IQIDRLNEV	1.5
HLA-DRB1*01:11	1	15	15	GDISGINASVVNIQK	ISGINASVV	12
HLA-DRB1*01:11	14	28	15	QKEIDRLNEVAKNLN	IDRLNEVAK	26
HLA-DRB1*01:11	2	16	15	DISGINASVVNIQKE	INASVVNIQ	28
HLA-DRB1*01:11	3	17	15	ISGINASVVNIQKEI	INASVVNIQ	29
HLA-DRB1*01:11	13	27	15	IQKEIDRLNEVAKNL	IDRLNEVAK	32
HLA-DRB1*01:11	10	24	15	VVNIQKEIDRLNEVA	IQKEIDRLN	34
HLA-DRB1*01:11	12	26	15	NIQKEIDRLNEVAKN	IDRLNEVAK	35
HLA-DRB1*01:11	8	22	15	ASVVNIQKEIDRLNE	VNIQKEIDR	38

Supplementary Table 14 | Predicted human HLA epitopes of the SARS-CoV-2 HR2 peptide and associated HLA binding. NetMHCpan-4.1 was used to predict the top eight human HLA-A*02:01 and HLA-DRB1*01:11 epitopes of each HR2 peptide. The default cutoff for HLA binders was used as described by NetMHCpan[59]. A lower rank score for the peptide is associated with stronger HLA binding. 0.5% was defined as the cutoff for 'strong' HLA-A*02:01 binders while 2% was defined as the cutoff for 'weak' HLA-A*02:01 binders. Any peptides that had a rank score >2% were assumed to not bind to HLA-A*02:01. 2% was defined as the cutoff for 'strong' HLA-DRB1*01:11 binders while 10% was defined as the cutoff for 'weak' HLA-DRB1*01:11 binders. Any peptides that had a rank score >10% were assumed to not bind to HLA-DRB1*01:11. HLA-A*02:01 and HLA-DRB1*01:11 were chosen as representative human HLAs, as they are the most well-studied HLAs of each class.

HLA-A or HLA-DRB	Start	End	Length	Peptide	Core peptide region	Rank
HLA-A*02:01	38	47	10	LLELDKWASL	LLLDKWASL	1.2
HLA-A*02:01	16	23	8	YTSLIHSL	YTS-LIHSL	2.7
HLA-A*02:01	37	47	11	ELLELDKWASL	ELLELDASL	3.2
HLA-A*02:01	16	24	9	YTSLIHSLI	YTSLIHSLI	3.9
HLA-A*02:01	18	26	9	SLIHSLIEE	SLIHSLIEE	4.1
HLA-A*02:01	18	27	10	SLIHSLIEES	SLIHSLIES	4.3
HLA-A*02:01	22	30	9	SLIEESQNQ	SLIEESQNQ	4.7
HLA-A*02:01	15	23	9	NYTSLIHSL	NYTSLIHSL	4.9
HLA-DRB1*01:11	13	27	15	INNYTSLIHSLIEES	YTSLIHSLI	4.2
HLA-DRB1*01:11	12	26	15	EINNYTSLIHSLIEE	YTSLIHSLI	5.7
HLA-DRB1*01:11	14	28	15	NNYTSLIHSLIEESQ	YTSLIHSLI	9
HLA-DRB1*01:11	11	25	15	REINNYTSLIHSLIE	YTSLIHSLI	12
HLA-DRB1*01:11	17	31	15	TSLIHSLIEESQNQQ	IHSLIEESQ	29
HLA-DRB1*01:11	16	30	15	YTSLIHSLIEESQNQ	IHSLIEESQ	33
HLA-DRB1*01:11	15	29	15	NYTSLIHSLIEESQN	YTSLIHSLI	33
HLA-DRB1*01:11	10	24	15	DREINNYTSLIHSLI	YTSLIHSLI	34

Supplementary Table 15 | Predicted human HLA epitopes of the HIV HR2 peptide and associated HLA binding. NetMHCpan-4.1 was used to predict the top eight human HLA-A*02:01 and HLA-DRB1*01:11 epitopes of each HR2 peptide. The default cutoff for HLA binders was used as described by NetMHCpan[59]. A lower rank score for the peptide is associated with stronger HLA binding. 0.5% was defined as the cutoff for 'strong' HLA-A*02:01 binders while 2% was defined as the cutoff for 'weak' HLA-A*02:01 binders. Any peptides that had a rank score >2% were assumed to not bind to HLA-A*02:01. 2% was defined as the cutoff for 'strong' HLA-DRB1*01:11 binders while 10% was defined as the cutoff for 'weak' HLA-DRB1*01:11 binders. Any peptides that had a rank score >10% were assumed to not bind to HLA-DRB1*01:11. HLA-A*02:01 and HLA-DRB1*01:11 were chosen as representative human HLAs, as they are the most well-studied HLAs of each class.

HLA-A or HLA-DRB	Start	End	Length	Peptide	Core peptide region	Rank
HLA-A*02:01	13	22	10	KIDQIIHDFV	KIDQIIHFV	0.71
HLA-A*02:01	13	21	9	KIDQIIHDF	KIDQIIHDF	1.4
HLA-A*02:01	9	17	9	NITDKIDQI	NITDKIDQI	1.6
HLA-A*02:01	10	18	9	ITDKIDQII	ITDKIDQII	3.2
HLA-A*02:01	8	17	10	KNITDKIDQI	KITDKIDQI	4.5
HLA-A*02:01	10	17	8	ITDKIDQI	ITD-KIDQI	9.2
HLA-A*02:01	12	22	11	DKIDQIIHDFV	KIDQIIHFV	9.5
HLA-A*02:01	13	23	11	KIDQIIHDFVD	KIDQIIHFV	11
HLA-DRB1*01:11	3	17	15	PHDWTKNITDKIDQI	WTKNITDKI	8.6
HLA-DRB1*01:11	2	16	15	EPHDWTKNITDKIDQ	WTKNITDKI	9.7
HLA-DRB1*01:11	1	15	15	IEPHDWTKNITDKID	WTKNITDKI	17
HLA-DRB1*01:11	4	18	15	HDWTKNITDKIDQII	WTKNITDKI	20
HLA-DRB1*01:11	7	21	15	TKNITDKIDQIIHDF	ITDKIDQII	35
HLA-DRB1*01:11	10	24	15	ITDKIDQIIHDFVDK	IDQIIHDFV	36
HLA-DRB1*01:11	6	20	15	WTKNITDKIDQIIHD	ITDKIDQII	38
HLA-DRB1*01:11	5	19	15	DWTKNITDKIDQIIH	WTKNITDKI	43

Supplementary Table 16 | Predicted human HLA epitopes of the Ebola HR2 peptide and associated HLA binding. NetMHCpan-4.1 was used to predict the top eight human HLA-A*02:01 and HLA-DRB1*01:11 epitopes of each HR2 peptide. The default cutoff for HLA binders was used as described by NetMHCpan[59]. A lower rank score for the peptide is associated with stronger HLA binding. 0.5% was defined as the cutoff for ‘strong’ HLA-A*02:01 binders while 2% was defined as the cutoff for ‘weak’ HLA-A*02:01 binders. Any peptides that had a rank score >2% were assumed to not bind to HLA-A*02:01. 2% was defined as the cutoff for ‘strong’ HLA-DRB1*01:11 binders while 10% was defined as the cutoff for ‘weak’ HLA-DRB1*01:11 binders. Any peptides that had a rank score >10% were assumed to not bind to HLA-DRB1*01:11. HLA-A*02:01 and HLA-DRB1*01:11 were chosen as representative human HLAs, as they are the most well-studied HLAs of each class.

Infectious disease	HLA-A strong binders	HLA-A weak binders	HLA-DRB strong binders	HLA-DRB weak binders
SARS-CoV-2	3	>8	0	0
HIV	0	1	0	3
Ebola	0	3	0	2

Supplementary Table 17 | Summary table of 'strong' and 'weak' HLA binding epitopes for various HR2 peptides. NetMHCpan-4.1 was used to predict the top eight human HLA-A*02:01 and HLA-DRB1*01:11 epitopes of each HR2 peptide. The default cutoff for HLA binders was used as described by NetMHCpan[59]. A lower rank score for the peptide is associated with stronger HLA binding. 0.5% was defined as the cutoff for 'strong' HLA-A*02:01 binders while 2% was defined as the cutoff for 'weak' HLA-A*02:01 binders. Any peptides that had a rank score >2% were assumed to not bind to HLA-A*02:01. 2% was defined as the cutoff for 'strong' HLA-DRB1*01:11 binders while 10% was defined as the cutoff for 'weak' HLA-DRB1*01:11 binders. Any peptides that had a rank score >10% were assumed to not bind to HLA-DRB1*01:11. HLA-A*02:01 and HLA-DRB1*01:11 were chosen as representative human HLAs, as they are the most well-studied HLAs of each class.

Antibody	Fluorophore	Catalog Number	Supplier
I-A/I-E (MHC II)	Brilliant Violet 421™	107632	Biolegend
CD11b	Brilliant Violet 605™	101257	Biolegend
CD103	Alexa Fluor® 700	121442	Biolegend
CD86	PE/Cyanine7	105014	Biolegend
CD205 (DEC-205)	PerCP/Cyanine5.5	138208	Biolegend
CD11c	Brilliant Violet 510™	117338	Biolegend
CD3	Brilliant Violet 785™	100232	Biolegend
CD4	PE	100408	Biolegend
CD8a	APC/Cyanine7	100714	Biolegend
CD107a (LAMP-1)	Alexa Fluor® 488	121608	Biolegend
CD279 (PD-1)	PerCP/Cyanine5.5	135208	Biolegend
IFN-γ	Brilliant Violet 510™	505841	Biolegend
CD69	PE/Cyanine7	104512	Biolegend
NK-1.1	PE	156504	Biolegend
T-bet	Brilliant Violet 421™	644832	Biolegend
CD40	PE	124610	Biolegend
CD64	FITC	139316	Biolegend

Supplementary Table 18 | Antibodies used for flow cytometry in Cov-2-Spike DoriVac immunogenicity studies. This panel was used to characterize immune cell phenotypes and activation in S-protein-conjugated DoriVac-treated mice and includes markers for antigen-presenting cells (I-A/I-E, CD11b, CD11c, CD40, CD64, CD86, CD205), T cells (CD3, CD4, CD8a, CD69, CD279, IFN-γ, T-bet), NK cells (NK-1.1, CD107a), and additional markers (CD103).

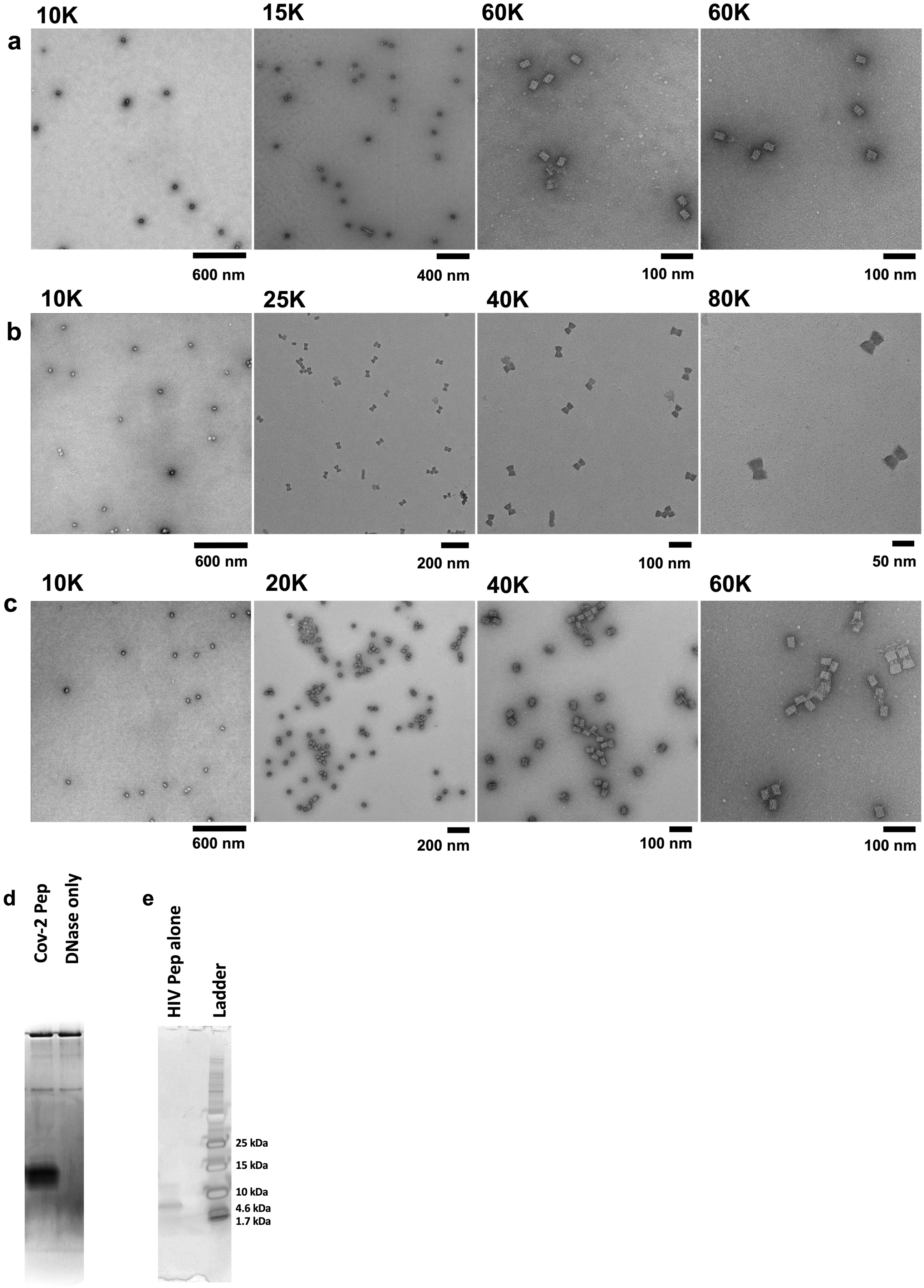

Supplementary Fig. 1 | Additional TEM images of HR2-functionalized DoriVac nanoparticles and SDS-PAGE analysis of associated peptides. a, Negative stain TEM image of DoriVac nanoparticles demonstrated that the nanoparticles were monodispersed, further supporting the agarose gel results. **b,**

Negative stain TEM image of HIV nanoparticles demonstrated that the nanoparticles were majority monodispersed, with some dimers observed, further supporting the agarose gel results. **c,** Negative stain TEM image of Ebola nanoparticles demonstrated that the nanoparticles were majority monodispersed, with some dimers observed, further supporting the agarose gel results. **d,** SDS-PAGE gel showing DNase I only alongside DNase I digested Cov-2 peptide. **e,** SDS-PAGE gel showing HIV peptide alone, with two bands located near the 10 kDa ladder marker.

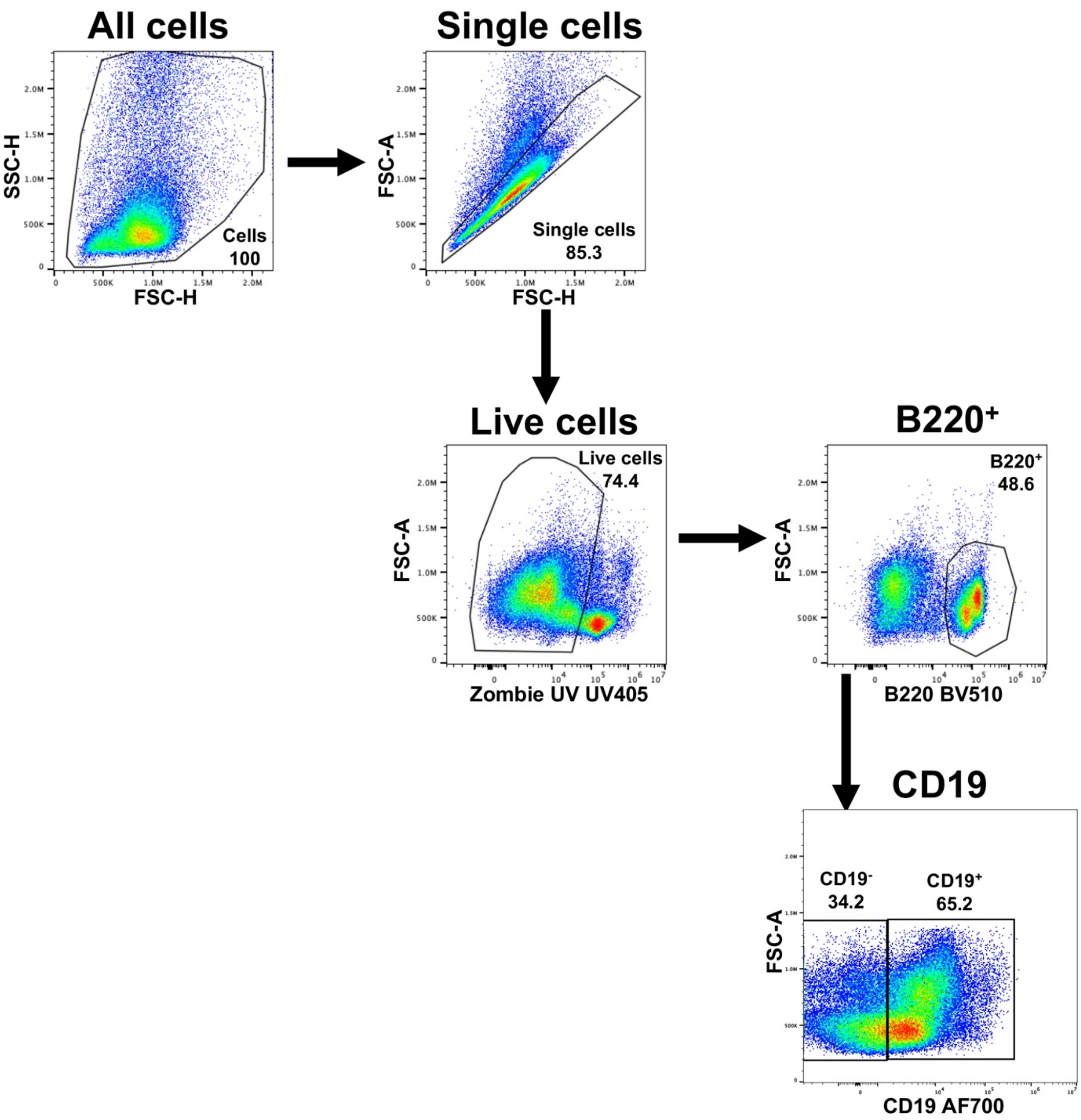

Supplementary Fig. 2 | B cell gating strategies. B cells were analysed using flow cytometry. This figure demonstrates the gating strategy to analyse B cells from frozen PBMC samples.

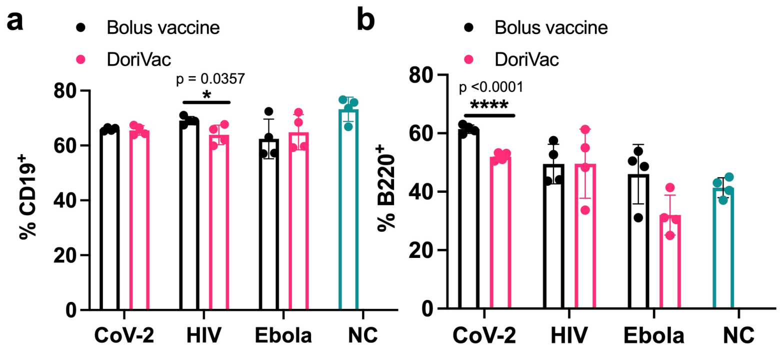

Supplementary Fig. 3 | Additional data demonstrating robust humoral responses for B cells. a, The $CD19^+$ B cell population in the blood on Day 21, as determined by flow cytometry, showed no significant difference after bolus vaccine treatment compared with DoriVac treatment. **b,** The $B220^+$ B cell population in the blood on Day 21, as determined by flow cytometry, showed minimal difference after bolus vaccine treatment compared with DoriVac treatment. Data are represented as mean ± SD. The flow data were analyzed by multiple unpaired t-tests and significance was defined as a two-tailed p value less than 0.05. '*' refers to $p \leq 0.05$; '****' refers to $p \leq 0.0001$.

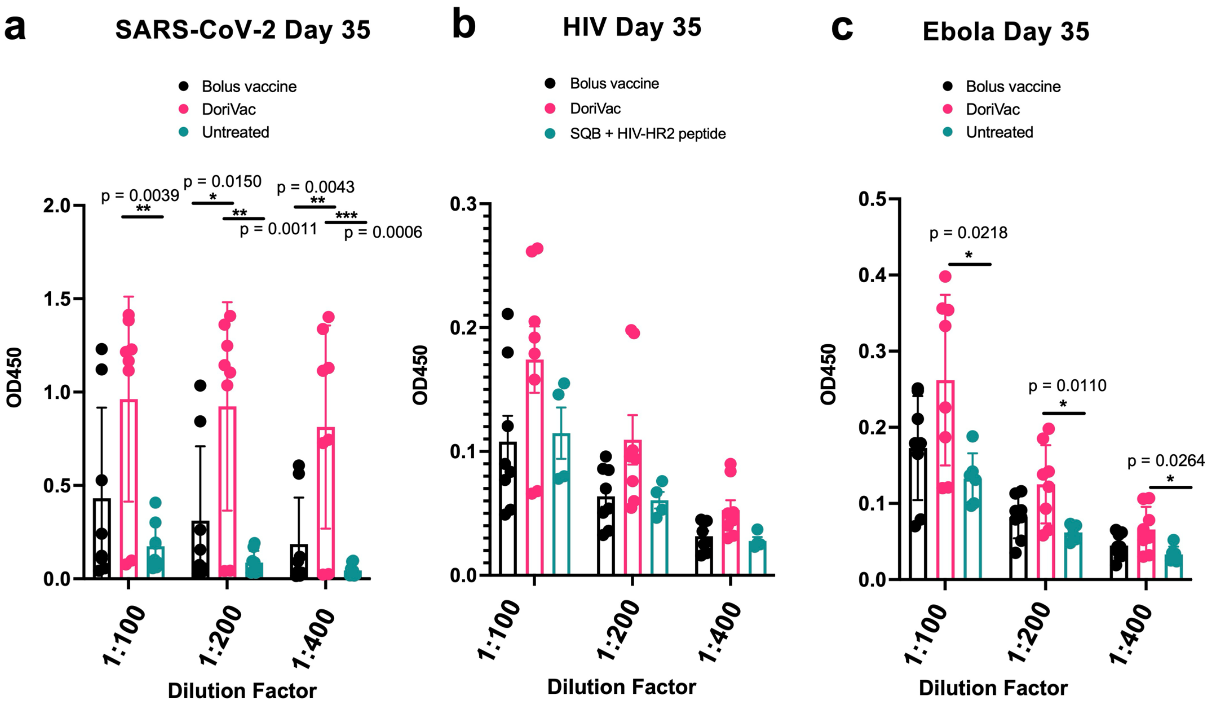

Supplementary Fig. 4 | Additional data demonstrating robust IgG antibody production. a, DoriVac treatment enhanced SARS-CoV-2 peptide-specific IgG antibody production in the plasma, as determined by ELISA assay, after two doses of the vaccine (on Day 35) compared to a bolus vaccine of free peptide and free CpG. **b,** DoriVac treatment enhanced HIV-peptide-specific IgG antibody production in the plasma, as determined by ELISA assay, after two doses of the vaccine (on Day 35) compared to a bolus vaccine. **c,** DoriVac treatment enhanced Ebola-peptide-specific IgG antibody production in the plasma, as determined by ELISA assay, after two doses of the vaccine (on Day 35) compared to a bolus vaccine. Data has been normalized. Data are represented as mean ± SD. The ELISA data was analyzed by one-way ANOVA (with correction for multiple comparisons using a Tukey's test) and significance was defined as a multiplicity-adjusted p value less than 0.05 (n=8). '*' refers to $P \leq 0.05$; '**' refers to $P \leq 0.01$. '***' refers to $P \leq 0.001$.

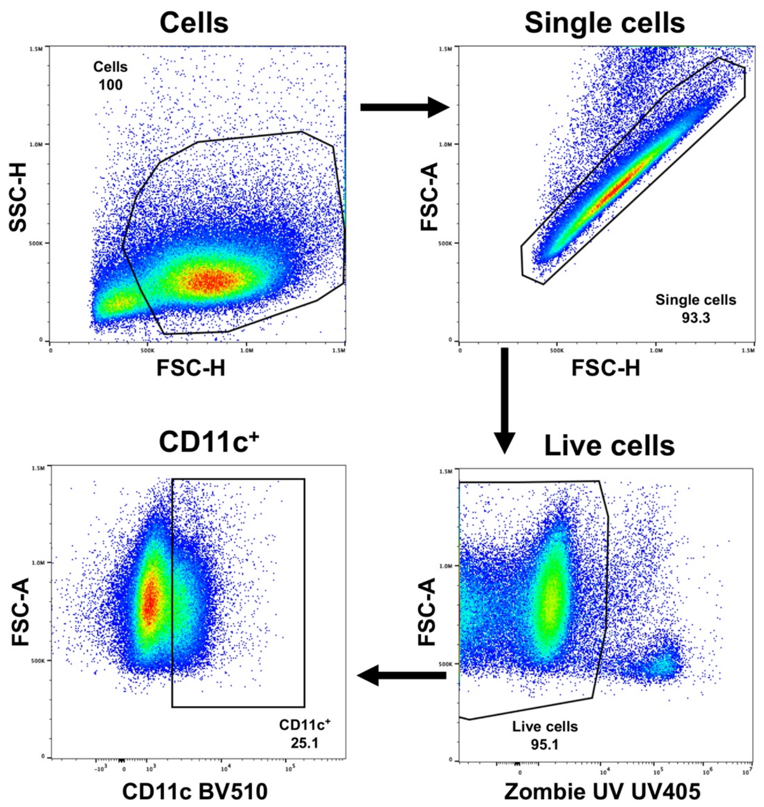

Supplementary Fig. 5 | DC gating strategies for lymph node samples. Dendritic cells were analyzed using flow cytometry. This figure demonstrates the gating strategy to analyze dendritic cells collected from the lymph nodes.

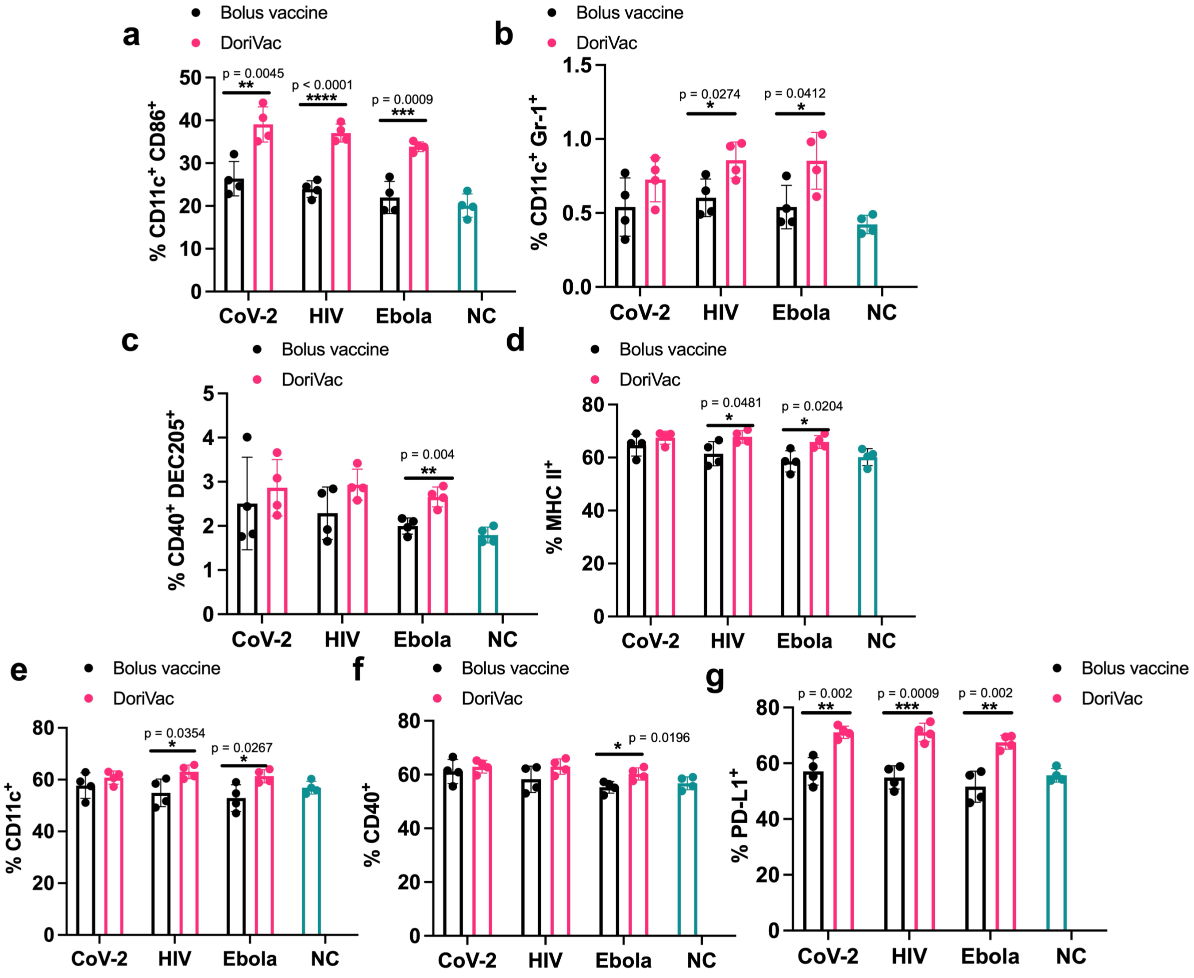

Supplementary Fig. 6 | Additional data demonstrating DC responses in the splenocytes on Day 21. Spleens were collected on Day 21 after two doses of treatment and processed into single-cell suspensions for flow cytometry. **a,** Percentages of $CD11c^+$ $CD86^+$, as determined by flow cytometry, in the splenocyte population (n=4). DoriVac demonstrated a significant increase in this activated DC population compared to bolus-vaccine treatment. **b,** Percentages of $CD11c^+$ Gr-1^+ DCs, as determined by flow cytometry, in the splenocyte population (n=4). DoriVac treatment demonstrated an increase in this plasmacytoid DC (pDC) –like subpopulation compared to bolus-vaccine treatment. **c,** Percentages of $CD40^+$ $DEC205^+$ DCs, as determined by flow cytometry, in the splenocytes (n=4). DoriVac treatment demonstrated an increase in this activated, endocytic subpopulation compared to bolus-vaccine treatment. **d,** Percentages of MHC-II^+ DCs, as determined by flow cytometry, in the draining lymph node (n=4). DoriVac treatment demonstrated a significant increase in this activated DC population compared to bolus-vaccine treatment. **e,** DoriVac treatment led to a notable increase in the $CD11c^+$ dendritic-cell population, as determined by flow cytometry. **f,** The lymphocytes were more activated (as indicated by upregulation of $CD40^+$) after DoriVac treatment compared to bolus vaccine treatment, as determined by flow cytometry. **g,** An increase in the PD-$L1^+$ subpopulation was observed after DoriVac treatment, compared to bolus-vaccine treatment, as determined by flow cytometry. Data are represented as mean ± SD. The flow data were analyzed by multiple unpaired t-tests and significance was defined as a two-tailed p value less than 0.05. '*' refers to $p \le 0.05$; '**' refers to $p \le 0.01$; '***' refers to $p \le 0.001$; '****' refers to $p \le 0.0001$.

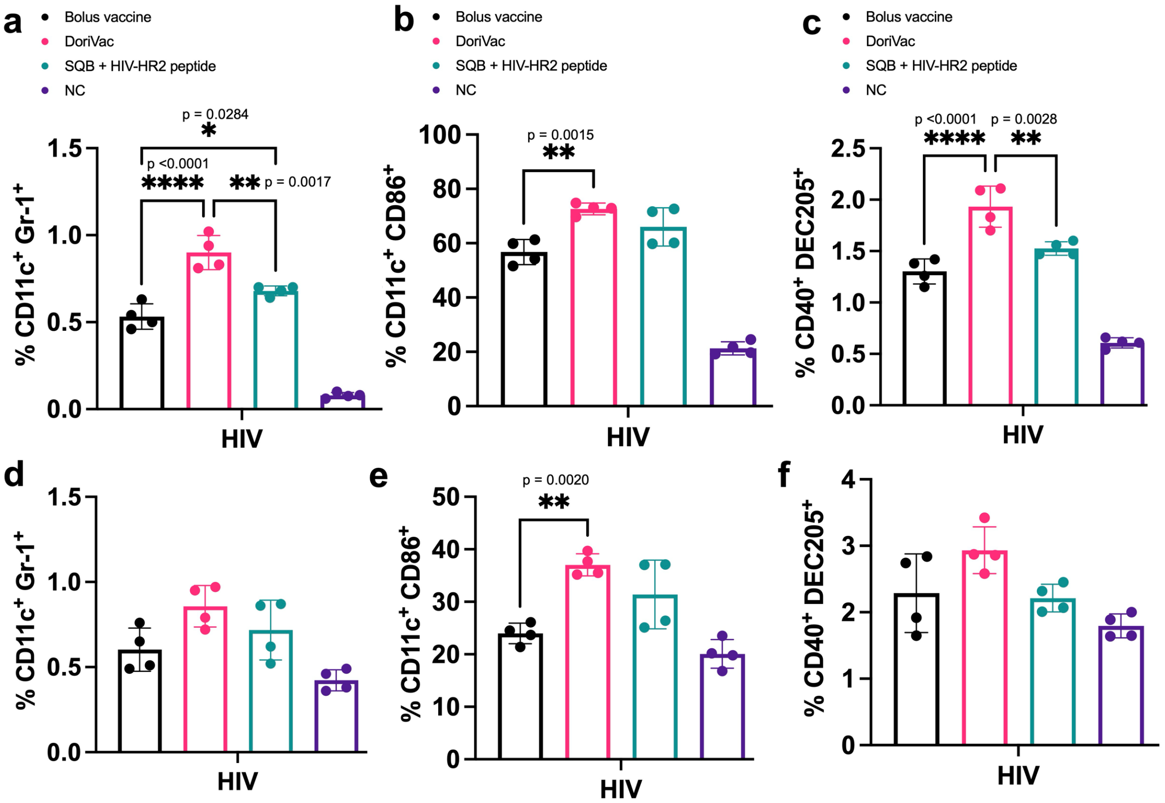

Supplementary Fig. 7 | Co-delivery of CpG and peptide on SQB is critical to robust DC responses. Half of the mice were sacrificed on Day 21 and draining lymph nodes were collected. **a,** Percentages of human pDC-like (CD11c$^+$ Gr-1$^+$) DCs in the draining lymph node (n=4), as determined by flow cytometry. DoriVac treatment demonstrated a significant increase in this plasmacytoid DC (pDC) – like subpopulation compared to treatment with the CpG SQB and HIV peptide, administered as a solution. **b,** Percentages of activated cDCs (CD11c$^+$ CD86$^+$) in the draining lymph node, as determined by flow cytometry. DoriVac treatment showed significant activation compared to the bolus vaccine and slightly more than the DNA origami SQB and HIV peptide administered as separate components. **c,** Percentages of CD40$^+$ DEC205$^+$ DCs in the draining lymph node (n=4), as determined by flow cytometry. The DoriVac treatment demonstrated a significant increase in this activated, endocytic subpopulation compared to bolus vaccine treatment and more than the DNA origami SQB and HIV peptide administered as separate components. **d,** Percentages of human pDC-like DCs in the splenocytes on Day 21 (n=4), as determined by flow cytometry. DoriVac treatment shows a significant increase compared to the bolus control. **e,** Percentages of activated cDCs in the splenocyte population, as determined by flow cytometry. DoriVac induced a significant increase in this population compared to the bolus control. **f,** Percentages of activated, endocytic DCs in the splenocytes population, as determined by flow cytometry. DoriVac showed significant increase in this population compared to the DNA origami SQB and HIV peptide delivered separately. Data are represented as mean ± SD. The flow data was analyzed by one-way ANOVA (with correction for multiple comparisons using a Tukey's test) and significance was defined as a multiplicity-adjusted p value less than 0.05 (n=4). '*' refers to $P \leq 0.05$; '**' refers to $P \leq 0.01$; '***' refers to $P \leq 0.001$; '****' refers to $P \leq 0.0001$.

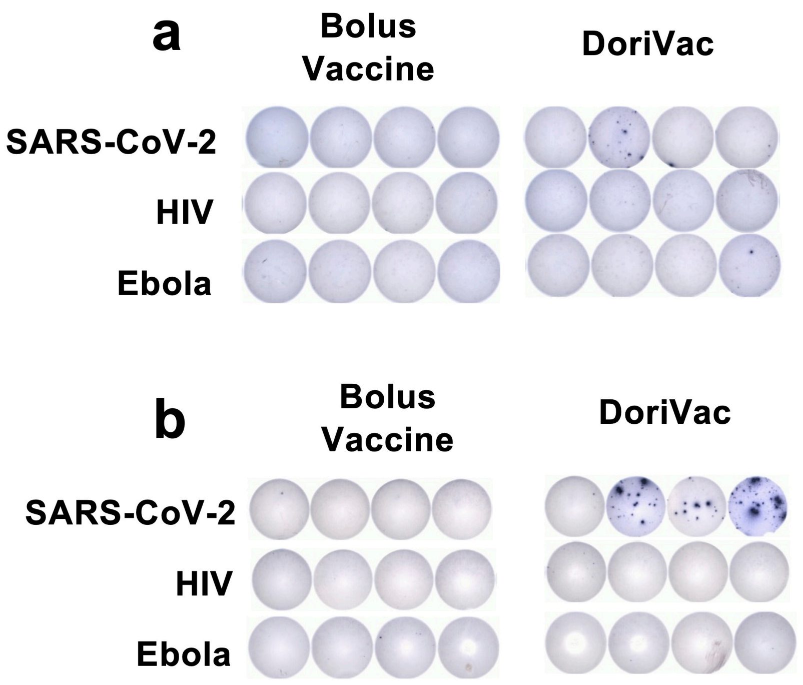

Supplementary Fig. 8 | Additional data on antigen-specific T cell responses on Day 21 and 28. a, Interferon-gamma (IFNγ) ELISpot demonstrating frequency of antigen-specific splenocytes on Day 21. Minimal difference is observed between the bolus-vaccine treated group and the DoriVac-treated group. **b,** IFNγ ELISpot demonstrating frequency of antigen-specific PBMCs on Day 28. There is a notable increase in the frequency of SARS-CoV-2 antigen-specific T cells.

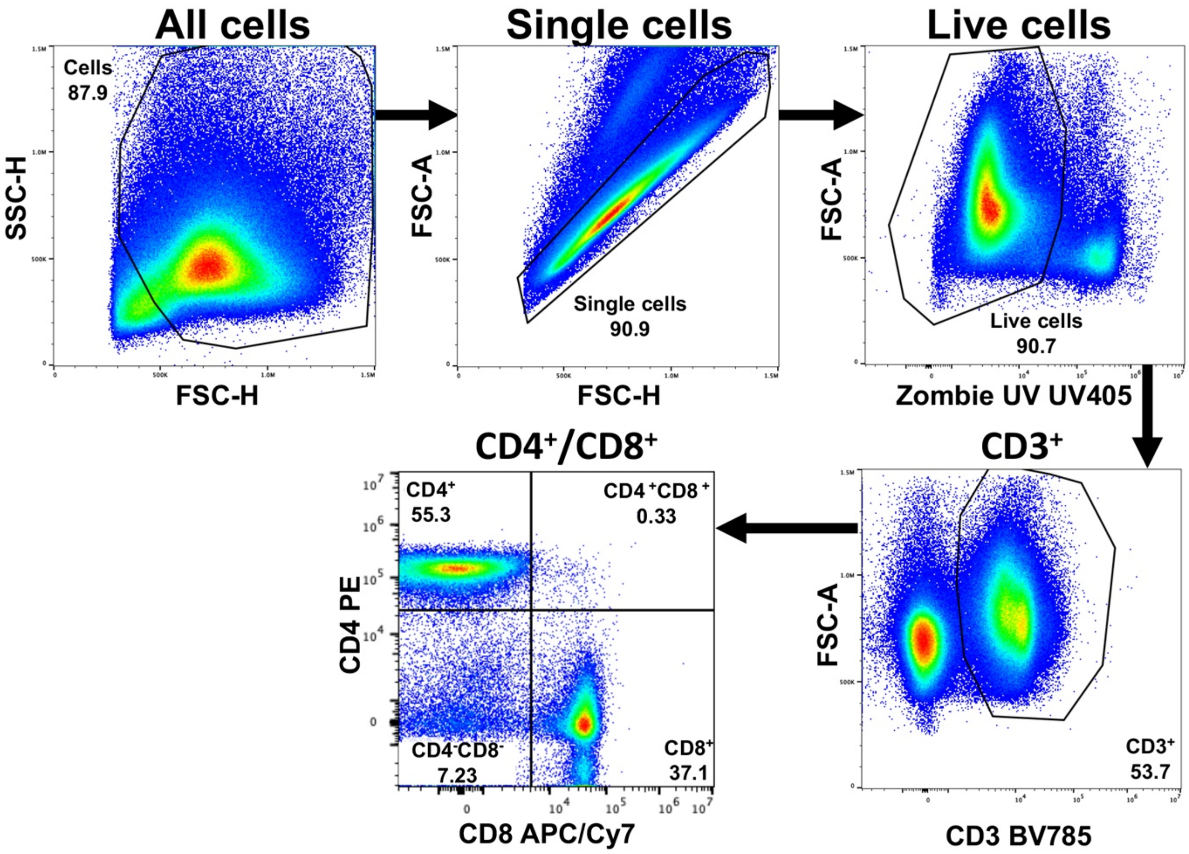

Supplementary Fig. 9 | T cell gating strategies. T cells were analyzed using flow cytometry. This figure demonstrates the gating strategy to analyze T cells in lymph nodes and spleen. Images shown are from the lymph nodes.

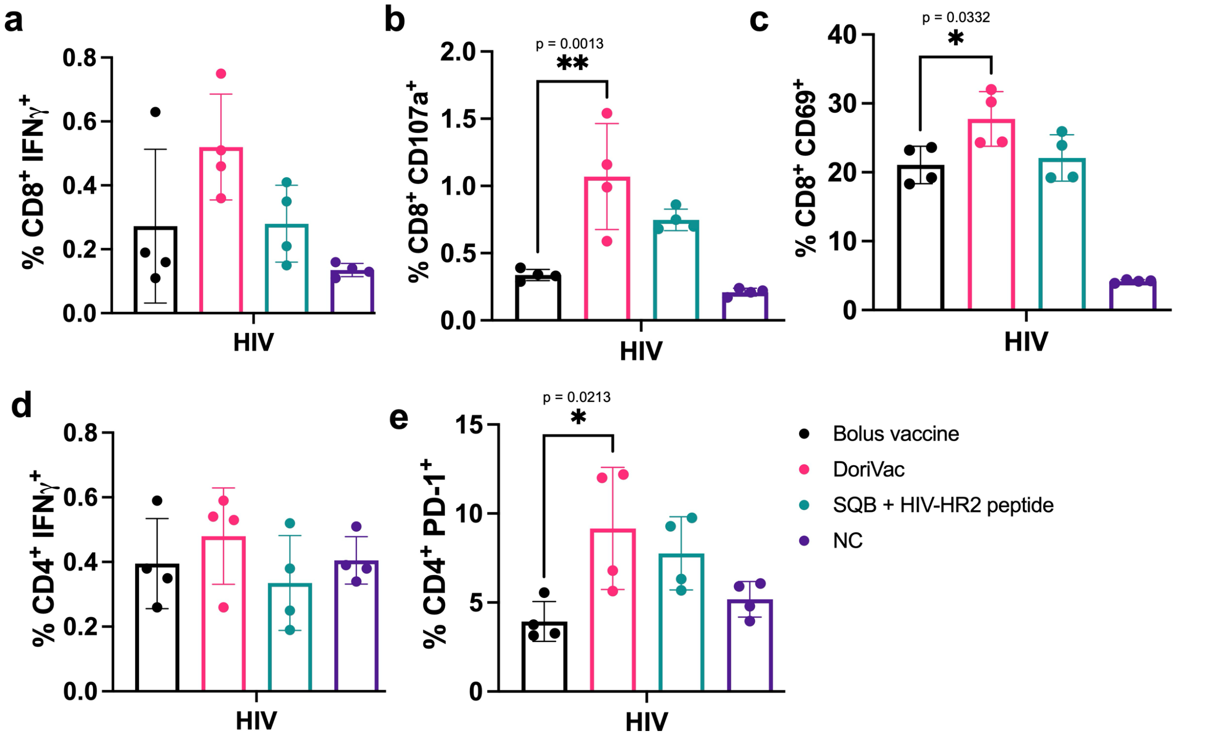

Supplementary Fig. 10 | Co-delivery of CpG and antigen peptide is essential to robust T cell responses. a, IFN-γ+ CD8+ T cells in the lymph node (LN) were upregulated on Day 21 in the DoriVac treatment group, compared to the bolus vaccine or the DNA origami SQB with HIV peptide groups, as determined by flow cytometry. **b,** CD107a+ CD8+ cytotoxic T cells in the LN were upregulated on Day 21 in the DoriVac treatment group, compared to the bolus vaccine or the DNA origami SQB with HIV peptide groups, as determined by flow cytometry. **c,** CD69+ CD8+ activated T cells in the LN showed upregulation on Day 21 after treatment with DoriVac, as determined by flow cytometry. **d,** IFN-γ+ CD4+ T cells in the LN were upregulated in the context of DoriVac treatment group on Day 21, compared to the DNA origami SQB with HIV peptide group, as determined by flow cytometry. **e,** PD-1+ CD4+ T cells in the LN were upregulated in the context of the DoriVac treated group on Day 21, compared to the bolus vaccine and DNA origami SQB with HIV peptide group, as determined by flow cytometry. Data are represented as mean ± SD. The flow data was analyzed by one-way ANOVA (with correction for multiple comparisons using a Tukey's test) and significance was defined as a multiplicity-adjusted p value less than 0.05 (n=4). '*' refers to P ≤ 0.05; '**' refers to P ≤ 0.01.

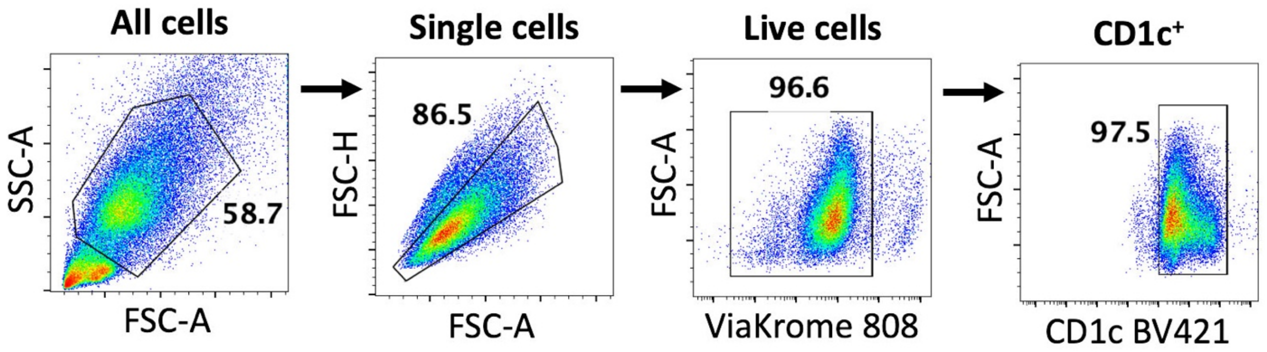

Supplementary Fig. 11 | Activation of human monocyte-derived dendritic cells (moDCs) analysis using flow cytometry. This figure demonstrates the gating strategy to analyse cells collected from moDCs after treatment with DNA origami vaccines.

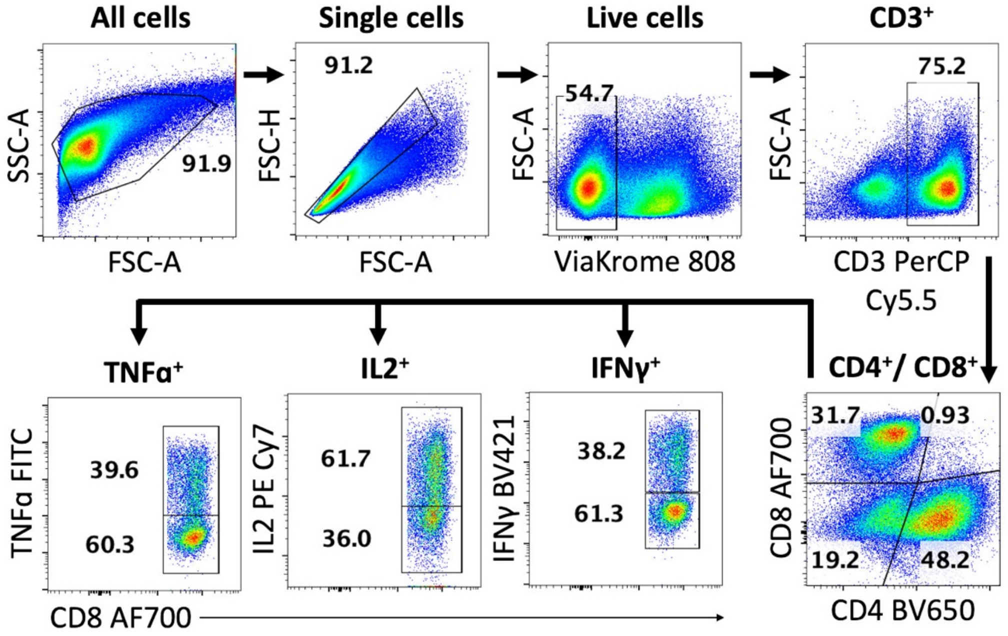

Supplementary Fig. 12 | Intracellular cytokine staining analysis using flow cytometry for cells from LN organ-on-a-chips. This figure demonstrates the gating strategy to analyse cells collected from LN organ-on-a-chips.

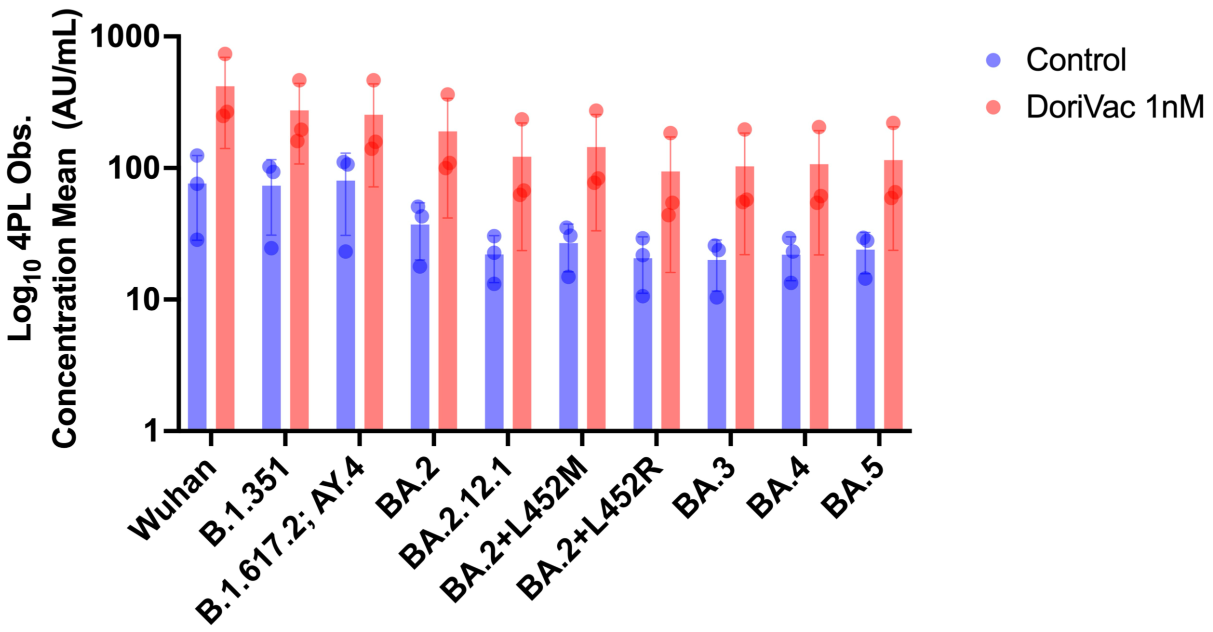

Supplementary Fig. 13 | Broad antibody response induced by HR2-DoriVac in a human lymph-node-on-chip model against SARS-CoV-2 spike variants. Concentration of antibodies (log10, 4PL fit observed) measured by Meso Scale Discovery (MSD) assay against SARS-CoV-2 spike protein from different variants of concern. Each bar represents the mean antibody concentration across three independent chips (n=3) in the presence of DoriVac (1 nM, red bars) and untreated control (blue bars). Variants include Wuhan, B.1.351, B.1.617.2; AY.4 Alt Seq 2, BA.2, BA.2.12.1, BA.2+L452M, BA.2+L452R, BA.3, BA.4, and BA.5. DoriVac treatment elicited higher antibody responses compared to the control across all tested variants, demonstrating a broad and robust humoral immune response.

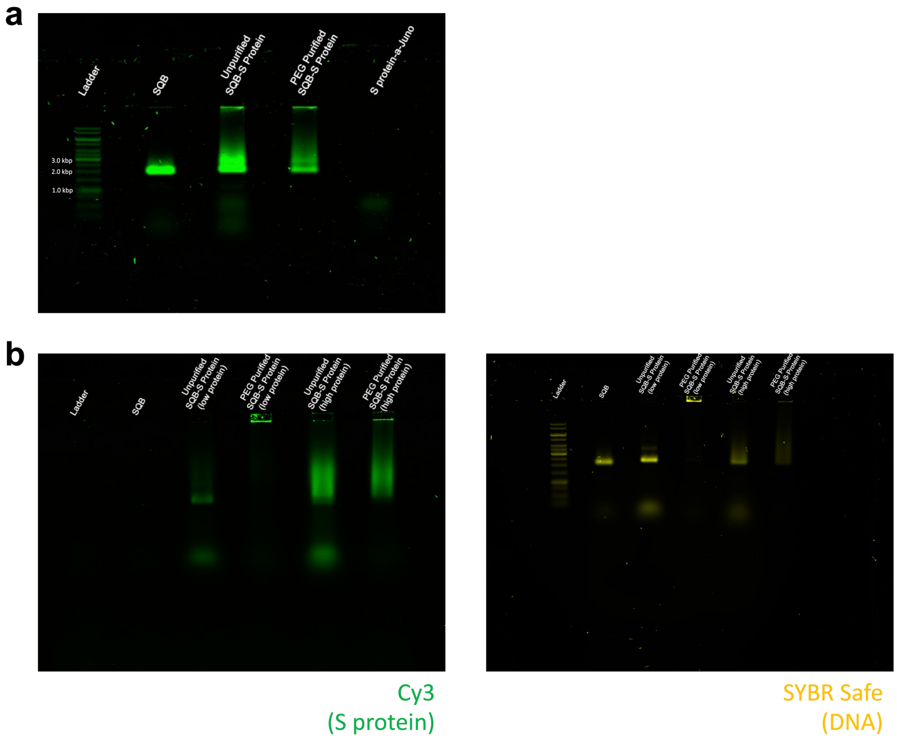

Supplementary Fig. 14 | Conjugation of Cov-2 Spike S Protein to DoriVac SQB DNA origami. a, Agarose gel electrophoresis demonstrating the successful attachment of oligonucleotide-conjugated S-proteins (S protein-a-Juno) to SQB origamis, resulting in upward band shifts in both the unpurified and PEG-purified DoriVac samples. PEG-purified samples were used for subsequent animal studies. **b,** Agarose gel electrophoresis demonstrating the complete removal of excess unbound oligonucleotide-conjugated Cy3-labelled Cov-2 spike S proteins from S-protein-conjugated SQBs after PEG purification (left, Cy3 green channel). The gel was subsequently post stained with SYBR safe to visualize the DNA origamis and oligonucleotides (right, yellow channel). We tested oligonucleotide-conjugated proteins added to SQBs in 40x excess of the amount of SQBs (high protein) and 11x (low protein).

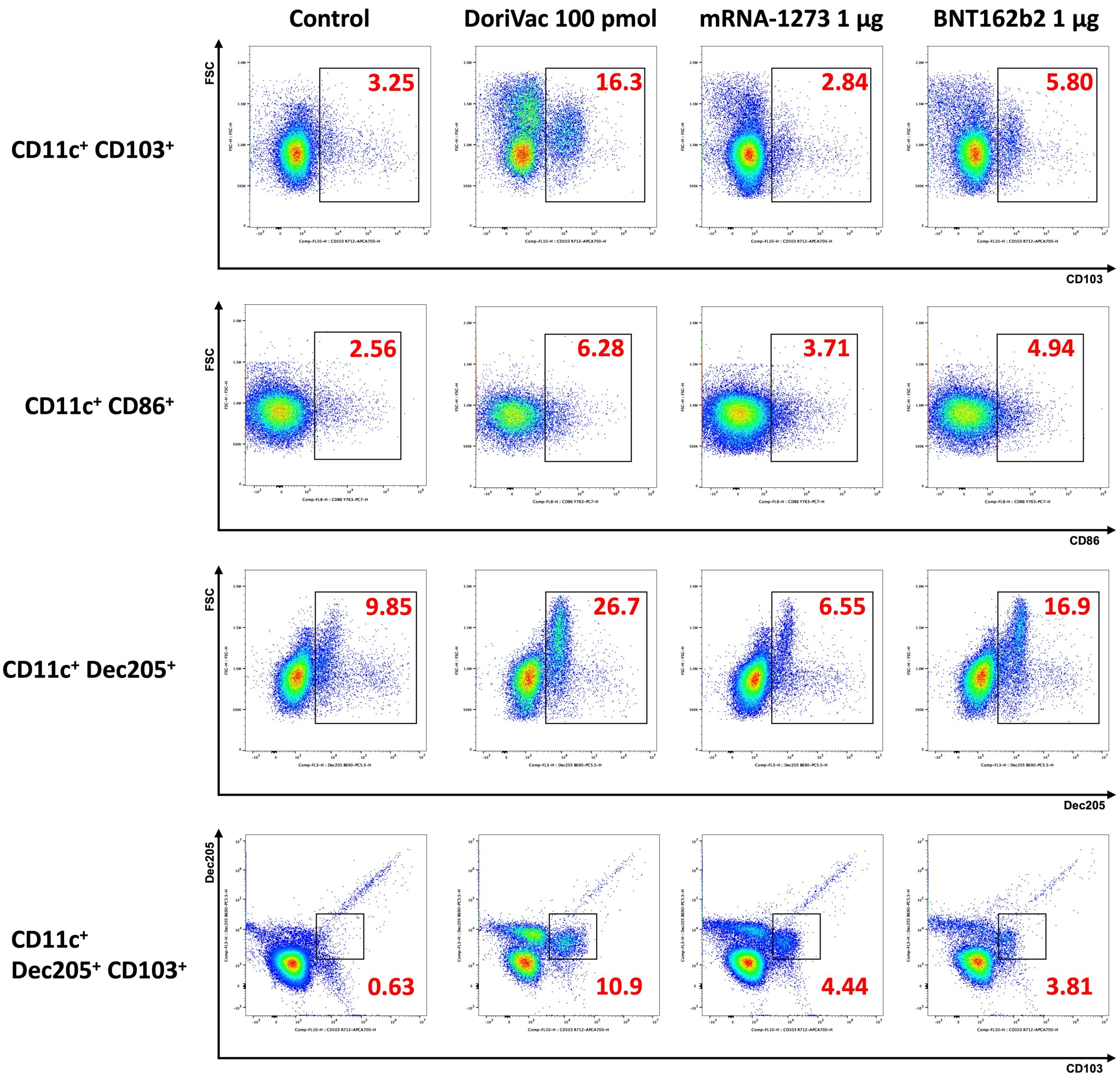

Supplementary Fig. 15 | Immune activation in CD11c$^+$ PBMCs on day 22, one day after the second dose. Representative images from flow scatter plots and corresponding frequencies of CD11c$^+$ population. Gating strategy is shown in Supplementary Fig. 5.

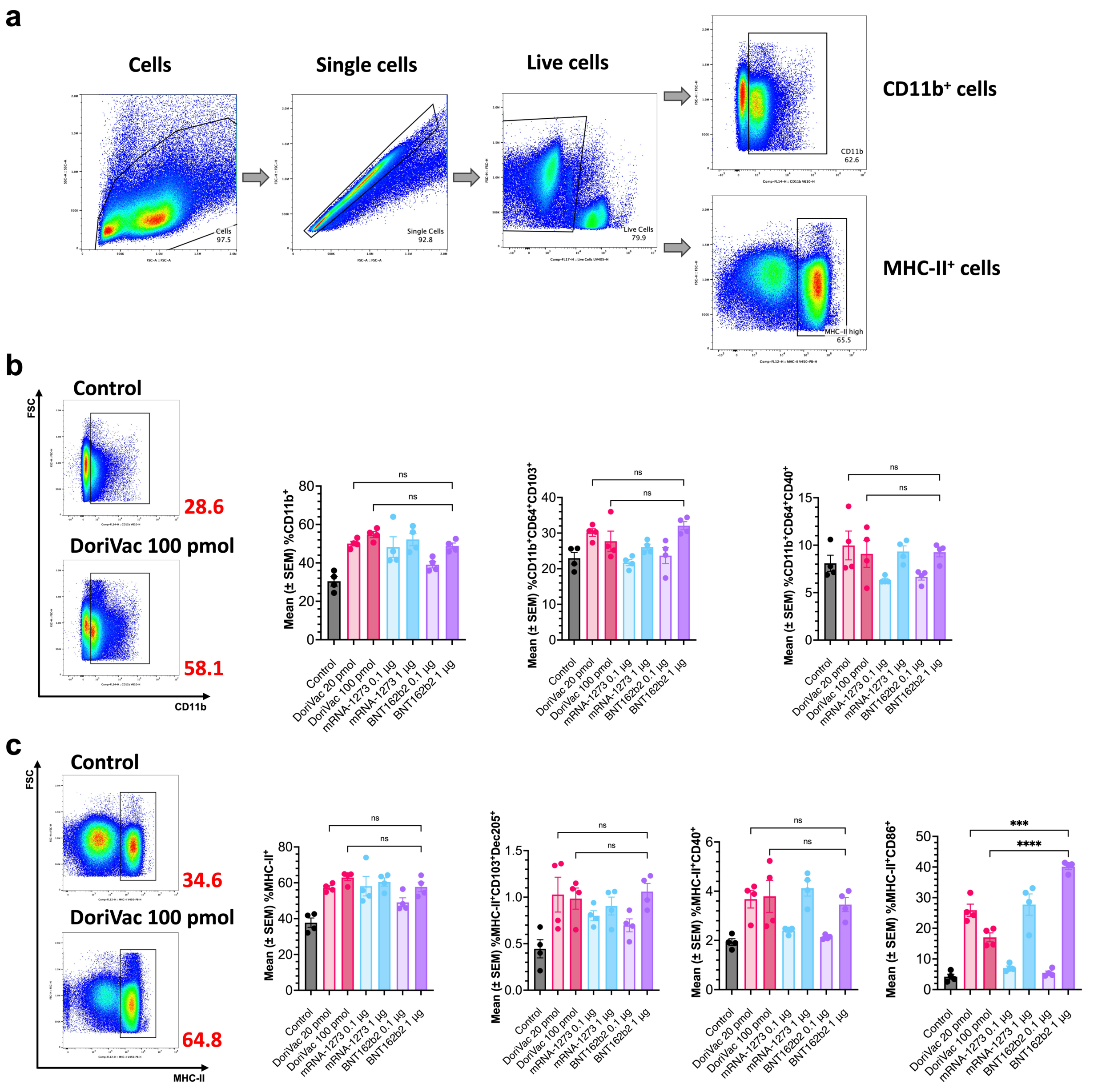

Supplementary Fig. 16 | Immune activation in the lymphatic cell population on day 50, one day after the third dose. a, Cell gating strategy for CD11b⁺ myeloid cells and MHC-II⁺ APCs in the lymph node (LN). **b,** CD11b⁺ myeloid cells and cross-presenting dendritic cells in the LN were upregulated on day 50 in the DoriVac treatment groups, compared to the untreated control, as determined by flow cytometry. DoriVac treatment groups showed responses comparable to the mRNA-LNP groups. Representative images from flow scatter plots and corresponding frequencies of CD11b⁺ population are shown on left. **c,** MHC-II⁺ APCs in the LN were upregulated on day 50 in the DoriVac treatment groups, compared to the untreated control, as determined by flow cytometry. DoriVac treatment groups showed responses comparable to mRNA-LNP groups for most APC activation markers except the BNT162b2 1 μg group for MHC-II⁺ CD86⁺ APCs. Representative images from flow scatter plots and corresponding frequencies of MHC-II⁺ population are shown on left. The flow data was analyzed by one-way ANOVA (with correction for multiple comparisons using a Tukey's test) and significance was defined as a multiplicity-adjusted p value less than 0.05 (n=4). 'ns' refers to $P > 0.05$; '***' refers to $P \leq 0.001$; '****' refers to $P \leq 0.0001$.

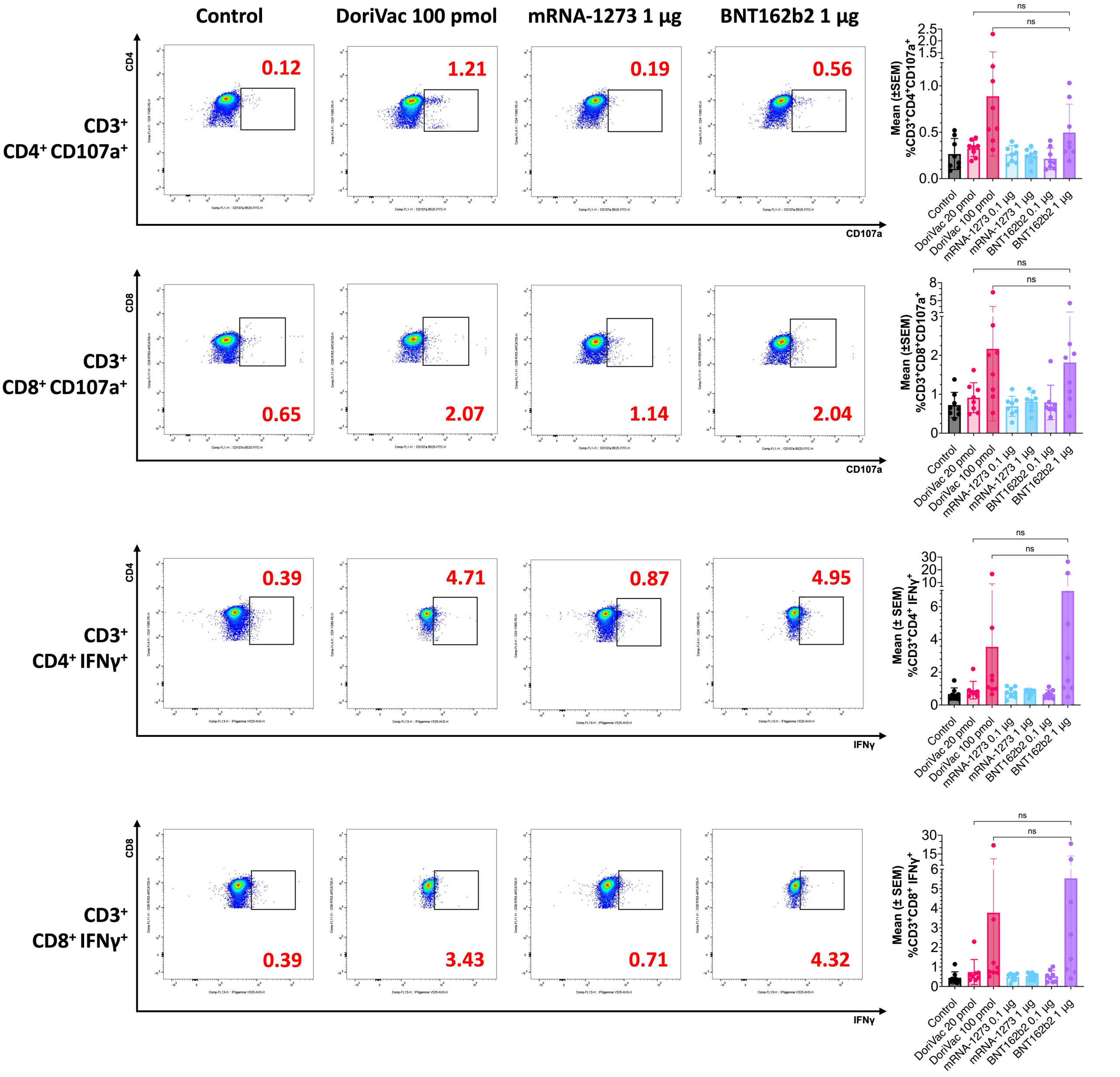

Supplementary Fig. 17 | Percentages of CD107⁺ and IFNγ-secreting PBMCs on day 22, one day after the second dose. Percentages of CD107⁺ PBMCs (n=8) and IFNγ secreting PBMCs (n=8) on day 22 as determined by flow cytometry. Gating strategy is illustrated in Supplementary Fig. 9. Representative images from flow scatter plots and corresponding frequencies are shown on the left. CD107a⁺ CD4⁺ and CD8⁺ PBMCs were upregulated on day 22 in the DoriVac treatment groups, compared to the untreated control. DoriVac treatment groups showed responses comparable to the mRNA-LNP groups. The flow data was analyzed by one-way ANOVA (with correction for multiple comparisons using a Tukey's test) and significance was defined as a multiplicity-adjusted p value less than 0.05 (n=8). 'ns' refers to P > 0.05.

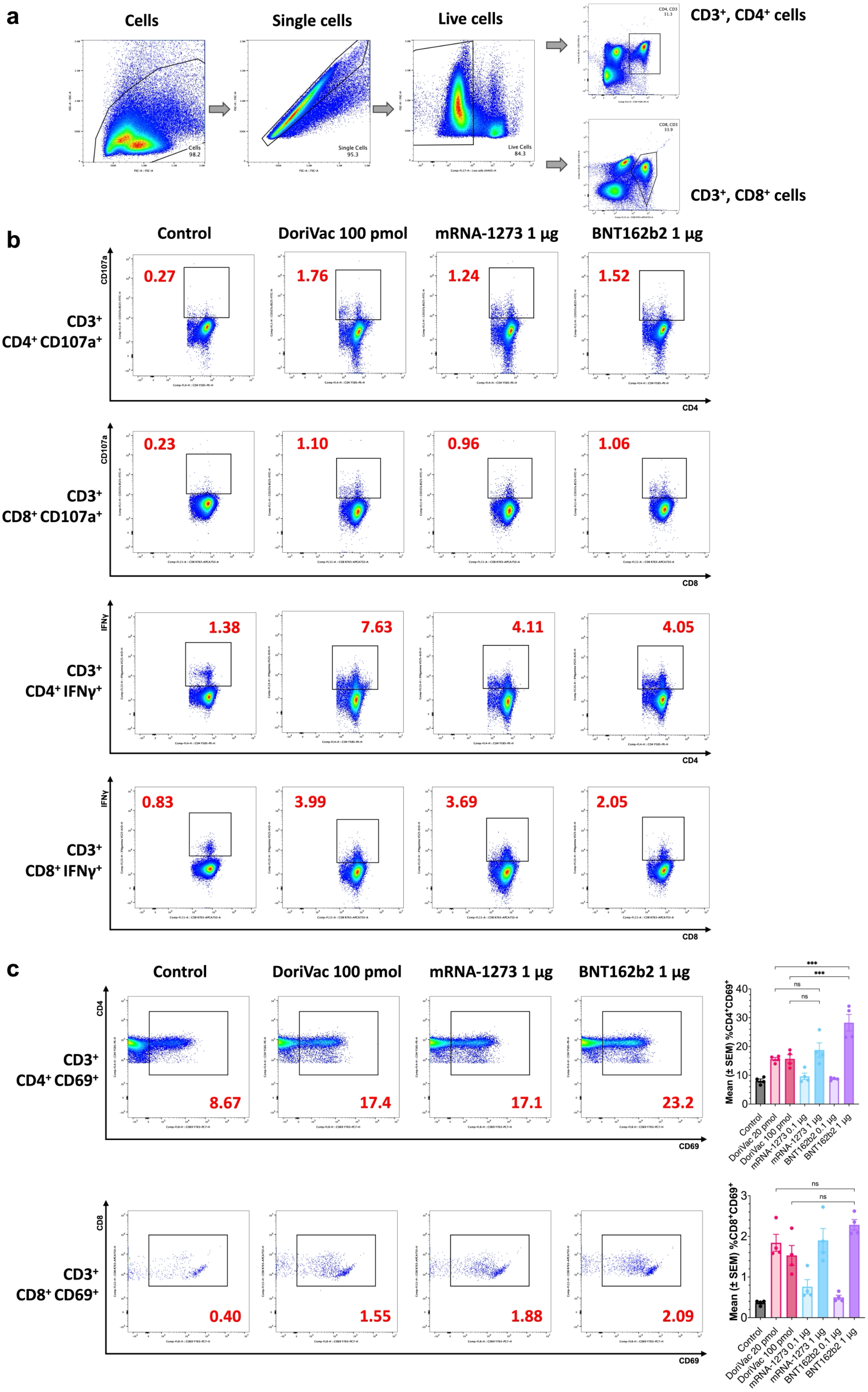

Supplementary Fig. 18 | Immune activation in CD4⁺ and CD8⁺ effector and IFNγ-secreting T cells on day 50, one day after the third dose. a, Gating strategy is modified from that illustrated in Supplementary Fig. 9 for the day 50 LN sample to allow for a better separation of CD4⁺ and CD8⁺ LN cells. **b,**

Representative images from flow scatter plots and corresponding frequencies of $CD4^{+}$ and $CD8^{+}$ effector and IFNγ-secreting populations. **c,** $CD69^{+}$ $CD4^{+}$ and $CD8^{+}$ cells (n=4) in the lymph node (LN) were upregulated on day 50 in the DoriVac treatment groups, compared to the untreated control, as determined by flow cytometry. DoriVac treatment groups showed responses comparable to mRNA-LNP groups except the BNT162b2 1 µg group for $CD4^{+}CD69^{+}$ T cells. Representative images from flow scatter plots and corresponding frequencies are shown on the left. The flow data was analyzed by one-way ANOVA (with correction for multiple comparisons using a Tukey's test) and significance was defined as a multiplicity-adjusted p value less than 0.05 (n=4). 'ns' refers to $P > 0.05$; '***' refers to $P \leq 0.001$.

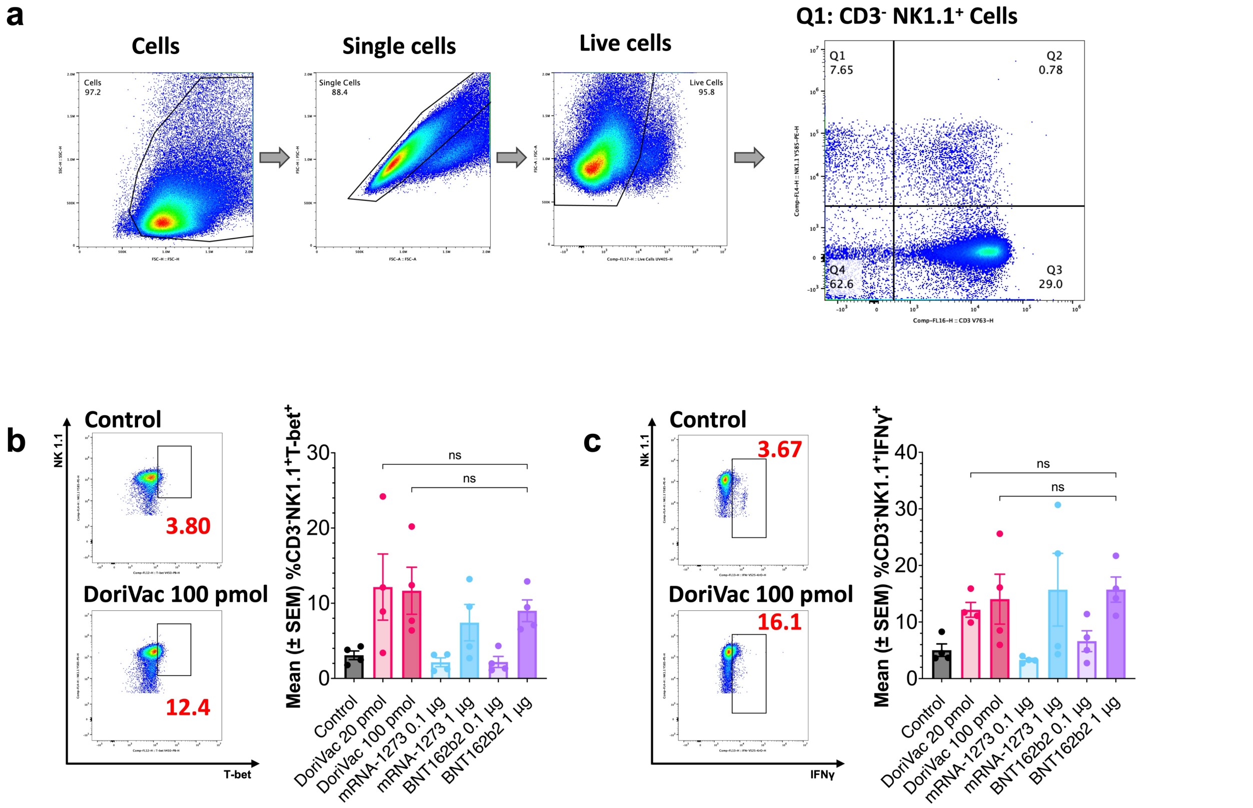

Supplementary Fig. 19 | Immune activation in PBMC NK Cells on day 50, one day after the third dose. a, Cell gating strategy for CD3$^-$NK1.1$^+$ PBMCs. **b–c,** T-bet and IFNγ expression in NK1.1$^+$ PBMC NK cells on day 50 in DoriVac-treated groups indicates enhanced NK cell activation and proinflammatory response compared to untreated control, as determined by flow cytometry (n=4). DoriVac treatment groups showed responses comparable to mRNA-LNP groups. Representative images from flow scatter plots and corresponding frequencies of population are shown on left. The flow data was analyzed by one-way ANOVA (with correction for multiple comparisons using a Tukey's test) and significance was defined as a multiplicity-adjusted p value less than 0.05 (n=4). 'ns' refers to $P > 0.05$.

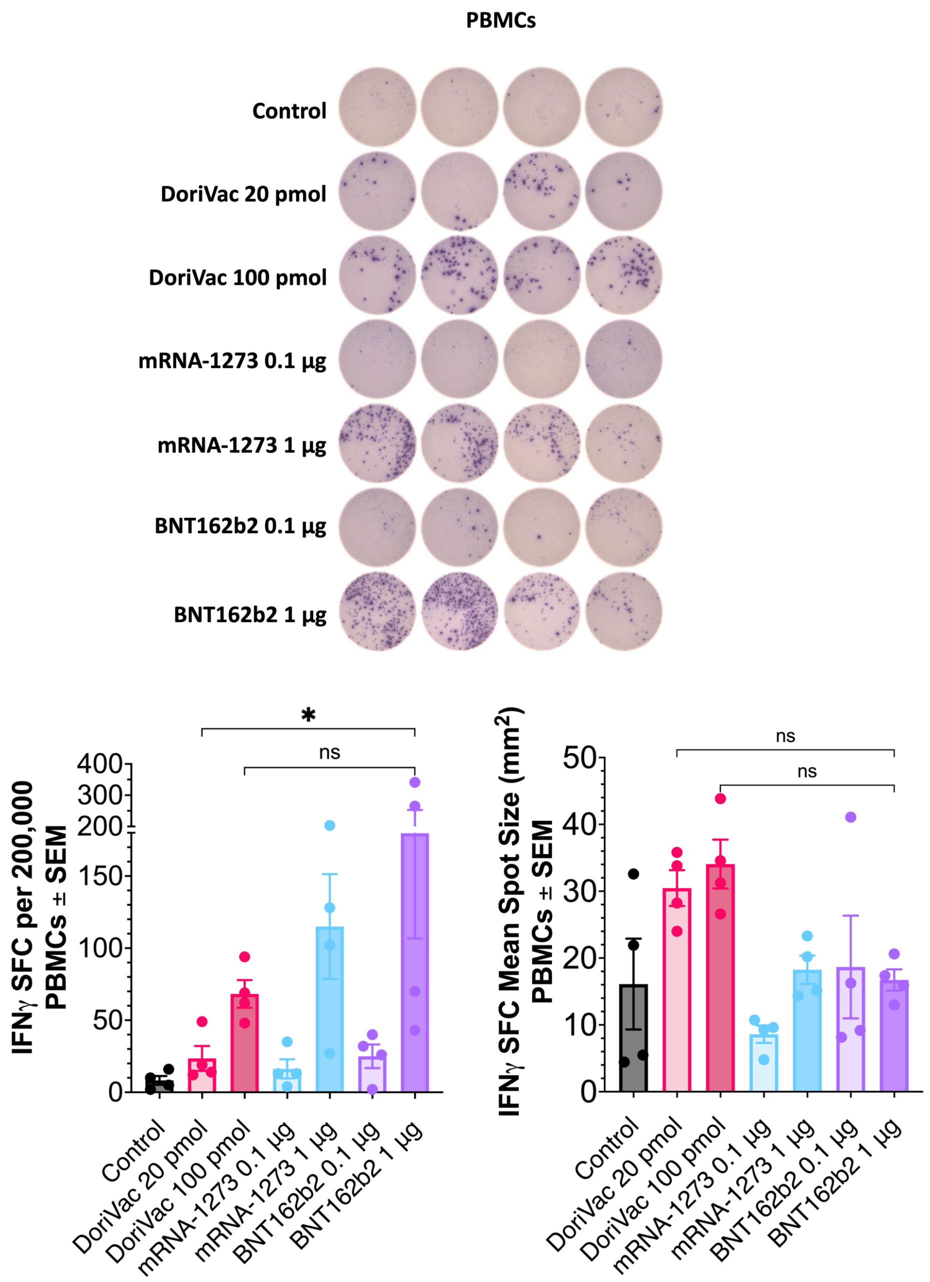

Supplementary Fig. 20 | SARS-CoV-2-specific T cell response following DoriVac treatment as measured by IFNγ ELISpot on day 35, two weeks after the second dose. IFNγ ELISpot demonstrating frequency of antigen-specific PBMCs (left, n=4, day 35), with accompanying quantification of IFNγ ELISpot SFUs and mean spot sizes. The results demonstrate an increase in SARS-COV-2 antigen-specific T cell frequency after treatment with DoriVac compared to the control. DoriVac treatment groups showed responses comparable to mRNA-LNP groups, only except when comparing the lower DoriVac dose to the best performing BNT162b2 1 µg group. The data was analyzed by one-way ANOVA (with correction for multiple comparisons using a Tukey's test) and significance was defined as a multiplicity-adjusted p value less than 0.05 (n=4). 'ns' refers to $P > 0.05$; '*' refers to $P \leq 0.05$.

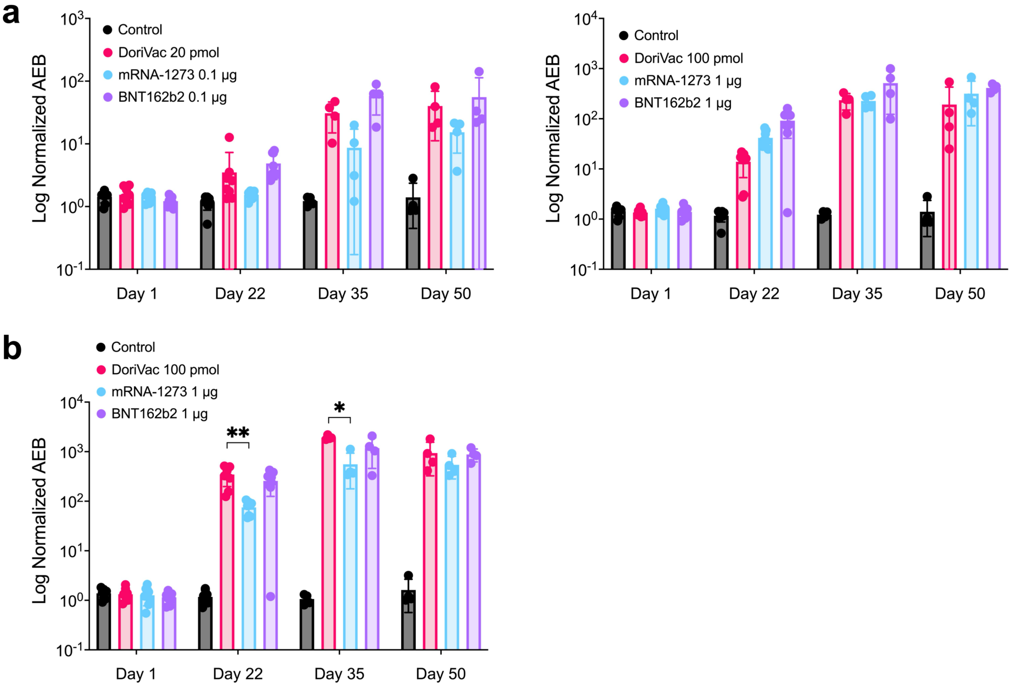

Supplementary Fig. 21 | Anti-S1 and anti-spike IgG production in different treatment groups as measured by SiMoA. a, Quantification of relative anti-S1 IgG levels in plasma samples using SiMoA (n=8 on day 1 and day 22, n=4 on day 35 and day 50). DoriVac treatment group exhibited increased antibody levels compared to the control across all sampling dates. **b,** Quantification of relative anti-spike IgG levels in plasma samples of control and 100 pmol/1 µg treatment groups using SiMoA (n=8 on day 1 and day 22, n=4 on day 35 and day 50). DoriVac treatment group exhibited increased antibody levels compared to the control across all sampling dates. The data was analyzed by one-way ANOVA (with correction for multiple comparisons using a Tukey's test) and significance was defined as a multiplicity-adjusted p value less than 0.05. '*' refers to $P \leq 0.05$; '**' refers to $P \leq 0.01$.

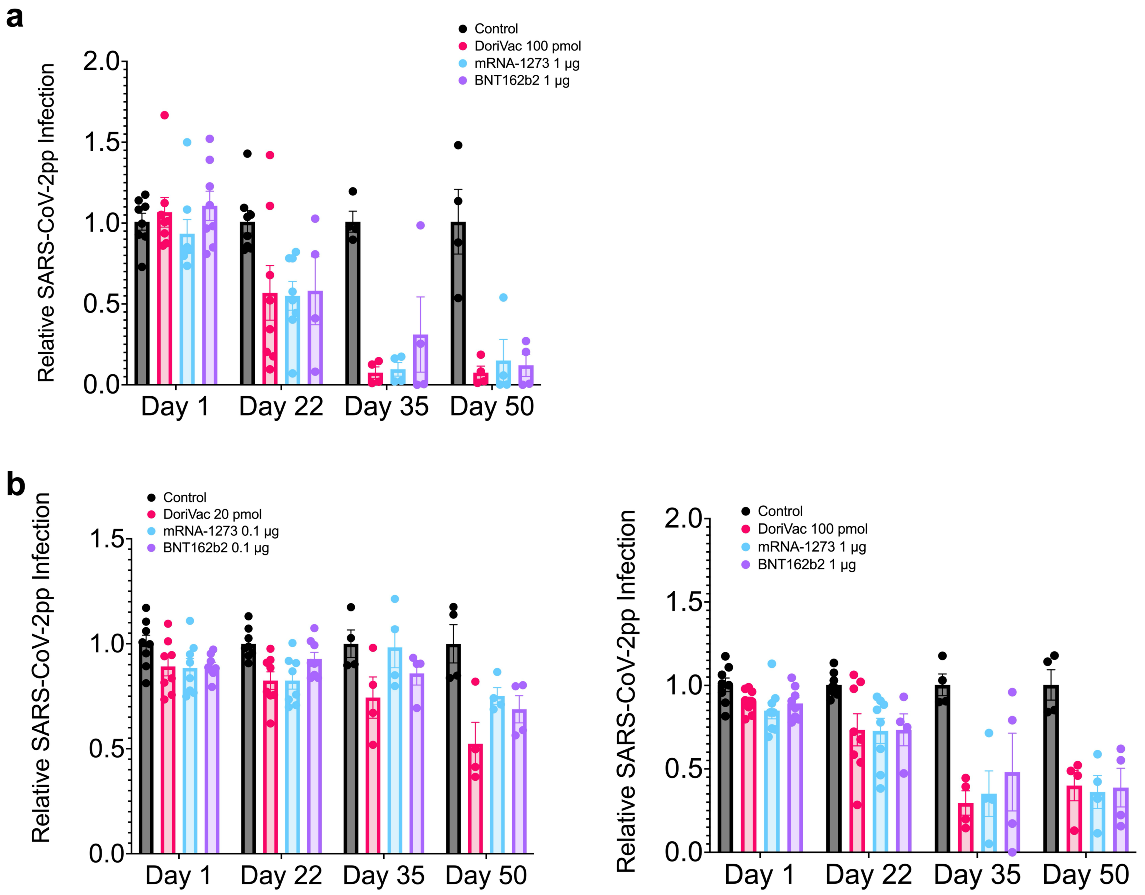

Supplementary Fig. 22 | SARS-CoV-2 pseudovirus neutralization by different treatments in ACE2-293T cells at low and high doses. a, SARS-CoV-2 pseudovirus (SARS-CoV-2pp) neutralization assay (1:20 dilution; n=8 on day 1 and day 22, n=4 on day 35 and day 50, except for BNT162b2 1 µg for which n=8 on Day 1 and n=4 on Day 22, 35, 50) for 100 pmol/1 µg treatment groups in model cell line ACE2-293T. DoriVac exhibited more pseudovirus neutralization compared to control starting on day 22. **b,** SARS-CoV-2 pseudovirus (SARS-CoV-2pp) neutralization assays for both 20 pmol/0.1 µg and 100 pmol/1 µg treatment groups were repeated with a 100x serum dilution for all samples. DoriVac continued to show stronger (20 pmol) or comparable (100 pmol) neutralization compared to mRNA-LNP vaccines.

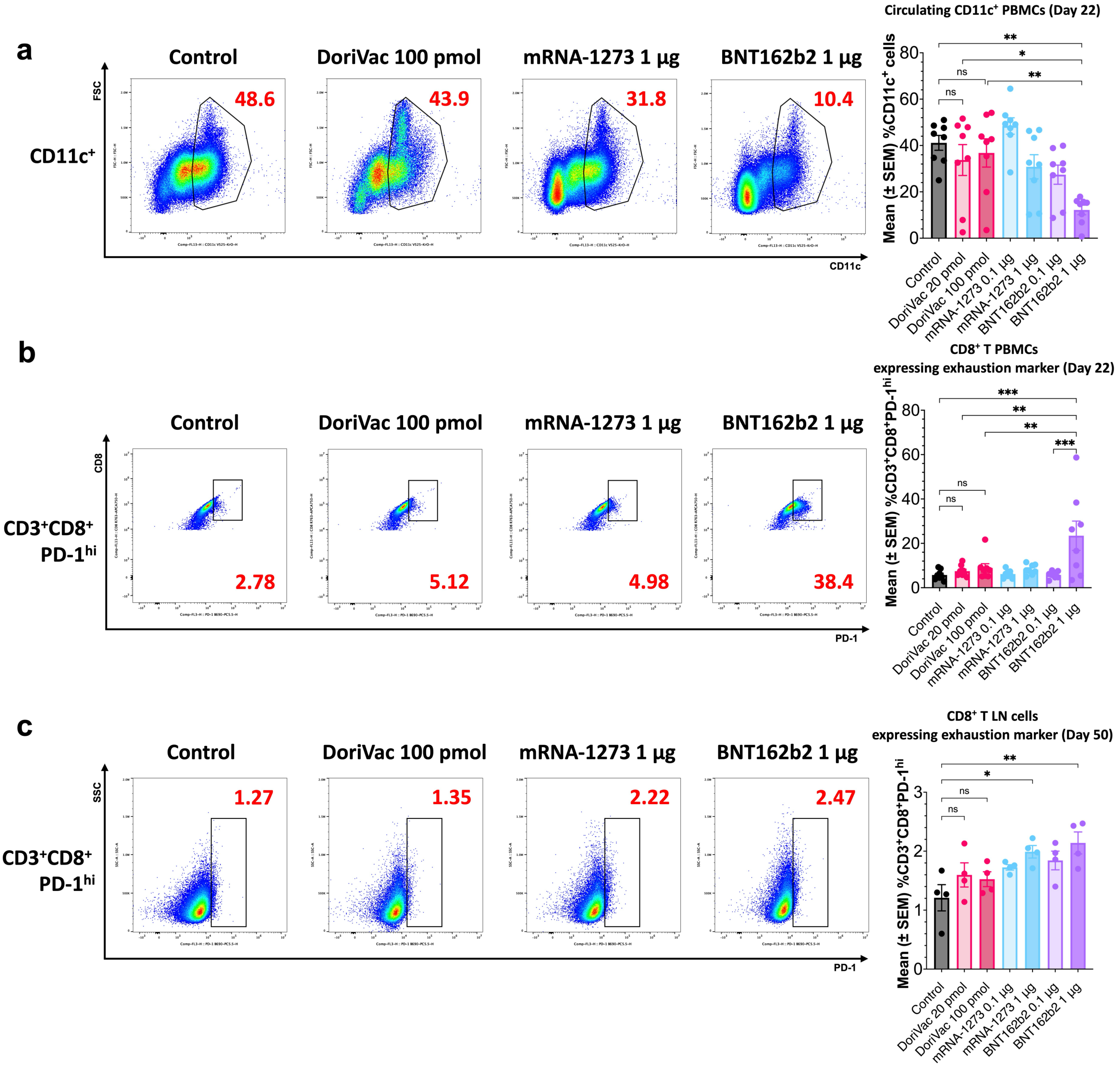

Supplementary Fig. 23 | Differential immune exhaustion in PBMCs and LN cells across treatment groups. a, Frequency of circulating CD11c$^+$ PBMCs on day 22 across treatment groups. Representative images from flow scatter plots and corresponding frequencies of CD11c$^+$ population are shown on the left. Gating strategy is shown in Supplementary Fig. 5. DoriVac treatment groups showed sustained activation, while BNT162b2 1 µg dose exhibited significant reductions compared to the control. **b,** CD8$^+$ T cells in PBMCs expressing the exhaustion marker PD-1 on day 22. Representative images from flow scatter plots and corresponding frequencies are shown on the left. Gating strategy is shown in Supplementary Fig. 9. The DoriVac 100 pmol dose group demonstrated significantly lower exhaustion compared to the BNT162b2 1 µg dose group. **c,** CD8$^+$ T cells from LN expressing PD-1 on day 50. Representative images from flow scatter plots and corresponding frequencies are shown on the left. Gating strategy is shown in Supplementary Fig. 18. Despite immune activation, DoriVac 100 pmol dose group did not show the same level of PD-1 upregulation as BNT162b2 1 µg dose group. The flow data was analyzed by one-way ANOVA (with correction for multiple comparisons using a Tukey's test) and significance was defined as a multiplicity-adjusted p value less than 0.05 (n=8 for day 22, n=4 for day 50). 'ns' refers to $P > 0.05$; '*' refers to $P \leq 0.05$; '**' refers to $P \leq 0.01$; '***' refers to $P \leq 0.001$.

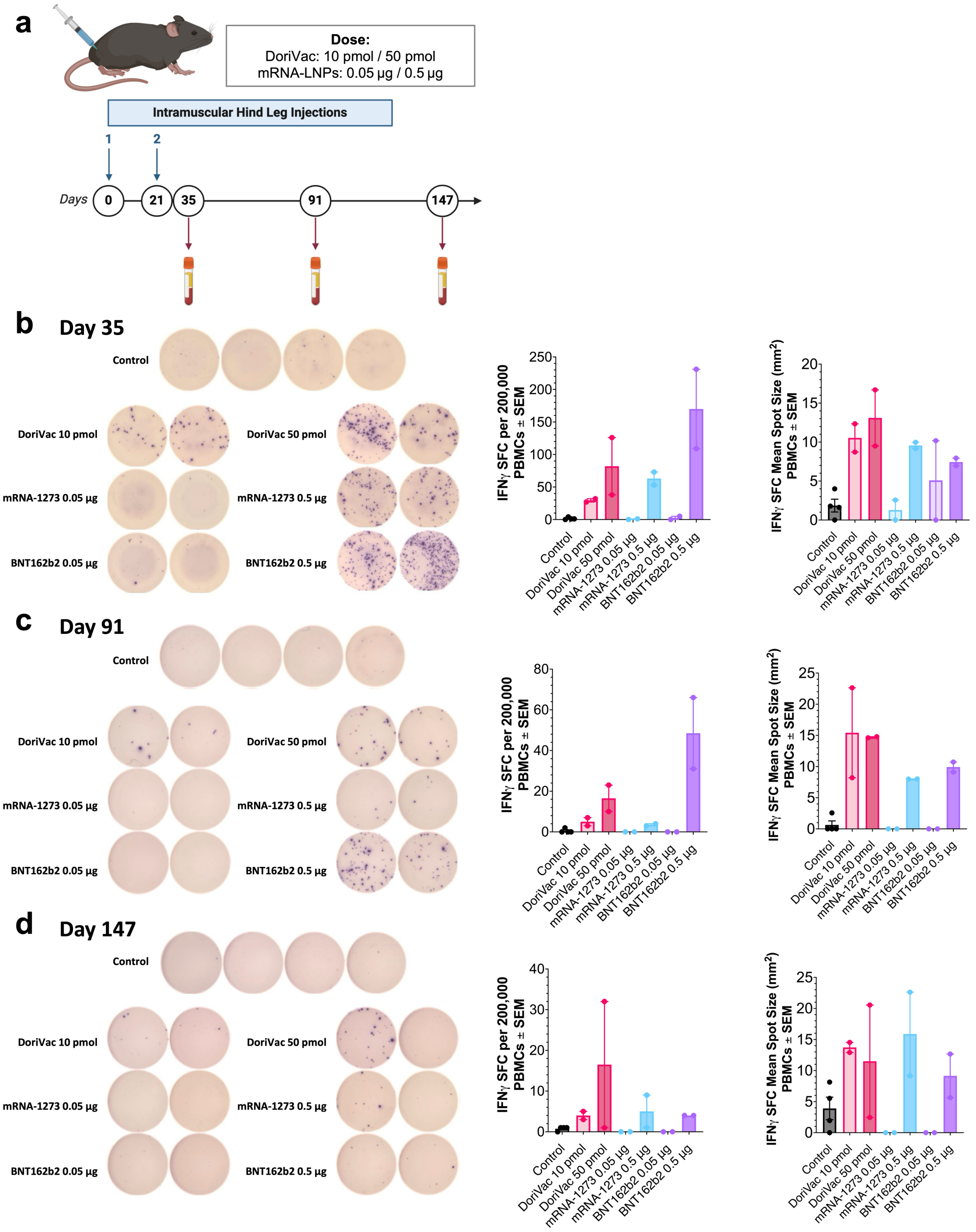

Supplementary Fig. 24 | SARS-CoV-2-specific T cell frequency and spot size following intramuscularly administered treatments as measured by IFNγ ELISpot. a, Schematic delineating the intramuscular vaccine administration protocol for naïve C57BL/6 mice and the data collection timeline. Blood samples were collected on day 35, day 91 and day 147. PBMCs were isolated for ELISpot assay. **b–d,** IFNγ ELISpot demonstrating frequency of antigen-specific PBMCs (n=2, except for control n=4) 35 days (2 weeks), 91 days (10 weeks) and 147 days (21 weeks) after first treatment administration, with accompanying quantification of IFNγ ELISpot SFUs and mean spot sizes. Results show an increase in SARS-COV-2 antigen-specific T cell frequency after treatment with DoriVac compared to the control.

DoriVac also produced larger spot sizes than some mRNA-LNP treated groups. The BNT162b2 0.5 µg dose treatment group experienced a large decrease in spot counts between day 91 and day 147, while DoriVac 50 pmol and mRNA-1273 0.5 µg treatment groups remained fairly consistent in counts, suggesting a more sustained T cell response.

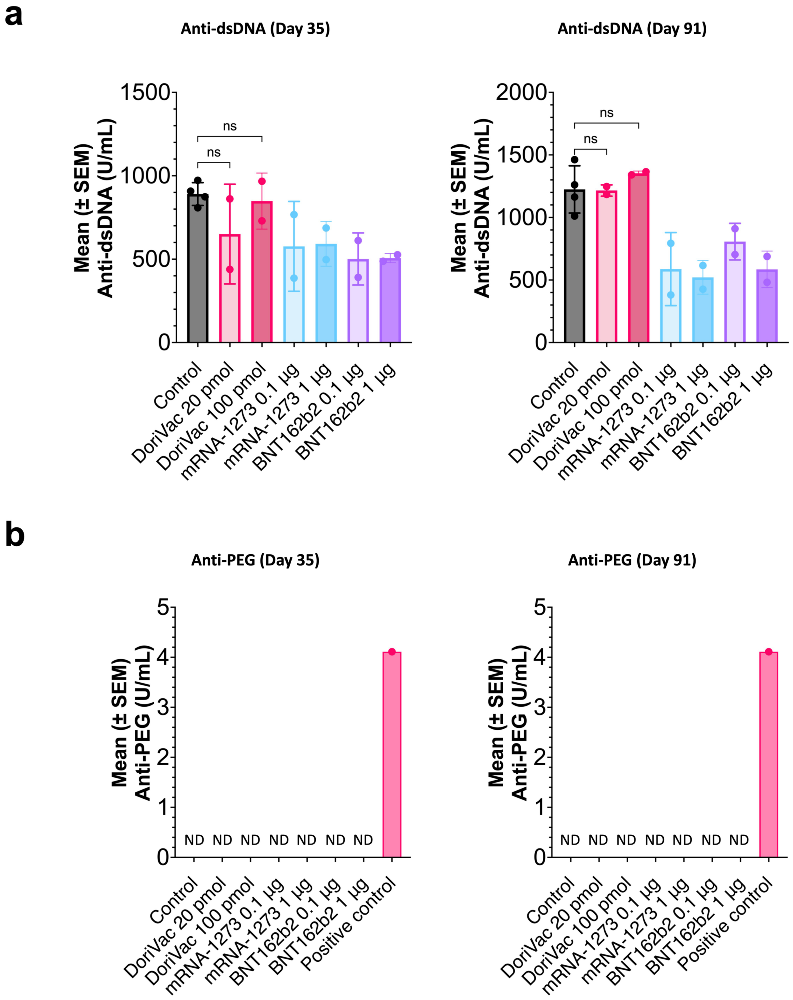

Supplementary Fig. 25 | Anti-dsDNA and anti-PEG IgG production following intramuscularly administered treatments. a, Quantification of relative anti-dsDNA IgG levels in serum samples collected on day 35 and day 91 (2 weeks and 10 weeks after second dose; n=2, except for control n=4; all data points are means of technical duplicates). No significant differences were observed between the control and treated groups (ns). **b,** Quantification of relative anti-PEG IgG levels in serum samples collected on day 35 and day 91 (2 weeks and 10 weeks after second dose; n=2, except for control n=4; all data points are means of technical duplicates). Only the positive control exhibited a measurable anti-PEG IgG level, and its value was in the manufacturer-stated range (within 2.8–5.2 U/mL), confirming assay validity. The "U" in U/mL refers to arbitrary activity units, which are defined by the calibrator curves provided by the manufacturer. All other groups were below the limit of detection (ND). All assays were performed with a 1:100 serum dilution, which is the lowest dilution recommended by the manufacturer (≥1:100). The data were analyzed by one-way ANOVA (with Tukey's correction for multiple comparisons), and significance was defined as a multiplicity-adjusted p value less than 0.05. 'ns' indicates $p > 0.05$.

Supplementary Methods | Determination of Relevant Doses for SARS-CoV-2-Protein-Conjugated DoriVac Compared to mRNA Vaccines

Rationale for Dosing Selection

To benchmark the immunogenicity of the DNA–protein hybrid DoriVac against established vaccine platforms, we compared DoriVac doses with those of commercially available SARS-CoV-2 mRNA–lipid nanoparticle (mRNA-LNP) vaccines. Our goal was to identify physiologically relevant doses suitable for mouse models, enabling meaningful comparisons of immune responses, including dendritic cell activation, T cell priming, and antibody generation.

Reference mRNA Vaccine Dosing Regimens in Mice

Mice have commonly been used as a preclinical model to evaluate mRNA vaccine immunogenicity and protective efficacy prior to human trials. Studies with mRNA-1273 and BNT162b2 have demonstrated robust immune responses at doses ranging from 0.1 µg to 10 µg per injection, depending on the specific aims and formulation.[1-4] For example, Li et al. reported administering 5 µg of an mRNA-based SARS-CoV-2 vaccine intramuscularly on days 0 and 21 to induce robust neutralizing antibody responses and protection in mice, with lower "human-equivalent" doses approximated at 0.2 µg.[1] Corbett et al. explored a range of doses from 0.01 µg to 10 µg for mRNA-1273, concluding that a 1 µg dose induced robust humoral and cellular responses.[2] Thus, typical murine dosing protocols for SARS-CoV-2 mRNA vaccines often center around 0.1–10 µg per dose, depending on experimental design and the desired magnitude of immune responses.[1,2,5,6]

Establishing an Equivalent DoriVac Dose

DoriVac nanoparticle doses were determined based on mole quantities of DNA origami and associated protein antigens. For our SARS-CoV-2 spike-protein-conjugated DoriVac, we employed two primary dose levels—20 pmol and 100 pmol of SQB (DNA origami "square block") constructs per injection. Each DoriVac particle displays multiple spike protein molecules, providing high antigen density. The 20 pmol dose corresponds to approximately 1.2×10^{13} DNA origami particles, and the 100 pmol dose is fivefold higher. Assuming a nominal occupancy of ~5 spike proteins per SQB, the 20 pmol dose presents on the order of ~6×10^{13} spike protein copies. By comparison, mRNA vaccine doses rely on endogenous translation of administered mRNA. Data from mRNA expression studies suggest that 1 µg of mRNA can yield on the order of 10^{13} to 10^{14} spike protein copies per injection, though actual yields vary with mRNA stability, translational efficiency, and delivery conditions.[7-10] Notably, this calculation is an approximation and may not directly translate into equivalent immunogenicity due to differences in mRNA protein translation, antigen processing, and localization. These mRNA copy estimates are derived from publicly available SARS-CoV-2 spike-encoding mRNA vaccine sequences.[11] Using these reference sequences, approximately 4–5 × 10^{11} mRNA molecules are present in 1 µg of mRNA.

Comparisons and Justification of Selected Dose Range

While it is tempting to directly compare the number of antigen molecules delivered by DoriVac with that theoretically produced from mRNA translation, such comparisons must be interpreted with caution. mRNA-LNP vaccines incorporate proprietary structural elements and modified nucleotides to enhance translation and stability, resulting in highly efficient protein production.[1,2,12] Conversely, DoriVac directly delivers pre-formed proteins to antigen-presenting cells. Thus, differences in antigen presentation pathways, kinetics of antigen availability, and immune-processing mechanisms complicate straightforward equivalences between mRNA and protein doses.

Furthermore, while mRNA vaccine doses of 0.1–1 µg are frequently sufficient for robust immunogenicity in mice[1,2], these doses rely on the host's translational machinery and may differ substantially from direct protein delivery platforms. mRNA vaccines continuously produce antigen for as long as the mRNA remains

stable and efficiently translated. In contrast, DoriVac delivers the full antigen complement at once, potentially affecting dose-response kinetics.

Previous studies estimating the number of proteins translated per mRNA molecule have proposed median translation rates and protein half-lives on the order of hours.[7-9] Using a median estimate of ~1,800 proteins generated per mRNA transcript[7], 1 μg of mRNA (comprising ~4–5 × 10^{11} mRNA copies for SARS-CoV-2 spike) might yield on the order of ~8–9 × 10^{14} proteins. Independent *in vitro* data corroborate this scale, measuring ~6 × 10^{14} proteins per μg of spike-encoding mRNA.[13] Such estimates provide useful ballpark figures but are not definitive benchmarks, as *in vivo* translation efficiency is influenced by numerous factors, including cell type, tissue microenvironment, and vaccine formulation.

Our chosen DoriVac doses (20 pmol and 100 pmol) reflect a balance between delivering sufficient antigen for immune priming and maintaining practical nanoparticle concentrations. In our previous study, the 20 pmol DoriVac dose elicited a robust immune response against the antigen and demonstrated favorable efficacy in the tumor model.[14] Analogous to how mRNA vaccine researchers have refined dosing regimens over time, future studies may optimize DoriVac dosing by incorporating a wider range of doses and dosing intervals, as well as evaluating alternative routes of administration.

Conclusions and Future Directions

Determining a "human-equivalent" dose or a direct functional comparison between protein-bearing DNA origami and mRNA vaccines is inherently challenging. Due to the differences in delivery modalities, antigen processing, and immunobiology, numerical equivalences in antigen copy number should serve only as rough guides. The DoriVac doses applied here were chosen to allow a fair comparison against established mRNA-LNP vaccine data. Further exploration—such as parallel dosing studies, long-term immunogenicity assessments, and direct comparisons under identical experimental conditions—will be needed to fine-tune DoriVac dosing strategies and fully elucidate its immunogenic potential relative to current vaccine technologies.

References

1. Li, C., *et al.* Mechanisms of innate and adaptive immunity to the Pfizer-BioNTech BNT162b2 vaccine. *Nat Immunol* **23**, 543-555 (2022).
2. Corbett, K.S., *et al.* SARS-CoV-2 mRNA vaccine design enabled by prototype pathogen preparedness. *Nature* **586**, 567-571 (2020).
3. Vogel, A.B., *et al.* BNT162b vaccines protect rhesus macaques from SARS-CoV-2. *Nature* **592**, 283-289 (2021).
4. Wu, K., *et al.* Serum Neutralizing Activity Elicited by mRNA-1273 Vaccine. *N Engl J Med* **384**, 1468-1470 (2021).
5. Peng, L., *et al.* Variant-specific vaccination induces systems immune responses and potent in vivo protection against SARS-CoV-2. *Cell Rep Med* **3**, 100634 (2022).
6. Peng, L., *et al.* Multiplexed LNP-mRNA vaccination against pathogenic coronavirus species. *Cell Rep* **40**, 111160 (2022).
7. Schwanhausser, B., *et al.* Global quantification of mammalian gene expression control. *Nature* **473**, 337-342 (2011).
8. Wilhelm, M., *et al.* Mass-spectrometry-based draft of the human proteome. *Nature* **509**, 582-587 (2014).
9. Hernandez-Alias, X., Benisty, H., Radusky, L.G., Serrano, L. & Schaefer, M.H. Using protein-per-mRNA differences among human tissues in codon optimization. *Genome Biol* **24**, 34 (2023).
10. Li, J.J., Bickel, P.J. & Biggin, M.D. System wide analyses have underestimated protein abundances and the importance of transcription in mammals. *PeerJ* **2**, e270 (2014).
11. Jeong, D.E., McCoy, M., Artiles, K., Ilbay, O., Fire, A., Nadeau, K., Park, H., Betts, B., Boyd, S., Hoh, R., & Shoura, M. Assemblies of putative SARS-CoV2-spike-encoding mRNA sequences for vaccines BNT-162b2 and mRNA-1273. (2021).
12. Pardi, N., Hogan, M.J., Porter, F.W. & Weissman, D. mRNA vaccines - a new era in vaccinology. *Nat Rev Drug Discov* **17**, 261-279 (2018).

13. Wu, Y., Zhang, H., Meng, L., Li, F. & Yu, C. Comparison of Immune Responses Elicited by SARS-CoV-2 mRNA and Recombinant Protein Vaccine Candidates. *Front Immunol* **13**, 906457 (2022).
14. Zeng, Y.C., *et al.* Fine tuning of CpG spatial distribution with DNA origami for improved cancer vaccination. *Nat Nanotechnol* **19**, 1055-1065 (2024).
