# Supplementary material for: DNA origami vaccine (DoriVac) nanoparticles improve both humoral and cellular immune responses to infectious diseases
Source: bioRxiv. 2024 Jan 2:2023.12.29.573647. Preprint. [Version 1] doi: 10.1101/2023.12.29.573647 (PMC10802255; doi:10.1101/2023.12.29.573647)
Supplement: Supplementary file 1 [file media-1.docx]

**Supplementary Information**

| **Variant** | **Sequence** |
| --- | --- |
| SARS-CoV-2 (Alpha) | GDISGINASVVNIQKEIDRLNEVAKNLNESLIDLQELGKYEQYIK |
| SARS-CoV-2 (Omicron) | GDISGINASVVNIQKEIDRLNEVAKNLNESLIDLQELGKYEQGSG |

**Supplementary Table 1. Differences between HR2 sequences in variants of SARS-CoV-2.** The amino acids that differ between the two variant sequences are colored in red. HR2 peptide is highly conserved, compared to many epitopes within the spike region, which renders the effectiveness of HR2-based SARS-CoV-2 vaccines less susceptible to antigenic drift.

**Supplementary Table 2. Peptide sequences and relevant properties.** Peptides were purchased with N-terminal azide modifications from IDT. The azide modifications enable a click-chemistry reaction between the azide-modified peptide and a DBCO-modified oligonucleotide. A negative value in the Grand AVerage of hydropathY (GRAVY) score indicates peptides that are net hydrophilic at pH 7.

**Supplementary Table 3. Peptide conjugation efficiency.** Peptide conjugation efficiency was determined by using the band intensity of a DNase I-digested infectious disease SQB, as calculated by ImageJ, and comparing it with the band intensity of the theoretical amount of peptide.

**Supplementary Table 4. Antibodies used for dendritic-cell (DC) flow cytometry.** These antibodies were grouped into different panels depending on the experimental goals. 

**Supplementary Table 5. Antibodies used for B cells flow cytometry.** These antibodies were grouped into different panels depending on the experimental goals. 

**Supplementary Table 6. Antibodies used for T cells flow cytometry.** These antibodies were grouped into different panels depending on the experimental goals. 

**Supplementary Table 7. Predicted murine MHC epitopes of the SARS-CoV-2 HR2 peptide and associated MHC binding.** NetMHCpan-4.1 was used to predict the top eight murine MHC-I and MHC-II epitopes of each HR2 peptide. The default cutoff for MHC binders was used as described by NetMHCpan^58^. A lower rank score for the peptide is associated with stronger binding to MHC. 0.5% was defined as the cutoff for ‘strong’ MHC-I binders while 2% was defined as the cutoff for ‘weak’ MHC-I binders. Any peptides that had a rank score >2% were assumed to not bind to MHC-I. 2% was defined as the cutoff for ‘strong’ MHC-II binders while 10% was defined as the cutoff for ‘weak’ MHC-II binders. Any peptides that had a rank score >10% were assumed to not bind to MHC-II. 

**Supplementary Table 8. Predicted murine MHC epitopes of the HIV HR2 peptide and associated MHC binding.** NetMHCpan-4.1 was used to predict the top eight murine MHC-I and MHC-II epitopes of each HR2 peptide. The default cutoff for MHC binders was used as described by NetMHCpan^58^. A lower rank score for the peptide is associated with stronger binding to MHC. 0.5% was defined as the cutoff for ‘strong’ MHC-I binders while 2% was defined as the cutoff for ‘weak’ MHC-I binders. Any peptides that had a rank score >2% were assumed to not bind to MHC-I. 2% was defined as the cutoff for ‘strong’ MHC-II binders while 10% was defined as the cutoff for ‘weak’ MHC-II binders. Any peptides that had a rank score >10% were assumed to not bind to MHC-II. 

**Supplementary Table 9. Predicted murine MHC epitopes of the Ebola HR2 peptide and associated MHC binding.** NetMHCpan-4.1 was used to predict the top eight murine MHC-I and MHC-II epitopes of each HR2 peptide. The default cutoff for MHC binders was used as described by NetMHCpan^58^. A lower rank score for the peptide is associated with stronger binding to MHC. 0.5% was defined as the cutoff for ‘strong’ MHC-I binders while 2% was defined as the cutoff for ‘weak’ MHC-I binders. Any peptides that had a rank score >2% were assumed to not bind to MHC-I. 2% was defined as the cutoff for ‘strong’ MHC-II binders while 10% was defined as the cutoff for ‘weak’ MHC-II binders. Any peptides that had a rank score >10% were assumed to not bind to MHC-II. 

**Supplementary Table 10. Summary Table of ‘strong’ and ‘weak’ MHC binding epitopes for various HR2 peptides.** NetMHCpan-4.1 was used to predict the top eight murine MHC-I and MHC-II epitopes of each HR2 peptide. The default cutoff for MHC binders was used as described by NetMHCpan^58^. A lower rank score for the peptide is associated with stronger binding to MHC. 0.5% was defined as the cutoff for ‘strong’ MHC-I binders while 2% was defined as the cutoff for ‘weak’ MHC-I binders. Any peptides that had a rank score >2% were assumed to not bind to MHC-I. 2% was defined as the cutoff for ‘strong’ MHC-II binders while 10% was defined as the cutoff for ‘weak’ MHC-II binders. Any peptides that had a rank score >10% were assumed to not bind to MHC-II. 

**Supplementary Table 11. Predicted human HLA epitopes of the SARS-CoV-2 HR2 peptide and associated HLA binding.** NetMHCpan-4.1 was used to predict the top eight human HLA-A*02:01 and HLA-DRB1*01:11 epitopes of each HR2 peptide. The default cutoff for HLA binders was used as described by NetMHCpan^58^. A lower rank score for the peptide is associated with stronger HLA binding. 0.5% was defined as the cutoff for ‘strong’ HLA-A*02:01 binders while 2% was defined as the cutoff for ‘weak’ HLA-A*02:01 binders. Any peptides that had a rank score >2% were assumed to not bind to HLA-A*02:01. 2% was defined as the cutoff for ‘strong’ HLA-DRB1*01:11 binders while 10% was defined as the cutoff for ‘weak’ HLA-DRB1*01:11 binders. Any peptides that had a rank score >10% were assumed to not bind to HLA-DRB1*01:11. HLA-A*02:01 and HLA-DRB1*01:11 were chosen as representative human HLAs, as they are the most well-studied HLAs of each class.

**Supplementary Table 12. Predicted human HLA epitopes of the HIV HR2 peptide and associated HLA binding.** NetMHCpan-4.1 was used to predict the top eight human HLA-A*02:01 and HLA-DRB1*01:11 epitopes of each HR2 peptide. The default cutoff for HLA binders was used as described by NetMHCpan^58^. A lower rank score for the peptide is associated with stronger HLA binding. 0.5% was defined as the cutoff for ‘strong’ HLA-A*02:01 binders while 2% was defined as the cutoff for ‘weak’ HLA-A*02:01 binders. Any peptides that had a rank score >2% were assumed to not bind to HLA-A*02:01. 2% was defined as the cutoff for ‘strong’ HLA-DRB1*01:11 binders while 10% was defined as the cutoff for ‘weak’ HLA-DRB1*01:11 binders. Any peptides that had a rank score >10% were assumed to not bind to HLA-DRB1*01:11. HLA-A*02:01 and HLA-DRB1*01:11 were chosen as representative human HLAs, as they are the most well-studied HLAs of each class.

**Supplementary Table 13. Predicted human HLA epitopes of the Ebola HR2 peptide and associated HLA binding.** NetMHCpan-4.1 was used to predict the top eight human HLA-A*02:01 and HLA-DRB1*01:11 epitopes of each HR2 peptide. The default cutoff for HLA binders was used as described by NetMHCpan^58^. A lower rank score for the peptide is associated with stronger HLA binding. 0.5% was defined as the cutoff for ‘strong’ HLA-A*02:01 binders while 2% was defined as the cutoff for ‘weak’ HLA-A*02:01 binders. Any peptides that had a rank score >2% were assumed to not bind to HLA-A*02:01. 2% was defined as the cutoff for ‘strong’ HLA-DRB1*01:11 binders while 10% was defined as the cutoff for ‘weak’ HLA-DRB1*01:11 binders. Any peptides that had a rank score >10% were assumed to not bind to HLA-DRB1*01:11. HLA-A*02:01 and HLA-DRB1*01:11 were chosen as representative human HLAs, as they are the most well-studied HLAs of each class.

**Supplementary Table 14. Summary Table of ‘strong’ and ‘weak’ HLA binding epitopes for various HR2 peptides.** NetMHCpan-4.1 was used to predict the top eight human HLA-A*02:01 and HLA-DRB1*01:11 epitopes of each HR2 peptide. The default cutoff for HLA binders was used as described by NetMHCpan^58^. A lower rank score for the peptide is associated with stronger HLA binding. 0.5% was defined as the cutoff for ‘strong’ HLA-A*02:01 binders while 2% was defined as the cutoff for ‘weak’ HLA-A*02:01 binders. Any peptides that had a rank score >2% were assumed to not bind to HLA-A*02:01. 2% was defined as the cutoff for ‘strong’ HLA-DRB1*01:11 binders while 10% was defined as the cutoff for ‘weak’ HLA-DRB1*01:11 binders. Any peptides that had a rank score >10% were assumed to not bind to HLA-DRB1*01:11. HLA-A*02:01 and HLA-DRB1*01:11 were chosen as representative human HLAs, as they are the most well-studied HLAs of each class.

**Supplementary Figure 1. HR2-functionalized DoriVac large scale TEM image. (A)** Negative stain TEM image of DoriVac nanoparticles demonstrated that the nanoparticles were monodispersed, further supporting the agarose gel results. **(B)** Negative stain TEM image of HIV nanoparticles demonstrated that the nanoparticles were majority monodispersed, with some dimers observed, further supporting the agarose gel results. **(C)** Negative stain TEM image of Ebola nanoparticles demonstrated that the nanoparticles were majority monodispersed, with some dimers observed, further supporting the agarose gel results. 

**Supplementary Figure 2. B cell gating strategies.** B cells were analyzed using flow cytometry. This figure demonstrates the gating strategy to analyze B cells from frozen PBMC samples. 

**Supplementary Figure 3. Additional data demonstrating robust humoral responses for B cells. (A)** The CD19^+^ B cell population in the blood on Day 21, as determined by flow cytometry, showed no significant difference after bolus vaccine treatment compared with DoriVac treatment. **(B)** The B220^+^ B cell population in the blood on Day 21, as determined by flow cytometry, showed minimal difference after bolus vaccine treatment compared with DoriVac treatment. Data are represented as mean ± SD. The flow data were analyzed by multiple unpaired t-tests and significance was defined as a two-tailed p value less than 0.05. ‘*’ refers to p ≤ 0.05; ‘**’ refers to p ≤ 0.01; ‘***’ refers to p≤ 0.001; ‘****’ refers to p ≤ 0.0001.

**Supplementary Figure 4. Additional data demonstrating robust humoral responses for B cells.** (**A)** DoriVac treatment enhanced SARS-CoV-2 peptide-specific IgG antibody production in the plasma, as determined by ELISA assay, after two doses of the vaccine (on Day 35) compared to a bolus vaccine of free peptide and free CpG. **(B)** DoriVac treatment enhanced HIV-peptide-specific IgG antibody production in the plasma, as determined by ELISA assay, after two doses of the vaccine (on Day 35) compared to a bolus vaccine. **(C)** DoriVac treatment enhanced Ebola-peptide-specific IgG antibody production in the plasma, as determined by ELISA assay, after two doses of the vaccine (on Day 35) compared to a bolus vaccine. Data has been normalized. Data are represented as mean ± SD. The ELISA data was analyzed by one-way ANOVA (with correction for multiple comparisons using a Tukey’s test) and significance was defined as a multiplicity-adjusted p value less than 0.05 (n=8). ‘*’ refers to P ≤ 0.05; ‘**’ refers to P ≤ 0.01. ‘***’ refers to P ≤ 0.001.

**Supplementary Figure 5. DC gating strategies for lymph node samples.** Dendritic cells were analyzed using flow cytometry. This figure demonstrates the gating strategy to analyze dendritic cells collected from the lymph nodes.

**Supplementary Figure 6. Additional data demonstrating DC responses in the splenocytes on Day 21.** Spleens were collected on Day 21 after two doses of treatment and processed into single-cell suspensions for flow cytometry. **(A)** Percentages of CD11c^+^ CD86^+^, as determined by flow cytometry, in the splenocyte population (n=4). DoriVac demonstrated a significant increase in this activated DC population compared to bolus-vaccine treatment.  **(B)** Percentages of CD11c^+^ Gr-1^+^ DCs, as determined by flow cytometry, in the splenocyte population (n=4). DoriVac treatment demonstrated an increase in this plasmacytoid DC (pDC) – like subpopulation compared to bolus-vaccine treatment. **(C)** Percentages of CD40^+ ^DEC205^+^ DCs, as determined by flow cytometry, in the splenocytes (n=4). DoriVac treatment demonstrated an increase in this activated, endocytic subpopulation compared to bolus-vaccine treatment. **(D)** Percentages of MHC-II^+^ DCs, as determined by flow cytometry, in the draining lymph node (n=4). DoriVac treatment demonstrated a significant increase in this activated DC population compared to bolus-vaccine treatment. (**E)** DoriVac treatment led to a notable increase in the CD11c^+^ dendritic-cell population, as determined by flow cytometry. **(F)** The lymphocytes were more activated (as indicated by upregulation of CD40^+^) after DoriVac treatment compared to bolus vaccine treatment, as determined by flow cytometry. **(G)** An increase in the PD-L1^+^ subpopulation was observed after DoriVac treatment, compared to bolus-vaccine treatment, as determined by flow cytometry. Data are represented as mean ± SD. The flow data were analyzed by multiple unpaired t-tests and significance was defined as a two-tailed p value less than 0.05. ‘*’ refers to p ≤ 0.05; ‘**’ refers to p ≤ 0.01; ‘***’ refers to p≤ 0.001; ‘****’ refers to p ≤ 0.0001.

**Supplementary Figure 7. Co-delivery of CpG and peptide on SQB is critical to robust DC responses.** Half of the mice were sacrificed on Day 21 and draining lymph nodes were collected. **(A)** Percentages of human pDC-like (CD11c^+^ Gr-1^+^) DCs in the draining lymph node (n=4), as determined by flow cytometry. DoriVac treatment demonstrated a significant increase in this plasmacytoid DC (pDC) – like subpopulation compared to treatment with the CpG SQB and HIV peptide, administered as a solution. **(B)**  Percentages of activated cDCs (CD11c^+^ CD86^+^) in the draining lymph node, as determined by flow cytometry. DoriVac treatment showed significant activation compared to the bolus vaccine and slightly more than the DNA origami SQB and HIV peptide administered as separate components. **(C)** Percentages of CD40^+^ DEC205^+^ DCs in the draining lymph node (n=4), as determined by flow cytometry. The DoriVac treatment demonstrated a significant increase in this activated, endocytic subpopulation compared to bolus vaccine treatment and more than the DNA origami SQB and HIV peptide administered as separate components. **(D)** Percentages of human pDC-like DCs in the splenocytes on Day 21 (n=4), as determined by flow cytometry. DoriVac treatment shows a significant increase compared to the bolus control. **(E)** Percentages of activated cDCs in the splenocyte population, as determined by flow cytometry. DoriVac induced a significant increase in this population compared to the bolus control. **(F)** Percentages of activated, endocytic DCs in the splenocytes population, as determined by flow cytometry. DoriVac showed significant increase in this population compared to the DNA origami SQB and HIV peptide delivered separately. **(G)** On Day 35, two weeks after the second dose of treatment, the remaining mice were sacrificed and their lymph nodes were harvested. The activated cDC population was significantly increased in the DoriVac group, as determined by flow cytometry, when compared to the control and the DNA origami SQB and HIV peptide group, although lower than the untreated control, suggesting that activation returns to baseline around 2 weeks after the final dose.  Data are represented as mean ± SD. The flow data was analyzed by one-way ANOVA (with correction for multiple comparisons using a Tukey’s test) and significance was defined as a multiplicity-adjusted p value less than 0.05 (n=4). ‘*’ refers to P ≤ 0.05; ‘**’ refers to P ≤ 0.01; ‘***’ refers to P ≤ 0.001; ‘****’ refers to P ≤ 0.0001.

**Supplementary Figure 8. Additional data on antigen-specific T cell responses on Day 21 and 28. (A)** Interferon-gamma (IFNγ) ELIspot demonstrating frequency of antigen-specific splenocytes on Day 21. Minimal difference is observed between the bolus-vaccine treated group and the DoriVac-treated group. **(B)** IFNγ ELIspot demonstrating frequency of antigen-specific PBMCs on Day 28. There is a notable increase in the frequency of SARS-CoV-2 antigen-specific T cells. 

**Supplementary Figure 9. T cell gating strategies.** T cells were analyzed using flow cytometry. This figure demonstrates the gating strategy to analyze T cells in lymph nodes and spleen. Images shown are from the lymph nodes.

**Supplementary Figure 10. Co-delivery of CpG and antigen peptide is essential to robust T cell responses. (A)** IFN-γ^+^ CD8^+^ T cells in the lymph node (LN) were upregulated on Day 21 in the DoriVac treatment group, compared to the bolus vaccine or the DNA origami SQB with HIV peptide groups, as determined by flow cytometry. **(B)** CD107a^+^ CD8^+^ cytotoxic T cells in the LN were upregulated on Day 21 in the DoriVac treatment group, compared to the bolus vaccine or the DNA origami SQB with HIV peptide groups, as determined by flow cytometry. **(C)** CD69^+^ CD8^+^ activated T cells in the LN showed upregulation on Day 21 after treatment with DoriVac, as determined by flow cytometry. **(D)** IFN-γ^+^ CD4^+^ T cells in the LN were upregulated in the context of DoriVac treatment group on Day 21, compared to the DNA origami SQB with HIV peptide group, as determined by flow cytometry. **(E)** PD-1^+^ CD4^+^ T cells in the LN were upregulated in the context of the DoriVac treated group on Day 21, compared to the bolus vaccine and DNA origami SQB with HIV peptide group, as determined by flow cytometry.  Data are represented as mean ± SD. The flow data was analyzed by one-way ANOVA (with correction for multiple comparisons using a Tukey’s test) and significance was defined as a multiplicity-adjusted p value less than 0.05 (n=4). ‘*’ refers to P ≤ 0.05; ‘**’ refers to P ≤ 0.01.
